# Synthesis and Structure–Activity Relationships of a New Class of Oxadiazoles Targeting DprE1 as Antitubercular Agents

**DOI:** 10.1021/acsmedchemlett.3c00295

**Published:** 2023-08-15

**Authors:** Veena D. Yadav, Helena I. Boshoff, Lena Trifonov, Jose Santinni O. Roma, Thomas R. Ioerger, Clifton E. Barry, Sangmi Oh

**Affiliations:** † Tuberculosis Research Section, Laboratory of Clinical Immunology and Microbiology, National Institute of Allergy and Infectious Diseases (NIAID), National Institutes of Health (NIH), Bethesda, Maryland 20892, United States; ‡ Department of Computer Science and Engineering, Texas A&M University, College Station, Texas 77843, United States

**Keywords:** 1,3,4-oxadiazole, structure−activity relationship, mycobacteria, tuberculosis, multidrug resistance, high-throughput screening

## Abstract

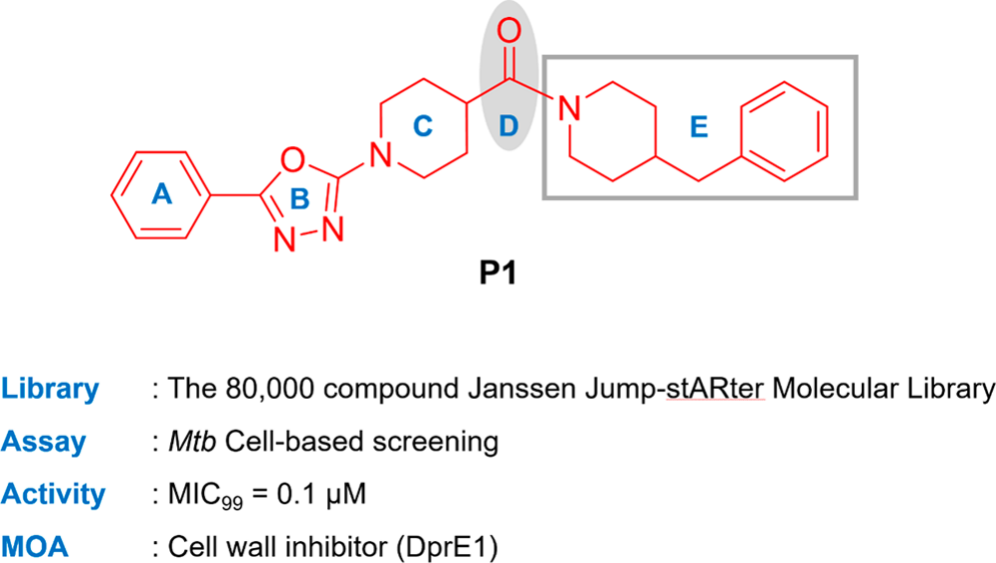

The continuing prevalence of drug-resistant tuberculosis threatens global TB control programs, highlighting the need to discover new drug candidates to feed the drug development pipeline. In this study, we describe a high-throughput screening hit (4-benzylpiperidin-1-yl)(1-(5-phenyl-1,3,4-oxadiazol-2-yl)piperidin-4-yl)methanone (**P1**) as a potent antitubercular agent. Structure–activity guided synthesis led to the discovery of several analogs with high *in vitro* potency. **P1** was found to have promising potency against many drug-resistant strains, as well as drug-susceptible clinical isolates. It also showed cidality against *Mtb* growing in host macrophages. Whole genome sequencing of genomic DNA from resistant mutants raised to **P1** revealed mutations in decaprenylphosphoryl-β-d-ribose 2′-oxidase (DprE1). This novel oxadiazole scaffold expands the set of chemical tools for targeting a well-validated pathway to treat tuberculosis.

The goal of the World Health Organization to eliminate tuberculosis (TB) by the year 2050 was undermined by the COVID-19 pandemic with increases in numbers of cases, numbers of deaths, and incidence of drug resistant cases recorded from 2020 to 2022.^1,2^ Continuing rise and spread of drug resistant tuberculosis is a global health threat and increases pressure on drug discovery programs to deliver new candidates to expand the TB drug development pipeline.

Since the mycobacterial cell wall is an essential protective barrier against various cellular stresses and important for virulence in the host, it has been the focus of countless efforts to identify small-molecule inhibitors of enzymes involved in its biogenesis.^3^ The vulnerability of drug targets in the cell wall has been validated by the chemotherapeutic efficacy of several antitubercular drugs in patients, including isoniazid and ethambutol, which are part of the first-line antitubercular drug regimen. Many antitubercular agents inhibiting mycolyl-arabinogalactan-peptidoglycan (mAGP) biogenesis have been introduced of which several have demonstrated therapeutic potential based on their *in vivo* efficacy.^4,5^ The well-studied protein targets include not only the NADH-dependent enoyl-acyl carrier protein reductase (InhA) which is the target of isoniazid but also the mycobacterial membrane protein Large 3 (MmpL3) and the decaprenylphosphoryl-β-d-ribose 2′-oxidase (DprE1). As an inhibitor of MmpL3, ethambutol-inspired SQ109 is in clinical trials with its bactericidal activity against drug-susceptible and drug-resistant TB strains ascribed to its inhibition of translocation of trehalose monomycolate (TMM) across the membrane.^6^ In the case of DprE1 inhibitors, four drug candidates with three scaffolds, BTZ-043, macozinone (PBTZ-169), quabodepistat (OPC-167832), and TBA-7371, are in the different phases of clinical trials.^7^ BTZ-043 and macozinone are prodrugs with a benzothiazinone core, which are activated by DprE1 resulting in covalent suicide inhibition of the enzyme.^8^ In contrast, quabodepistat and TBA-7371 are noncovalent inhibitors of DprE1 with different structural features, a carbostyril and a 1,4-azaindole scaffold, respectively, and good therapeutic profiles.^9^ Since several structurally diverse inhibitors of MmpL3 and DprE1 have been identified, of which several are in different stages of the TB drug development pipeline, the concern has been raised that these are promiscuous drug targets.^10^ Nevertheless, the inhibition of bacterial growth along with the *in vivo* efficacy of several of these inhibitors confirms the vulnerability of these targets and supports efforts to assess and develop such inhibitors.

In an effort to discover new antimicrobial agents, screening of the 80,000 compound Janssen Jump-stARter Molecular Library^11^ against *Mtb* growing on glucose or on dipalmitoylphosphatidylcholine/cholesterol as carbon sources yielded a 1,3,4-oxadiazole-containing hit, **P1** (Figure 1), which inhibited *Mtb* growth under both growth conditions. The oxadiazole core represents a promising new chemotype for antitubercular drug discovery and has served as a privileged scaffold in several drug discovery programs serving as linker or a key pharmacophoric moiety in several approved as well as investigational stage drugs.^12^ For example, a new class of non-β-lactam antibiotics targeting cell-wall biosynthesis contained an oxadiazole moeity.^13^ In addition, an oxadiazole can serve as a bioisosteric substitution for the hydrazide moiety, such as that found in the first-line anti-TB drug isoniazid.^14^ Some oxadiazole derivatives have also been reported to interact with novel *Mtb* targets.^12,14,15^ Previously, GSK reported the SAR of a 1,3,4-thiadiazole-containing hit (GSK710) that was selected from the TB-set with enzymatic activity against DprE1, which shared high degree of structural similarity with **P1**.^16,17^ Based on these favorable properties, **P1** was selected for formal hit assessment. Determination of the concentration required to fully inhibit growth of *Mtb* (minimum inhibitory concentration, MIC) confirmed that the compound was active, with sub-micromolar MIC, against *Mtb* growing on both glycolytic (glucose) and gluconeogenic (dipalmitoylphosphatidylcholine and cholesterol) carbon sources (Table 1). Profiling of the compound in several reporter assays revealed that the compound perturbed cell-wall synthesis, but was unlikely to result in DNA damage or respiratory inhibition (Table 1), based on phenotypic assays for (1) inhibition of cell wall mycolyl-arabinogalactan biosynthesis, (2) respiration through the bc_1_ menaquinol-cytochrome c oxidoreductase, or (3) DNA damage (Table 1).^18,19^

**Figure 1:**
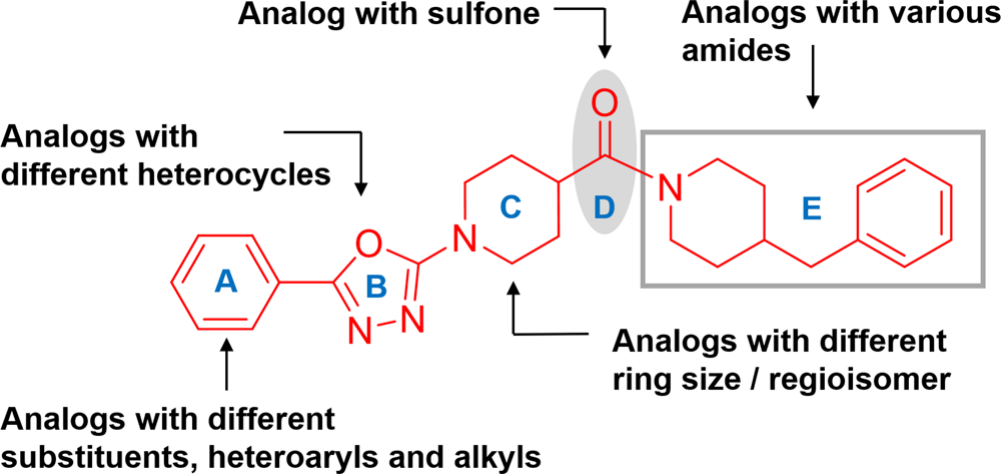
Chemical structure of hit compound **P1** and its derivatization for the SAR study.

**Table 1:** Biological Screening of **P1**

	MIC (μM)					Reporter gene assay	
Hit	7H9^a^	GBSA^b^	DPPC^c^	Chol^d^	CydKO^e^	cell wall^f^	DNA damage^g^
**P1**	0.78	0.78	0.6	0.78	0.6	yes	no
INH	0.2	0.3	0.2	0.39	0.39	ND^h^	no

a MIC of compound against *Mtb* strain H37Rv in Middlebrook 7H9/ADC/Tween 80.

b MIC of compound against *Mtb* strain H37Rv in Middlebrook 7H9/BSA/Tyloxapol with glucose (GBSA) or dipalmitoylphosphatidylcholine (DBSA) as the carbon source.

c MIC of compound against *Mtb* strain H37Rv in Middlebrook 7H9/DPPC/cholesterol/BSA/Tyloxapol.

d MIC of compound against *Mtb* strain H37Rv in Middlebrook 7H9/cholesterol/BSA/Tyloxapol.

e MIC of compound against *Mtb* strain H37Rv in Middlebrook 7H9/ADC/Tween 80 with *cydC::aph*.

f Induction of the cell wall responsive *ini*BAC promoter as measured using the *pini*B-LUX reporter strain.

g Induction of the DNA damage responsive *rec*A and *rad*A reporters using the *prec*A-LUX and *prad*A-LUX reporter strains.

h Not determined.

We investigated the structure–activity relationships (SAR) of the initial hit **P1** in five regions as shown in Figure 1. General synthesis of 1,3,4-oxadiazole analogs (**P2**–**P5**, **P8**–**P10**, **P12**–**P30**) including **P1** started from a coupling reaction of commercially available 2-(methylsulfonyl)-1,3,4-oxadiazoles (**1**) with appropriate cyclic amines (**2**) to generate intermediate **3**. After saponification of the ester group, the resulting carboxylic acid was coupled with appropriate amine to form the desired product (Scheme 1).^20−23^

**Scheme 1:**
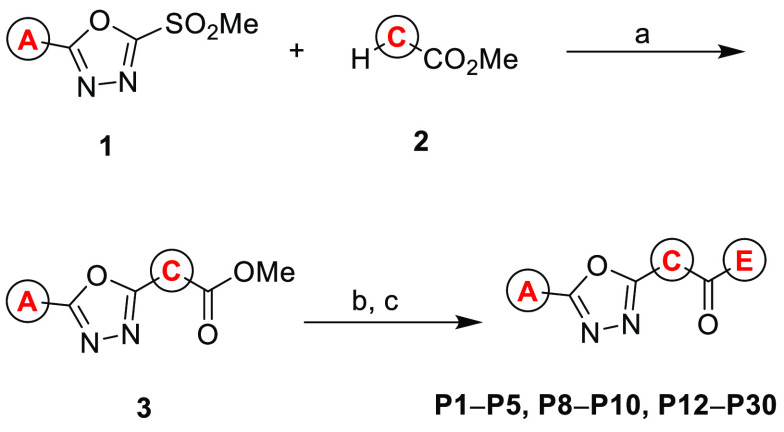
General Synthetic Scheme of **P1** and Its Derivatives **P2–P5**, **P8–P10**, and **P12–P30** Reagents and conditions: (a) K_2_CO_3_ (2.0 equiv), DMF, rt, 18 h; (b) NaOH (2.5 equiv), MeOH, 50 °C, 1.5 h; (c) EH, EDC, HOBt, DIPEA, 0 °C to rt, DCM, 9–14 h, 75–81%; Letters A, C, and E in red indicate each moiety depicted in Figure 1.

The 1,3,4-thiadiazole and 1,2,4-oxadiazole-based analogs (**P6** and **P7**) were prepared from commercially available 2-chloro-5-phenyl-1,3,4-thiadiazole (**4a**) and 3-bromo-5-phenyl-1,2,4-oxadiazole (**4b**) via nucleophilic substitution with piperidine-4-carboxylate to afford the corresponding intermediates **5a** and **5b**.^24^ Further basic hydrolysis afforded the corresponding acids, which were used directly for further EDC coupling with amine 4-benzylpiperidine to afford compounds **P6** and **P7** (Scheme 2). **P11** with a sulfonyl linker instead of the carbonyl group was prepared from commercially available Boc-protected 4-(chlorosulfonyl)piperidine (**6**) and 4-benzylpiperidine. After the coupling reaction followed by deprotection of the Boc group, the same amide coupling reaction with 4-benzylpiperidine afforded the final compound **P11** (Scheme 3).

**Scheme 2:**
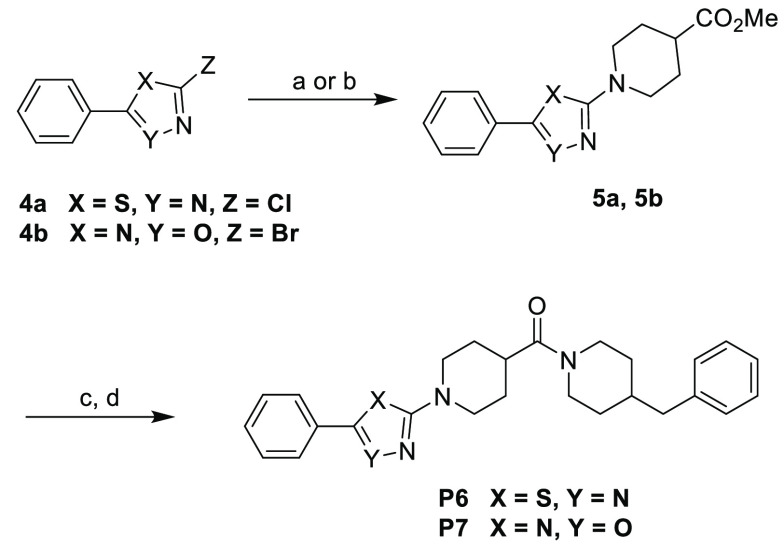
Synthesis of **P6** and **P7** Reagents and conditions: (a) methylpiperidine-4-carboxylate (1.2 equiv), K_2_CO_3_ (2.0 equiv), DMF, 110 °C, 19 h, 81%; (b) methylpiperidine-4-carboxylate (1.2 equiv), K_2_CO_3_ (2.0 equiv), DMF, 55 °C, 25 h, 42%; (c) NaOH (2.5 equiv), MeOH, 50 °C, 1.5 h; (d) 4-benzylpiperidine, EDC, HOBt, DIPEA, 0 °C to rt, DCM, 12 h, **P6** (**7**9%) and **P7** (81%).

**Scheme 3:**
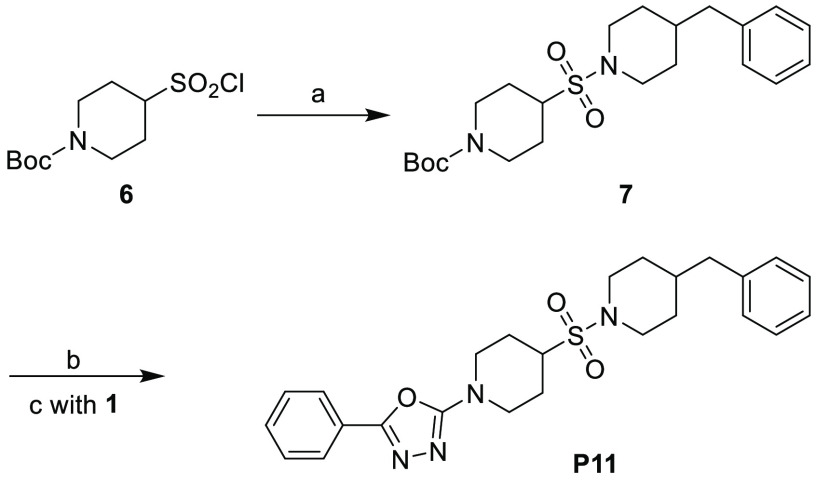
Synthesis of **P11** Reagents and conditions: (a) 4-benzylpiperidine (1.2 equiv), NEt_3_ (2.0 equiv), DCM, 2 h, 72%; (b) TFA/DCM (1/4), 2 h; (c) K_2_CO_3_ (2.0 equiv), DMF, rt, 18 h, 52%.

This focused library of 29 analogs was evaluated for antitubercular activity by MIC determination against *Mtb* in two different media, specifically media with or without albumin in the presence of glucose as carbon source (GBSA and GCas, respectively) to assess the effect of protein binding on the activity. The frontline antitubercular agent, isoniazid, served as the positive control (Tables 2 and 3). Compounds **P2**–**P5** were synthesized to evaluate the importance of the A ring on the left-hand side. Compared to the initial hit, **P1** (MIC = 0.1 μM), which has a phenyl ring at this position, replacement with a methyl group in **P2** caused the complete loss of activity, whereas a pyridine in **P3** caused a 4-fold increase in MIC value (0.39 μM), indicating that the hydrophobic and aromatic phenyl ring was essential for activity. Substitution of the *para*-position of the A ring with a small, electron-withdrawing fluoro substituent (**P4**) was tolerated, but a trifluoromethyl substituent (**P5**) at the same position eliminated its activity against *Mtb*, showing the importance of the substituent size on the A ring.

**Table 2:**
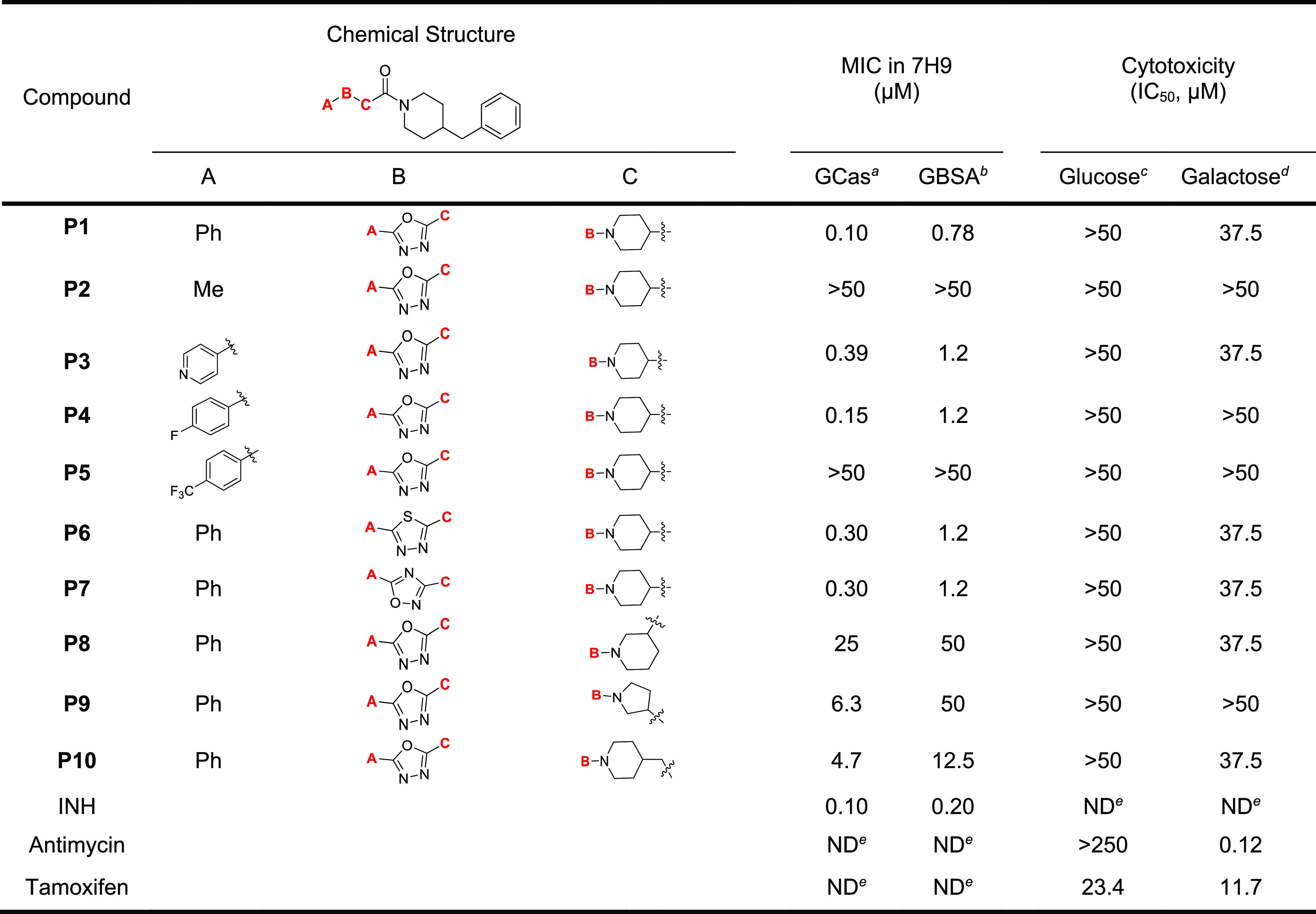
Structural Aspects and Antitubercular Activities of **P1**–**P10**

a MIC of compounds tested against *Mtb* H37Rv in Middlebrook 7H9/glucose/casitone/tyloxapol.

b MIC of compounds tested against *Mtb* H37Rv in 7H9/glucose/BSA/tyloxapol.

c Cytotoxicity of compound tested against HepG2 cells in DMEM/10% FBS supplemented with glucose.

d Cytotoxicity of compound tested against HepG2 cells in DMEM/10% FBS supplemented with galactose.

e Not determined.

**Table 3:**
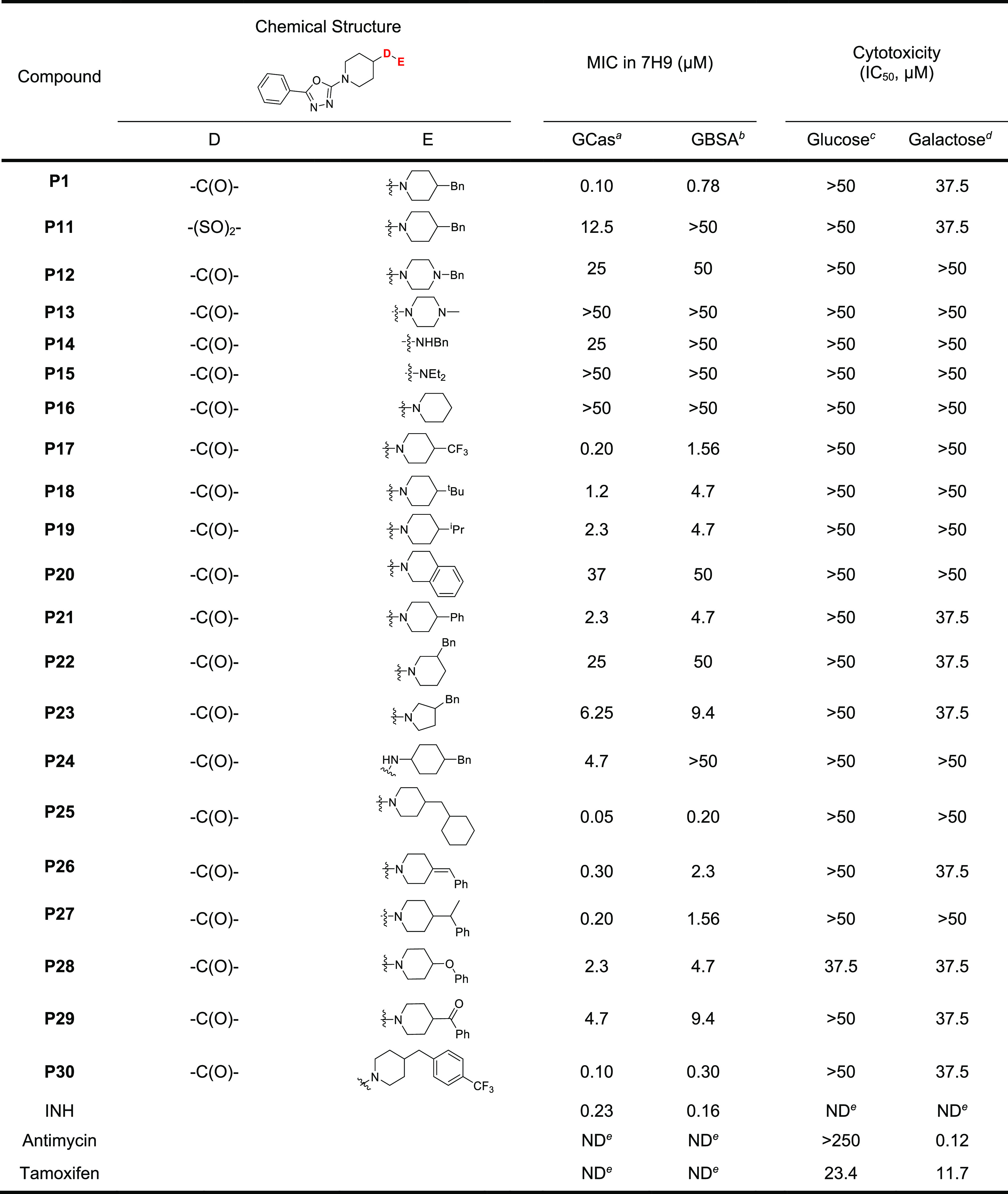
Structural Aspects and Antitubercular Activities of **P11**–**P30**

a MIC of compounds tested against *Mtb* H37Rv in Middlebrook 7H9/glucose/casitone/tyloxapol.

b MIC of compounds tested against *Mtb* H37Rv in 7H9/glucose/BSA/tyloxapol.

c Cytotoxicity of compound tested against HepG2 cells in DMEM/10% FBS supplemented with glucose.

d Cytotoxicity of compound tested against HepG2 cells in DMEM/10% FBS supplemented with galactose.

e Not determined.

The importance of the heteroatoms in the oxadiazole ring (core B) was explored via synthesis of **P6** whereas the regioisomeric contribution of the atoms was evaluated through synthesis of **P7**. Exchange of the oxygen atom in the oxadiazole ring with a sulfur atom to form a thiadiazole ring (**P6**) and modification of the 1,3,4-oxadiazole to a 1,2,4-oxadiazole moiety (**P7**) were tolerated (MICs = 0.3 μM) with only a slight increase in MIC compared to **P1**. These results suggest that three heteroatoms with a lone pair of electrons are important by functioning as hydrogen-bond acceptors within the binding pocket of the target protein, although the limited number of compounds evaluated in the assessment of core B limits any strong conclusions. To explore the role of the piperidine ring in part C, a different regioisomer (**P8**), a different ring size with a pyrrolidine analog (**P9**), and a methyl insertion between the piperidine ring C and the carbonyl carbon (**P10**) were synthesized, all of which resulted in loss of potency. For all active analogs described in Table 2 including the initial hit, **P1** (MIC = 0.10 μM in GCas, 0.78 μM in GBSA), protein binding was detrimental to whole cell activity as evidenced by the 3–8-fold increase in MIC in protein-enriched (GBSA) media.

The carbonyl group between parts C and E was critical because replacement of the carbonyl with a sulfonyl group was not tolerated (Table 3: **P11**, MIC = 12.5 μM).

For the SAR study of the right-hand side part E, compounds **P12**–**P30** were synthesized to evaluate the benzyl-substituted piperidine moiety. Replacement of the piperidine with a piperazine (**P12** and **P13**) or with an aliphatic amine (**P14** and **P15**) abolished whole cell activity. Although **P16** without the benzyl group had complete loss of activity, simple aliphatic substituents like trifluoromethyl (**P17**), *tert*-butyl (**P18**), and isopropyl (**P19**) still showed moderate MICs in GCas medium with 2–8-fold higher MICs in GBSA media, suggesting a similar pattern on effect of protein binding as well as the importance of a hydrophilic substituent on the piperidine ring. Interestingly, **P20** with an embedded tetrahydroisoquinoline moiety as a fused bicyclic analogue had diminished activity, whereas its rotatable analogue, **P21**, with a phenyl conjugated piperidine, was modestly active against *Mtb* cells. The 20-fold loss of activity associated with replacement of the benzyl group of **P1** (MIC = 0.10 μM) with a phenyl group in **P21** (MIC = 2.3 μM) confirmed the importance of the conformational flexibility generated by the methyl linker between the piperidine and phenyl rings. In addition, the 4-benzylpiperidine moiety was crucial in terms of regioisomeric aspects as well as ring size because the regioisomer **P22** with a 3-benzylpiperidine moiety was inactive, and replacement of the piperidine with a smaller pyrrolidine, **P23**, reduced its activity about 60-fold (MIC = 6.25 μM). Replacement of the piperidine ring with a cyclohexyl amine (**P24**) resulted in an almost 50-fold reduction in activity (MIC = 4.7 μM) compared to **P1** with protein binding of **P24** in GBSA contributing to complete lack of whole cell activity. Analogs **P26**–**P29** were prepared to further examine the methyl linker between piperidine and phenyl ring, which demonstrated that conformation restriction due to unsaturation (**P26**) or a methyl substitution in the benzylic position (**P27**) did not impact whole cell activity (MIC = 0.30 and 0.20 μM, respectively) whereas the introduction of oxygen (**P28**, MIC = 2.3 μM) or replacement of the sp^3^ carbon with a carbonyl group (**P29**, MIC = 4.7 μM) was detrimental for activity, as evidenced by a 20–50-fold decrease in potency compared to **P1**.

Notably, replacement of the aromatic benzene with an aliphatic cyclohexane in **P25** improved the potency and reduced the detrimental effect of protein binding on whole cell activity (MIC = 0.05 μM in GCas and 0.20 μM in GBSA). Similarly, **P30**, which has a trifluoromethyl substituent in the *para* position on the phenyl ring, was equipotent to **P1** but its activity was only 3-fold lower in the presence of protein in the media (MIC = 0.10 μM in GCas and 0.30 μM in GBSA). Based on this preliminary SAR, further modification of part E with different heteroaryls, aliphatic cycles, and different substituents on the benzene ring would allow the optimization of compounds that retain potency in the presence of protein which is essential for *in vivo* proof of concept studies. Analysis of the correlation between MIC in either medium and cLogP for compounds **P1** and **P11**–**P30** based on the assumption that rings A, B, and C formed the key pharmacophore revealed no correlation between whole cell activity and hydrophobicity. Moreover, there was no correlation between cLogP and the effect of protein binding as measured by the fold difference between MIC values in protein-free and -supplemented media suggesting that BSA binding and access to the cellular target were not driven by hydrophobicity in contrast to reported correlates between cLogP and whole cell activity (Figure S1).^25^ This lack of correlation was unaffected by including all compounds with whole cell activity. The compounds had an excellent selectivity index as evidenced by the lack of cytotoxicity against HepG2 cells as well as lack of overt mitochondrial toxicity as seen by lack of cytotoxicity during growth of these cells on galactose as a carbon source (Table 2).^26^

The efficacy of **P1** against *Mtb* growing in host macrophages revealed that the compound exerted remarkable cidality at 5-fold the MIC value, even exceeding that of rifampicin (Figure 2). This suggested that the compound has access to the phagosomal environment of *Mtb* in the host and that the target pathway remained vulnerable during host pathogenesis. In addition, the level of pre-existing resistance in a panel of clinical isolates including several multidrug and extensively drug resistant strains was low with 5 strains exhibiting low (approximately 4–5-fold) levels of resistance (Table 4), suggesting that resistance mechanisms for clinically used drugs pose limited concern in potential drug development efforts for this scaffold.

To gain an understanding of the mechanism of action of **P1**, resistant mutants were generated on solid growth medium containing 5- and 10-fold the liquid MIC concentration of **P1**. Colonies grew up at frequencies of 3 × 10^–8^ cells at 5-fold MIC concentrations, whereas no resistant mutants could be generated at higher compound concentrations, even when plating 10^9^ cells. All colonies were found to be 4–16-fold resistant to **P1** as measured by liquid MIC determinations. Whole genome sequencing of 7 mutants revealed that 6 contained a T to C single nucleotide polymorphism (SNP) at position 4237005 of the *Mtb* H37Rv genome (re-sequenced) corresponding to a Leu368Pro mutation in DprE1, the decaprenylphosphoryl-β-d-ribose 2′-oxidase essential for cell wall arabinan biosynthesis of mycobacteria (Table 5). Leu368 is 13.5 Å away (Cβ to Cβ) from the Cys387 residue to which benzothiazinones covalently attach in the active site (such as BTZ043, see Figure 3). Leu368 is oriented away from the active site pocket toward the surface on the other side of the protein. However, Leu368 occurs at the terminus of a β-strand that is adjacent to the β-strand containing Cys387, and other residues adjacent to Leu368 (Lys367 and Val365) line the pocket and make contact with the inhibitor. We speculate that mutating Leu368 to proline at the end of this β-strand might induce a backbone shift that propagates to the adjacent residues in the strand, thereby prohibiting binding of **P1** (assuming it binds in the same pocket) while maintaining catalytic activity. Another mutant had an A to G SNP at position 4269441 resulting in a Ser173Pro mutation in UbiA, the decaprenylphosphoryl-5-phosphoribose synthase, which functions upstream of DprE1 (Table 5). This mutation has been previously identified in ethambutol resistant *Mtb* strains and likely affects flux through the arabinan biosynthetic pathway.^27^ Although several other SNPs were identified in the **P1**-resistant mutants, several have been found to convey low-level nonspecific resistance to a variety of inhibitors, including an insertion in *fadD26* encoding a fatty-acid-AMP ligase involved in PDIM biosynthesis likely affecting cell wall permeability and an insertion in *ppe13* which may affect porin-mediated uptake of the compound, which likely contribute to the range of resistance profiles (6–16-fold resistance) observed between the strains with *dprE1* polymorphisms (Table 5 and Table S1). In contrast, *ubiA* conferred lower levels of resistance, as previously observed with ethambutol.^27^ We confirmed that mutations in UbiA raised to other cell wall targeting inhibitors (unpublished data) also conferred low to moderate resistance whereas certain mutations that have been observed to confer resistance to other DprE1-targeting compounds including gave a diversity of resistance profiles with some mutations (Gly248Ser, Tyr314Cys, Pro116Ser) conferring resistance while strains with other mutations (Tyr314His, Asn364Ser) remained susceptible.^28−31^ This strongly suggests that **P1** likely targets the decaprenylphosphoryl-β-d-ribose 2′-oxidase, which is further corroborated by the positive response of our cell wall reporter assay.

**Figure 2:**
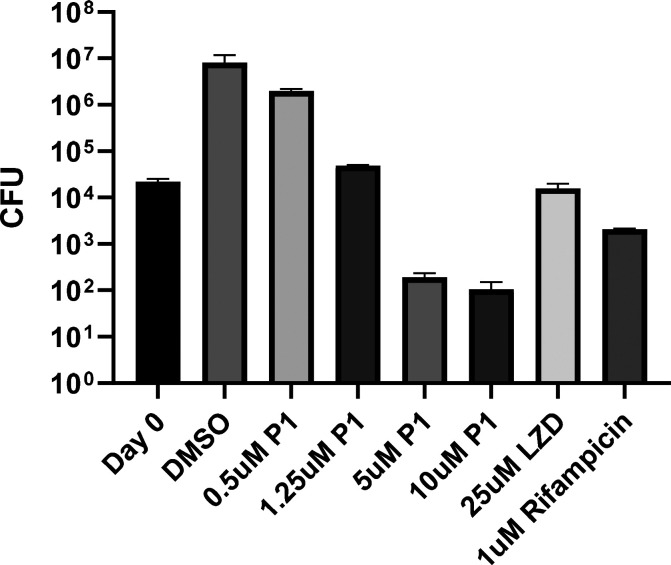
**P1** is cytotoxic against *Mtb* growing in macrophages. One day *Mtb*-infected J774 macrophages were treated for 7 days with the indicated compounds followed by colony forming unit enumeration on solid medium.

**Table 4:** Activity of **P1** against Drug-Susceptible and Drug-Resistant *Mtb* Strains

		MIC (μM)				MIC (μM)	
Strain	DST^a^	BDQ	P1	Strain	DST^a^	BDQ	P1
Drug-Susceptible Strains							
H37Rv ATCC27294		0.15	0.6	K11b00DS		0.3	1.56
Erdman		0.1	0.39	K12b00DS		0.04	1.56
HN878		0.05	0.78	K13b00DS		0.1	1.2
CDC1551		0.07	0.6	K14b00DS		0.6	1.2
CRC clinical strain K5072429		0.2	2.3	K15b00DS		0.07	0.78
K04b00DS		0.04	0.78	K16b00DS		0.1	0.78
K05b00DS		0.05	1.2	K17b00DS		0.07	0.78
K07b00DS		0.15	1.2	K35b00DS		0.07	1.2
K09b00DS		0.2	1.2	NIH_G12		0.05	0.78
K10b00DS		0.1	1.2				
Drug-Resistant Strains							
NIH_G11R	ECs	0.15	0.6	NIH1B21	HEth	0.07	1.56
NIH_G145	Eth	0.2	1.2	NIH1B314	HEThCsOThLZ	<0.02	3.13
NIH_G169	SCs	0.15	0.39	NIHA38	HTh	0.05	0.39
NIH_G17R	HEthCs	0.1	0.6	NIHB140	HTh	0.05	0.6
NIH1B127	HOTh	0.1	0.39	NIHB43	RHSTh	0.05	0.78
Multidrug-Resistant (MDR) Strains							
CRC clinical strain 01291696	HRS	0.05	0.78	NIH_G21R	HRERbCs	0.02	2.3
K18b01MR	HRERb	0.04	1.56	NIH_G22R	HRERb	0.1	1.2
K21b00MR	HRES	0.07	1.2	NIH_G269DR	HRERb	0.1	1.2
K22b00MR	HRERb	0.15	1.56	NIH_G79	HRSEth	0.07	1.56
K25b00MR	HREZRbTh	0.1	1.2	NIHA37	HRRb	0.05	0.39
K26b00MR	HREZRb	0.1	1.2	NIHA48	HREthTh	0.1	1.56
K29b00MR	HRSPO	0.2	1.2	NIHB302	HREZRbTh	0.04	0.78
NIH_G10R	HRERb	0.1	0.6	NIHB67	HRPOTh	0.3	0.78
NIH_G19R	HRESCsRb	0.1	1.56	NIHB68	HRPZThRb	0.1	0.39
MDR+ Strains							
K20b00MR	HREZSK	<0.02	4.7	NIH_G367DR	HRXM	<0.02	3.13
K32b00MR	HRKZCpmAmkThCs	0.1	1.56	NIH_G76MR	HRXM	<0.02	1.2
K33b00MR	HREZSKPTh	0.07	2.3	NIHB188	HRZThRbOML	0.04	0.6
Kb019	HREPKOTh	0.07	4.7	NIHB270	HREZThOLRb	0.07	1.56
Extensively Drug-Resistant (XDR) Strains							
026 K111	HRESKPCpmAmkThCsOM LRbZLzd	0.2	1.56	NIH1B178	HRSEKThCsPOL	0.05	0.78
028K111	HRESKPCpmAmkThCsOM LRbZLzd	0.15	1.56	NIH1B250	HRESPORbZThCsMLRbCpm	0.07	2.3
CRC clinical strain 00202293	HRESKPEthML	0.02	1.56	NIHB13	HRESKPThCsOMLAmkCpmTh	0.05	0.78
K37b00XR	HRKSXML	0.1	3.13				

a DST, drug resistance profile as determined by drug sensitivity testing. Amk, amikacin; Cpm, capreomycin; Cs, d-cycloserine; E, ethambutol; Eth, ethionamide; H, isoniazid; K, kanamycin; L, levofloxacin; Lzd, linezolid; M, moxifloxacin; O, ofloxacin; P, *para*-aminosalicylic acid; R, rifampicin; Rb, rifabutin; S, streptomycin; Th, prothionamide; Z, pyrazinamide.

**Table 5:** Mutations Observed in **P1**-Resistant Mutants

		**P1**-Resistant Strain (Fold Resistance)						
SNP position in chromosome (gene/protein)	H37Rv	A1 (6×)	A4 (8×)	B5 (8×)	B6 (8×)	C2 (8×)	C4 (16×)	D4 (4×)
4237005 (*Rv3790*/DprE1:L368P)	T	C	C	C	C	C	C	-
4269441 (*Rv3806c*/UbiA:S173P)	A	-	-	-	-	-	-	G
6684 (*Rv0005*/GyrB:R482L)	G	-	-	-	-	-	-	-
976904 (*Rv0878c*/PPE13:+1)	T	TG	TG	TG	TG	TG	TG	-
2181405 (*Rv1929c*/Rv1929c:T172S)	T	-	-	-	-	-	A	-
3244329 (*Rv2930*/FadD26:+1)	C	CA	CA	CA	CA	CA	CA	CA
3380454 (intergenic:+1)	G	-	GC	GC	GC	-	GC	GC
3537818 (*Rv3169*/Rv3169:A194P)	G	-	-	-	-	-	-	C
3558903 (3558902:T>A)	T	-	-	-	-	-	A	-
4124644 (*Rv3683*/Rv3683:V35A)	T	C	C	C	C	C	C	C

**Figure 3:**
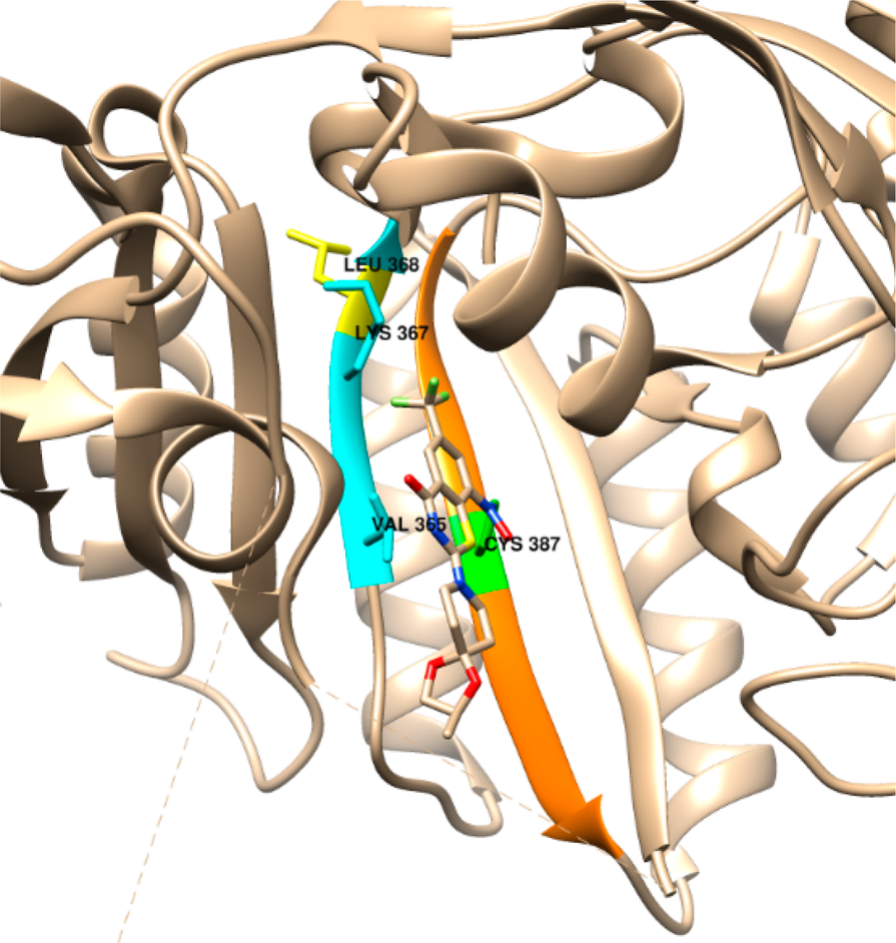
Proximity of L368 to C387 in the active site of DprE1 (PDB: 6HEZ). The benzothiazinone inhibitor BTZ043 is covalently attached to C387 (green). L368 (yellow) is oriented outside of the pocket, but the adjacent residues in the same β-strand (K367 and V365) line the active site pocket and contact the inhibitor. Mutating L368 to proline could cause a backbone shift affecting these neighboring residues in the β-strand (blue), which might abrogate binding of the oxadiazole inhibitor **P1**, providing a structural hypothesis for the mechanism of resistance.

In summary, screening of the 80,000 compound Janssen Jump-stARter Molecular Library yielded a phenyl oxadiazole piperidine scaffold with promising in vitro and intramacrophage activity against *Mtb*. Synthesis of a focused library of 29 analogs allowed us to identify the salient features on the scaffold that are essential for future drug development efforts on this hit. The identification of DprE1 as the likely cell wall biosynthetic target aligns with the known vulnerability of the arabinan biosynthetic pathway. There are several DprE1-targeting scaffolds currently under clinical evaluation for TB chemotherapy, and this phenyl oxadiazole piperidine adds a novel chemotype for future evaluation as a cell wall biosynthetic inhibitor in the TB drug development pipeline.

## Supporting Information Available

Supporting table and figure, experimental details for chemistry and biology, characterization data for synthetic compounds including the copies of NMR (^1^H and ^13^C) and mass (HRMS and UV) data (PDF)

## Abbreviations

*Mtb*: *Mycobacterium tuberculosis*
TB: tuberculosis
SAR: structure–activity relationship
MDR: multidrug-resistant
XDR: extensively drug-resistant
mAGP: mycolyl-arabinogalactan-peptidoglycan
InhA: NADH-dependent enoyl–acyl carrier protein reductase
MmpL3: mycobacterial membrane protein Large 3
DprE1: decaprenylphosphoryl-β-d-ribose 2′-oxidase
MIC: minimum inhibitory concentration
SNP: single nucleotide polymorphism
INH: isoniazid
BDQ: bedaquiline
DMSO: dimethylsulfoxide
DMF: dimethylformamide
EDC: 1-ethyl-3-(3-dimethylaminopropyl)carbodiimide hydrochloride
HOBt: *N*-hydroxybenzotriazole
DIPEA: *N,N*-diisopropylethylamine
DCM: dichloromethane
TFA: trifluoroacetic acid
