# Supplementary material for: Synthesis and Structure–Activity Relationships of a New Class of Oxadiazoles Targeting DprE1 as Antitubercular Agents
Source: ACS Med Chem Lett. 2023 Aug 15;14(9):1275–83. doi: 10.1021/acsmedchemlett.3c00295 (PMC10510505; doi:10.1021/acsmedchemlett.3c00295)

[Supporting Information]

Synthesis and structure-activity relationships of a new class of oxadiazoles targeting DprE1 as antitubercular agents

Veena D. Yadav[1,3], Helena I. Boshoff[1,3], Lena Trifonov[1], Jose Santinni O. Roma[1], Thomas R. Ioerger[2], Clifton E. Barry III[1], Sangmi Oh[1,*]

[1] Tuberculosis Research Section, Laboratory of Clinical Immunology and Microbiology, National Institute of Allergy and Infectious Diseases (NIAID), National Institutes of Health (NIH), Bethesda, MD 20892, USA

[2] Department of Computer Science and Engineering, Texas A&M University, College Station, Texas 77843, USA

[3] These authors contributed equally

* Corresponding author.

Tel.: +1-301-761-6401; fax: +1-301-480-3506; e-mail: sangmi.oh@nih.gov

Table of Contents

1. Supporting table and figure

Table S1 ………………………………………………………………………… S3

Figure S1 ………………………………………………………………………… S4

2. Experimental details

2-1. Chemistry ………………………………………………………………………… S5

2-2. Biology ………………………………………………………………………… S13

3. Copies of NMR and HRMS spectra ………………………………………………………… S14

1. Supporting Table and Figure

Table S1. Resistance levels different strains with defined mutations in DprE1 or MmpL3 to **P1**

Strains	MIC (μM)		
	P1	Ethambutol	Isoniazid
WT	0.6	1	0.2
EU8 5X1 (UbiA: G165V)	2.3	7.8	0.2
EU8 5x3 (UbiA: G165V)	3.1	7.8	0.2
KRT488 2x2 (UbiA: S176V)	1.6	3.9	0.2
KRT488 2X (UbiA: S173P)	1.2	6	0.2
KRT488 10x1 (DprE1: G248S)	1.6	1	0.2
1C1 (DprE1: Y314H)	0.2	1.5	0.2
1C2 (DprE1: Y314H)	0.2	1.5	0.2
1C10 (DprE1: Y314C)	2.3	1.5	0.2
1C16 (DprE1: N364S)	0.4	1	0.2
IC17 (DprE1: P116S)	2.3	1.5	0.2
C11-3 (DprE1: Y314H)	0.2	1	0.2
C11-6 (DprE1: L368P)	4.7	1	0.2
C16-2 (MmpL3: L166P)	0.3	1	0.2
C16-6 (MmpL3: L166P)	0.2	1	0.2
DA5-2 (MmpL3: A700T)	0.39	1.5	0.2
DA8-1 (MmpL3: L567P)	0.6	1.95	0.2
DA8-2 (MmpL3: Q40R and T2055375C)	0.39	1.5	0.2
TG22-1 (MmpL3: T284A)	0.3	1.5	0.2
TG22-4 (MmpL3: F644L)	0.3	1	0.2

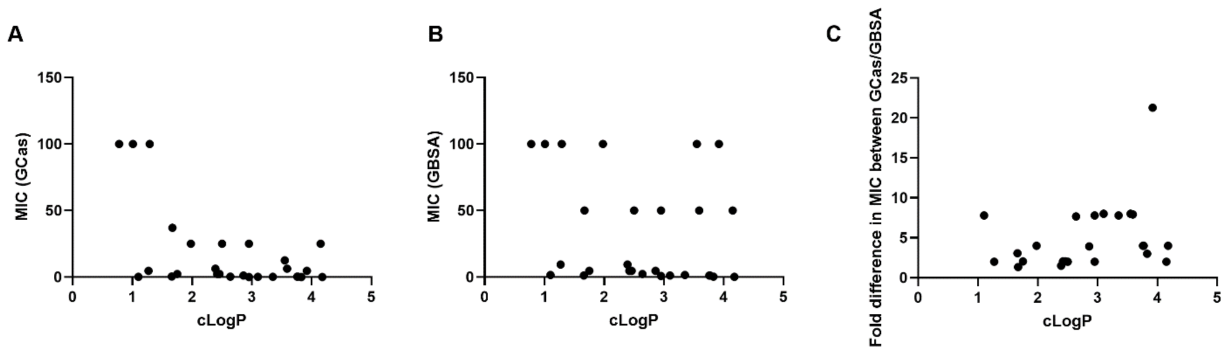

Figure S1. Lack of correlation between cLogP and whole-cell activity. Whole cell activity as determined in protein-free (GCas) (panel A) or protein replete (panel B) media.

2. Experimental details

No unexpected or unusually high safety hazards were encountered.

2-1. Chemistry

All reactions were monitored by analytical thin layer chromatography (TLC, silica gel 60 F254 0.25 mm) or routine mass and purity analyses (LRMS). ^{1}H NMR and ^{13}C NMR spectra were recorded on Bruker AVANCE III HD NanoBay 400 MHz spectrometers with $CDCl_3$ with tetramethylsilane as internal standard, as solvent and chemical shifts were measured in ppm relative to the specific solvent signal. Peak multiplicities of 1H-NMR signals were designated as s (singlet), bs (broad singlet), d (doublet), dd (doublet of doublet), t (triplet), m (multiplet) etc. Coupling constants (J) are in Hz. Routine mass and purity analyses (LRMS) were performed on an HP Agilent LC/MS series 1100 system equipped with a reverse phase column (Agilent Poroshell 120 EC-C18, 2.7 μm, 50 × 2.1 mm) and photodiode array detector coupled to an Agilent 1946 DSL quadrupole mass selective detector using electrospray ionization (ESI). The gradient mobile phase consisting of acetonitrile/water with 0.1% formic acid and UV detection at 254 nm was used to confirm all final products to be ≥95%. Accurate masses (HRMS) were obtained using a Waters LCT Premiere time-of-flight mass spectrometer. The instrument was operated in W-mode at a resolution of 10,000 in positive-ion mode. Ions were generated with Z-Spray electrospray ionization (ESI) with a capillary voltage of 3.4 kV. Accurate masses were determined using the internal-standard method. SiliaFlash P60 (40−60 μm) used in flash column chromatography was purchased from Silicycle Inc. Analytical grade solvents for the column chromatography were used as received. Commercial grade reagents and solvents were used without further purification. All reactions were assembled and were monitored by analytical thin layer chromatography (TLC). TLC was performed on pre-coated silica gel plates. After elution, plate was visualized under UV.

General synthesis of compound P1-P5, P8-P10 and P12- P30 can be performed as exemplified for P1

(4-Benzylpiperidin-1-yl)(1-(5-phenyl-1,3,4-oxadiazol-2-yl)piperidin-4-yl)methanone **(P1)**

Step1: To a solution of the 2-(methylsulfonyl)-5-phenyl-1,3,4-oxadiazole (**1,** 1.0 equiv) in anhydrous DMF, K_2CO_3 (2.0 equiv) and methyl piperidine-4-carboxylate (**2**, 1.2 equiv) were added. The reaction mixture was stirred overnight at rt. After completion of the reaction, water was added and then extracted with EtOAc (2×10 mL). The combined organic extracts were washed with water (2×5 mL) and brine. The combined organic layer was dried over Na_2SO_4 and concentrated *in vacuo.* The residue was purified by column chromatography to afford intermediate methyl 1-(5-phenyl-1,3,4-oxadiazol-2-*yl*)piperidine-4-carboxylate (**3**) in 64% yield as an off white solid: R*f* = 0.42 (MeOH/DCM = 5/95); ^{1}H NMR (400 MHz, $CDCl_3$) δ (ppm) 1.81-1.90 (m, 2H), 2.03-2.07 (m, 2H), 2.53-2.61 (m, 1H), 3.17-3.24 (m, 2H), 3.72 (s, 3H), 4.02-4.07 (m, 2H), 7.44-7.45 (m, 1H); ^{13}C NMR (100 MHz, $CDCl_3$) δ (ppm) 27.2, 40.4, 45.8, 52.0, 124.7, 125.9, 128.9, 130.6, 159.4, 164.2, 174.5.

Step 2: A mixture of methyl 1-(5-phenyl-1,3,4-oxadiazol-2-yl)piperidine-4-carboxylate (**3**) in 1N NaOH/MeOH (5/1) was stirred for 1.5 hr at 50 °C. After cooling down to rt, the mixture was concentrated *in vacuo*. The residue was suspended in EtOAC and 1N HCl and then the separated organic layer was evaporated to afford 1-(5-phenyl-1,3,4-oxadiazol-2-yl)piperidine-4-carboxylic acid. This can be used without further purification for EDC coupling to afford the corresponding product.

Step 3: To a solution of acid (1.0 equiv), EDC (1.0 equiv) and HOBt (0.5 equiv) in anhydrous DCM was added DIPEA (1.2 equiv) at 0 °C under Ar atmosphere. The recation mixture was stirred for 10 min. 4-Benzyl piperidine (1.1 equiv) was added and then the reaction mixture was stirred at rt for 8 hr. The reaction mixture was extracted with DCM and was dried over Na_2SO_4, concentrated *in vacuo* and purified by column chromatography on silica gel to afford the title compound **P1** as a white solid (78%): R*f* = 0.59 (MeOH/DCM = 1/9); ^{1}H NMR (400 MHz, $CDCl_3$) δ (ppm) 1.11-1.20 (m, 2H), 1.69-1.83 (m, 5H), 1.88-1.97 (m, 2H), 2.47-2.57 (m, 3H), 2.70-2.75 (m, 1H), 2.97-3.03 (m, 1H), 3.11-3.17 (m, 2H), 3.88 (d, *J* = 13 Hz, 1H), 4.12-4.15 (m, 2H), 4.60 (d, *J* = 13 Hz, 1H), 7.13 (d, *J* = 7 Hz, 2H), 7.18-7.22 (m, 1H), 7.29-7.30 (m, 2H), 7.42-7.44 (m, 3H), 7.89-7.92 (m, 2H); ^{13}C NMR (100 MHz, $CDCl_3$) δ (ppm) 27.6, 27.9, 31.9, 33.0, 37.7, 38.3, 42.3, 42.9, 45.8, 124.7, 125.8, 126.1, 128.4, 128.8, 129.1, 130.5, 139.9, 159.3, 164.2, 172.2; HRMS (ESI) calcd for $C_{26}H_{31}N_4O_2$ $[M + H]^+$ 431.2447, found 431.2453.

(4-Benzylpiperidin-1-yl)(1-(5-methyl-1,3,4-oxadiazol-2-yl)piperidin-4-yl)methanone **(P2).** A brown oil (73%): R*f* = 0.49 (MeOH/DCM = 1/9); ^{1}H NMR (400 MHz, $CDCl_3$) δ (ppm) 1.11-1.92 (m, 2H), 1.70-1.92 (m, 7H), 2.39 (s, 3H), 2.47-2.61 (m, 3H), 2.67-2.72 (m, 1H), 2.96-3.07 (m, 3H), 3.86-3.89 (m, 1H), 4.0-4.06 (m, 2H), 4.59-4.63 (d, *J* = 13.2 Hz, 1H), 7.13 (d, *J* = 7.1 Hz, 2H), 7.19-7.23 (m, 1H), 7.27-7.31 (m, 2H); ^{13}C NMR (100 MHz, $CDCl_3$) δ (ppm) 10.9, 27.3, 27.6, 31.6, 32.7, 37.5, 38.0, 42, 42.7, 45.6, 125.9, 128.1, 128.9, 139.6, 157.5, 164.2, 172.0; LRMS (ESI) m/z calcd for $C_{21}H_{29}N_4O_2$ $[M + H]^+$, 369.2; found 369.2.

(4-Benzylpiperidin-1-yl)(1-(5-(pyridin-4-yl)-1,3,4-oxadiazol-2-yl)piperidin-4-yl)methanone **(P3).** A white solid (76%): R*f* = 0.52 (MeOH/DCM = 1/9); ^{1}H NMR (400 MHz, $CDCl_3$) δ (ppm) 1.12 (m, 2H), 1.67-1.85 (m, 5H), 1.90-2.01 (m, 2H), 2.49-2.62 (m, 3H), 2.72-2.78 (m, 1H), 3.02 (t, *J* = 12.6 Hz, 1H), 3.16-3.22 (m, 2H), 3.89 (d, *J* = 6.6 Hz, 1H), 4.16-4.18 (m, 2H), 4.62 (d, *J* = 13.7 Hz, 1H), 7.14 (d, *J* = 7.4 Hz, 2H), 7.19-7.23 (m, 1H), 7.26-7.31 (m, 2H), 7.75 (d, *J* = 5.6 Hz, 2H), 8.72 (d, *J* = 4.0 Hz, 2H); ^{13}C NMR (100 MHz, $CDCl_3$) δ (ppm) 27.5, 27.8, 31.8, 32.9, 37.4, 38.2, 42.2, 42.9, 45.7, 119.2, 126.1, 128.3, 129.0, 131.6, 139.8, 150.5, 157.2, 164.5, 172.0; HRMS (ESI) calcd for $C_{25}H_{30}N_5O_2$ $[M + H]^+$ 432.2400, found 432.2406.

(4-Benzylpiperidin-1-yl)(1-(5-(4-fluorophenyl)-1,3,4-oxadiazol-2-yl)piperidin-4-yl)methanone **(P4).** A off white solid (78%): R*f* = 0.49 (MeOH/DCM = 1/9); ^{1}H NMR (400 MHz, $CDCl_3$) *δ* (ppm) 1.13-1.21 (m, 2H), 1.69-1.83 (m, 5H), 1.90-1.96 (m, 2H), 2.47-2.56 (m, 3H), 2.72-2.77 (m, 1H), 2.96-3.03 (m, 1H), 3.11-3.18 (m, 2H) 3.89 (d, *J* = 13 Hz, 1H), 4.09-4.14 (m, 2H), 4.60 (d, *J* = 12.9 Hz, 1H), 7.09-7.14 (m, 4H), 7.19 (t, *J* = 7.3 Hz, 1H), 7.28 (t, *J* = 7.4 Hz, 2H), 7.87-7.90 (m, 2H); ^{13}C NMR (100 MHz, $CDCl_3$) *δ* (ppm) 27.7 (d), 31.9, 33.0, 37.7, 38.4, 42.3, 43.0, 45.9, 116.0, 116.3, 121.1, 126.2, 127.9, 128.0, 128.4, 129.1, 139.9, 158.6, 162.8, 164.3, 165.3, 172.2; HRMS (ESI) calcd for $C_{26}H_{30}N_4O_2F$ $[M + H]^+$ 449.2353, found 449.2357.

(4-Benzylpiperidin-1-yl)(1-(5-(4-(trifluoromethyl)phenyl)-1,3,4-oxadiazol-2-yl)piperidin-4-yl)methanone **(P5).** A white solid (77%): R*f* = 0.51 (MeOH/DCM = 1/9); ^{1}H NMR (400 MHz, $CDCl_3$) *δ* (ppm) 1.09-1.18 (m, 2H), 1.67-1.95 (m, 7H), 2.45-2.58 (m, 3H), 2.69-2.76 (m, 1H), 2.95-3.01 (m, 1H), 3.12-3.19 (m, 2H), 3.86 (d, *J* = 13.1 Hz, 1H), 4.11-4.13 (m, 2H), 4.59 (d, *J* = 13.2 Hz, 1H), 7.10 (d, *J* = 7 Hz, 2H), 7.17 (m, 1H), 7.24-7.28 (m, 2H); 7.66 (d, *J* = 8.3 Hz, 2H), 7.98 (d, *J* = 8.2 Hz, 2H); ^{13}C NMR (100 MHz, $CDCl_3$) *δ* (ppm) 27.7(d), 31.9, 33.0, 37.6, 38.3, 42.3, 42.9, 45.8,

122.4, 125.2, 125.9, 126.2, 127.9, 128.4, 129.1, 131.5, 131.8, 132.1, 132.5, 139.8, 158.1, 164.4, 172.1; LRMS (ESI) m/z calcd for $C_{27}H_{30}F_3N_4O_2$[M + H]$^+$, 499.2; found 499.2.

Methyl 1-(5-phenyl-1,3,4-thiadiazol-2-yl)piperidine-4-carboxylate **(5a).** To a solution of 2-chloro-5-phenyl-1,3,4-thiadiazole (1.0 equiv) in 5 mL of anhydrous DMF, methyl piperidine-4-carboxylate (1.2 equiv) and K_2CO_3 (2.0 equiv) were added under Ar atmosphere. The reaction mixture was heated overnight at 110 ºC. After cooling down to rt, it was suspended in EA and water, and then organic layer was separated and dried under $MgSO_4$. The evaporated residue was purified by column chromatography to afford intermediate **5a** as a white solid (81%): R*f* = 0.51 (EtOAc/Hex = 2/3); ^{1}H NMR (400 MHz, $CDCl_3$) δ (ppm) 1.89-1.93 (m, 2H), 2.03-2.07 (m, 2H), 2.55-2.62 (m, 1H), 3.23-3.30 (m, 2H), 3.71 (s, 3H), 3.96-3.99 (m, 2H), 3.71 (s, 3H), 3.96-3.99 (m, 2H), 7.40-7.43 (m, 3H), 7.79-7.81 (m, 2H; ^{13}C NMR (100 MHz, $CDCl_3$) δ (ppm) 27.3, 40.5, 49.5, 52.0, 127.9, 129.0, 129.8, 131.2, 158.6, 171.7, 174.5; LRMS (ESI) m/z calcd for $C_{15}H_{18}N_3O_2S$ [M + H]$^+$, 499.2; found 499.2.

(4-Benzylpiperidin-1-yl)(1-(5-phenyl-1,3,4-thiadiazol-2-yl)piperidin-4-yl)methanone **(P6).** Basic hydrolysis of **5a** as followed step 2 and 3 of **P1** to afford **P6** as a white solid (79%): R*f* = 0.50 (MeOH/DCM = 1.5/8.5); ^{1}H NMR (400 MHz, $CDCl_3$) δ (ppm) 1.09-1.18 (m, 2H), 1.67-1.82 (m, 5H), 1.90-2.01 (m, 2H), 2.46-2.56 (m, 3H), 2.70-2.75 (m, 1H), 2.93-3.0 (m, 1H), 3.17-3.23 (m, 2H), 3.86 (d, J = 13.1 Hz, 1H), 4.04 (t, J = 13.4 Hz, 2H), 4.6 (d, J = 13.1 Hz, 1H), 7.12 (d, J = 7 Hz, 2H), 7.19 (t, J = 7.3 Hz, 1H), 7.26-7.30 (m, 2H), 7.34-7.41 (m, 3H), 7.78-7.80 (m, 2H); ^{13}C NMR (100 MHz, $CDCl_3$) δ (ppm) 27.6, 27.9, 31.8, 32.9, 37.7, 38.2, 42.1, 42.8, 45.7, 49.5, 126.0, 126.7, 128.3, 128.8, 129.0, 129.6, 131.1, 139.8, 158.2, 171.7, 172.0; HRMS (ESI) calcd for $C_{26}H_{31}N_4OS$ [M + H]$^+$ 447.2219, found 447.2227.

Methyl 1-(5-phenyl-1,2,4-oxadiazol-3-yl)piperidine-4-carboxylate **(5b).** To a solution of 4-bromo-2-phenyloxazole (1.0 equiv) in 5 mL of anhydrous DMF, methyl piperidine-4-carboxylate (1.2 equiv) and K_2CO_3 (2.0 equiv) were added under Ar atmosphere. The reaction mixture was heated overnight at 110 ºC. After cooling down to rt, it was suspended in EA and water, and then organic layer was separated and dried under $MgSO_4$. The evaporated residue was purified by column chromatography to afford intermediate **5b** as a white solid (42%): R*f* = 0.45 (EA/ Hex = 1/1); ^{1}H NMR (400 MHz, $CDCl_3$) δ (ppm) 1.77-1.87 (m, 2H), 1.99-2.03 (m, 2H), 2.51-2.58 (m, 1H), 3.03-3.10 (m, 2H), 3.70 (s, 3H), 4.03-4.08 (m, 2H), 7.46-7.50 (m, 2H), 7.53-7.57 (m, 1H), 8.05-8.07 (m, 2H; ^{13}C NMR (100 MHz, $CDCl_3$) δ (ppm) 27.3, 40.8, 45.6, 51.8, 124.8, 127.9, 128.9, 132.4, 170.7, 174.4, 174.9; LRMS (ESI) m/z calcd for $C_{15}H_{18}N_3O_3$ [M + H]$^+$, 288.1; found 288.1.

(4-Benzylpiperidin-1-yl)(1-(5-phenyl-1,2,4-oxadiazol-3-yl)piperidin-4-yl)methanone **(P7).** Basic hydrolysis of **5b** as followed step 2 and 3 of **P1** to afford **P7** as a white solid (81%): R*f* = 0.61 (MeOH/DCM = 1.5/8.5); ^{1}H NMR (400 MHz, $CDCl_3$) δ (ppm) 1.13-1.19 (m, 2H), 1.68-1.78 (m, 5H), 1.87-1.98 (m, 2H), 2.46-2.56 (m, 3H), 2.66-2.73 (m, 1H), 2.94-3.03 (m, 3H), 3.89 (d, J = 13.1 Hz, 1H), 4.15 (d, J = 11.2 Hz, 2H), 4.62 (d, J = 13.0 Hz, 1H), 7.13 (d, J = 7.12 Hz, 2H), 7.18-7.21 (m, 1H), 7.26-7.30 (m, 2H), 7.45-7.48 (m, 2H), 7.51-7.55 (m, 1H), 8.05 (d, J = 7.2 Hz, 2H); ^{13}C NMR (100 MHz, $CDCl_3$) δ (ppm) 27.7 (d), 31.7, 32.8, 38.1 (d), 42.0, 42.8, 45.7, 124.6, 125.9, 127.7, 128.2, 128.8, 129.0, 132.2, 139.8, 170.5, 172.4, 174.2; HRMS (ESI) calcd for $C_{26}H_{31}N_4O_2$ [M + H]$^+$ 431.2447, found 431.2452.

(4-Benzylpiperidin-1-yl)(1-(5-phenyl-1,3,4-oxadiazol-2-yl)piperidin-3-yl)methanone **(P8).** A light yellow solid (78%): R*f* = 0.59 (MeOH/DCM = 1/9); ^{1}H NMR (400 MHz, $CDCl_3$) δ (ppm) 1.09-1.29 (m, 2H), 1.64-1.96 (m, 7H), 2.48-2.62 (m, 3H), 2.48-2.62 (m, 3H), 2.83-2.88 (m, 1H), 2.95-3.13 (m, 2H), 3.25-3.48 (m, 1H), 3.95-4.01 (m, 1H), 4.09-4.16 (m, 2H), 4.60 (d, J = 13.1 Hz, 1H), 7.12-7.15 (m, 2H), 7.19-7.22 (m, 1H), 7.26-7.30 (m, 2H), 7.43-7.45 (m, 3H), 7.90-7.91 (m, 2H); ^{13}C NMR (100 MHz, $CDCl_3$) δ (ppm) 23.7, 27.0, 27.4, 31.5, 32.5, 32.6, 37.7, 37.9, 41.7 (d), 42.5, 45.4, 46.6, 48.7, 48.9, 124.3, 125.3, 125.8, 128.0, 128.5, 128.8, 130.2, 139.6, 158.9, 163.7, 170.5 (d); LRMS (ESI) m/z calcd for $C_{26}H_{31}N_4O_2$ $[M + H]^+$, 431.2; found 431.2.

(4-Benzylpiperidin-1-yl)(1-(5-phenyl-1,3,4-oxadiazol-2-yl)pyrrolidin-3-yl)methanone **(P9).** A white solid (74%): R*f* = 0.47 (MeOH/DCM = 1/9); ^{1}H NMR (400 MHz, $CDCl_3$) δ (ppm) 1.12-1.25 (m, 2H), 1.71-1.85 (m, 3H), 2.16-2.25 (m, 1H), 2.31-2.42 (m, 1H), 2.49-2.62 (m, 3H), 3.0-3.07 (m, 1H), 3.36-3.43 (m, 1H), 3.60-3.67 (m, 1H), 3.76-3.85 (m, 3H), 3.87-3.92 (m, 1H), 4.62 (d, J = 13.4 Hz, 1H), 7.11-7.15 (m, 2H), 7.19-7.23 (m, 1H), 7.28-7.31 (m, 2H), 7.42-7.45 (m, 3H), 7.89-7.96 (m, 2H); ^{13}C NMR (100 MHz, $CDCl_3$) δ (ppm) 29.0 (d), 31.6, 32.6, 38.6 (d), 40.3, 42.3, 42.7, 45.7, 47.3, 50.4 (d), 124.5, 125.5, 125.9, 128.2, 128.5, 128.6, 128.9, 130.1, 139.6, 158.9, 162.2, 169.7; LRMS (ESI) m/z calcd for $C_{25}H_{29}N_4O_2$ $[M + H]^+$, 417.2; found 417.2.

1-(4-Benzylpiperidin-1-yl)-2-(1-(5-phenyl-1,3,4-oxadiazol-2-yl)piperidin-4-yl)ethan-1-one **(P10).** A white oil (65%): R*f* = 0.45 (MeOH/DCM = 1/9); ^{1}H NMR (400 MHz, $CDCl_3$) δ (ppm) 1.08-1.20 (m, 2H), 1.29-1.40 (m, 2H), 1.68-1.79 (m, 3H), 1.87 (d, J = 12.5 Hz, 2H), 2.09-2.13 (m, 1H), 2.26 (d, J = 6.8 Hz, 2H), 2.46-2.59 (m, 3H), 2.90-2.97 (m, 1H), 3.10 (t, J = 12.5 Hz, 2H), 3.82 (d, J = 13.4 Hz, 1H), 4.09 (d, J = 12.3 Hz, 2H), 4.62 (d, J = 13.2 Hz, 1H), 7.12 (d, J = 7 Hz, 2H), 7.17-7.21 (m, 1H), 7.26-7.30 (m, 2H), 7.42-7.43 (m, 3H), 7.89-7.91 (m, 2H) ; ^{13}C NMR (100 MHz, $CDCl_3$) δ (ppm) 31.1, 31.6, 32.3, 32.4, 37.9, 39.1, 41.8, 42.7, 45.7, 46.3, 124.5, 125.4, 125.8, 128.0, 128.6, 130.1, 139.6, 158.8, 164.0, 169.2; LRMS (ESI) m/z calcd for $C_{27}H_{33}N_4O_2$ $[M + H]^+$, 445.2; found 445.2.

tert-Butyl 4-((4-benzylpiperidin-1-yl)sulfonyl)piperidine-1-carboxylate **(7).** To a solution of 4-benzyl piperidine (1.2 equiv) and NEt_3 (2.0 equiv) in anhydrouus DCM, *tert*-butyl 4-(chlorosulfonyl)piperidine-1-carboxylate (1.2 equiv) was slowly added. The reaction mixture was stirred at rt for 2 hr. After completion of reaction water was added to the reaction and organic layer was washed with 1N HCl, followed by washed with saturated $NaHCO_3$ solution. The organic layer was dried over Na_2SO_4. The column purification afforded an off-white solid (72%) of desired product: R*f* = 0.52 (EtOAc/Hex = 2/3); ^{1}H NMR (400 MHz, $CDCl_3$) δ (ppm) 1.25-1.33 (m, 2H), 1.46 (s, 9H), 1.61-1.76 (m, 5H), 2.0 (d, J = 12.4 Hz, 2H), 2.55 (d, J = 6.6 Hz, 2H), 2.69-2.84 (m, 4H), 2.97-3.03 (m, 1H), 3.78 (d, J = 12.6 Hz, 2H), 4.23 (s, 2H), 7.12 (d, J = 7.2 Hz, 2H), 7.20 (d, J = 7.2 Hz, 1H), 7.27-7.30 (m, 2H); ^{13}C NMR (100 MHz, $CDCl_3$) δ (ppm) 26.1, 28.5, 32.4, 37.8, 42.9, 46.7, 59.6, 80.1, 126.2, 128.4, 129.1, 139.8, 154.5; LRMS (ESI) m/z calcd for $C_{22}H_{35}N_2O_4S$ $[M + H]^+$, 423.2; found 423.2. Further boc protected **7** was dissolved in a 20 % (v/v) solution of TFA in DCM and the resulting solution was stirred at room temperature for 2 hr and concentrated. The resulting crude residue was dissolved in DCM, washed with 1 M solution of NaOH, dried over Na_2SO_4 and then concentrated. The crude produce was used for the next reaction without purification as following step 1 of the **P1** to afford **P11**

2-(4-((4-Benzylpiperidin-1-yl)sulfonyl)piperidin-1-yl)-5-phenyl-1,3,4-oxadiazole (P11). (P11). A white oil (52%): R*f* = 0.51 (MeOH/DCM = 1/9); ^{1}H NMR (400 MHz, $CDCl_3$) δ (ppm) 1.26-1.35 (m, 2H), 1.64-1.72 (m, 3H),

1.85-1.95 (m, 2H), 2.17 (d, J = 11.7 Hz, 2H), 2.56 (d, J = 6.7 Hz, 2H), 2.81-2.87 (m, 2H), 3.06-3.12 (m, 3H), 3.80 (d, J = 12.7 Hz, 2H), 4.23 (d, J = 13.2 Hz, 2H), 7.12 (d, J = 7.4 Hz, 2H), 7.20 (d, J = 7.4 Hz, 1H), 7.26-7.30 (m, 2H), 7.45-7.46 (m, 3H), 7.90-7.91 (m, 2H) ; ^{13}C NMR (100 MHz, $CDCl_3$) δ (ppm) 14.2, 25.5, 32.5, 37.8, 42.9, 45.5, 46.9, 53.5, 58.9, 124.5, 125.9, 126.3, 127.0, 127.9, 128.5, 128.7, 129.0, 129.2, 130.8, 139.8, 153.9, 159.9, 163.9; LRMS (ESI) m/z calcd for $C_{25}H_{31}N_4O_3S$ [M + H]$^+$, 467.2; found 467.2.

(4-Benzylpiperazin-1-yl)(1-(5-phenyl-1,3,4-oxadiazol-2-yl)piperidin-4-yl)methanone **(P12).** A white solid (71%): R*f* = 0.48 (MeOH/DCM = 1/9); ^{1}H NMR (400 MHz, $CDCl_3$) δ (ppm) 1.75 (s, 3H), 1.85-1.88 (m, 1H), 1.93-2.08 (m, 2H), 2.23-2.28 (m, 2H), 2.63-2.77 (m, 1H), 2.99-3.06 (m, 1H), 3.12-3.24 (m, 1H), 3.34-3.38 (m, 1H), 3.49-3.53 (m, 1H), 4.08-4.13 (m, 1H), 4.18-4.22 (m, 1H), 4.61 (d, J = 15.3 Hz, 2H), 4.69 (.d, J = 7.4 Hz, 1H), 4.76-4.83 (m, 1H), 7.17-7.22 (m, 2H), 7.28-7.34 (m, 2H), 7.37-7.41 (m, 1H), 7.43-7.46 (m, 3H), 7.88-7.93 (m, 2H). ^{13}C NMR (100 MHz, $CDCl_3$) δ (ppm) 22.5, 22.8, 28.0, 28.1, 35.8, 37.4, 38.2, 38.3, 44.9, 45.7, 45.8, 46.0, 51.2, 112.2, 113.0, 124.7, 125.9, 127.6, 128.0, 128.8, 130.6, 143.1, 159.6, 174.3; LRMS (ESI) m/z calcd for $C_{25}H_{31}N_5O_2$ [M + H]$^+$, 432.2; found 432.2.

(4-Methylpiperazin-1-yl)(1-(5-phenyl-1,3,4-oxadiazol-2-yl)piperidin-4-yl)methanone **(P13).** A light yellow solid (77%): R*f* = 0.45 (MeOH/DCM = 1/9); ^{1}H NMR (400 MHz, $CDCl_3$) δ (ppm) 1.82-1.86 (m, 2H), 1.91-2.01 (m, 2H), 2.33 (s, 3H), 2.38-2.45 (m, 4H), 2.71-2.76 (m, 1H), 3.12-3.19 (m, 2H), 3.56 (t, J = 4.6 Hz, 2H), 3.64-3.67 (m, 2H), 4.13-4.18 (m, 2H), 7.44-7.46 (m, 3H), 7.90-7.93 (m, 2H); ^{13}C NMR (100 MHz, $CDCl_3$) δ (ppm) 27.4, 37.1, 41.4, 45.1, 45.5, 45.7, 54.4, 55.1, 124.3, 125.4, 128.6, 130.2, 158.9, 163.9, 172.1; LRMS (ESI) m/z calcd for $C_{19}H_{26}N_5O_2$ [M + H]$^+$, 356.2; found 356.2.

N-Benzyl-1-(5-phenyl-1,3,4-oxadiazol-2-yl)piperidine-4-carboxamide **(P14).** A white solid (81%): R*f* = 0.53 (MeOH/DCM = 1/9); ^{1}H NMR (400 MHz, $CDCl_3$) δ (ppm) 1.85-2.01 (m, 4H), 2.34-2.42 (m, 1H), 3.08-3.14 (m, 2H), 4.11-4.16 (m, 2H), 4.45 (d, J = 5.6 Hz, 2H), 6.01 (s, 1H), 7.26-7.36 (m, 5H), 7.44-7.46 (m, 3H), 7.87-7.91 (m, 2H); ^{13}C NMR (100 MHz, $CDCl_3$) δ (ppm) 27.8, 42.3, 43.5, 45.9, 124.5, 125.7, 127.5, 127.6, 128.7, 128.9, 130.6, 138.4, 159.3, 164.1, 174.0; LRMS (ESI) m/z calcd for $C_{21}H_{23}N_4O_2$ [M + H]$^+$, 363.2; found 363.2.

N,N-Diethyl-1-(5-phenyl-1,3,4-oxadiazol-2-yl)piperidine-4-carboxamide **(P15).** Off white solid (77%): R*f* = 0.57 (MeOH/DCM = 1/9); ^{1}H NMR (400 MHz, $CDCl_3$) δ (ppm) 1.09-1.13 (m, 3H), 1.21-1.24 (m, 3H), 1.80 (d, J = 12.6 Hz, 2H), 1.93-2.03 (m, 2H), 2.65-2.71 (m, 1H), 3.13-3.18 (m, 2H), 3.34-3.40 (m, 4H), 4.15 (d, J = 13 Hz, 2H), 7.43-7.44 (m, 3H), 7.89-7.91 (m, 2H); ^{13}C NMR (100 MHz, $CDCl_3$) δ (ppm) 12.9, 14.9, 27.7, 37.7, 40.1, 41.7, 45.6, 124.5, 125.5, 128.6, 130.2, 159.0, 164.0, 173.1; LRMS (ESI) m/z calcd for $C_{18}H_{25}N_4O_2$ [M + H]$^+$, 329.2; found 329.2.

(1-(5-Phenyl-1,3,4-oxadiazol-2-yl)piperidin-4-yl)(piperidin-1-yl)methanone **(P16).** A white solid (75%): R*f* = 0.44 (MeOH/DCM = 1/9); ^{1}H NMR (400 MHz, $CDCl_3$) δ (ppm) 1.53-1.65 (m, 6H), 1.81-1.84 (m, 2H), 1.88-1.98 (m, 2H), 2.75-2.80 (m, 1H), 3.13-3.20 (m, 2H), 3.46-3.55 (m, 4H), 4.11-4.15 (m, 2H), 7.42-7.43 (m, 3H), 7.88-7.90 (m, 2H); ^{13}C NMR (100 MHz, $CDCl_3$) δ (ppm) 24.3, 25.3, 26.6, 27.4, 37.2, 42.6, 45.5, 46.2, 124.3, 125.4, 128.5, 130.1, 158.8, 163.9, 171.9; LRMS (ESI) m/z calcd for $C_{19}H_{25}N_4O_2$ [M + H]$^+$, 341.2; found 341.2.

(1-(5-Phenyl-1,3,4-oxadiazol-2-yl)piperidin-4-yl)(4-(trifluoromethyl)piperidin-1-yl)methanone **(P17).** A white solid (69%): R*f* = 0.45 (MeOH/DCM = 1/9); ^{1}H NMR (400 MHz, $CDCl_3$) δ (ppm) 1.36-1.42 (m, 2H), 1.72-1.91 (m, 6H),

2.18-2.26 (m, 1H), 2.45 (t, J = 12.2 Hz, 1H), 2.66-2.72 (m, 1H), 2.97-3.11 (m, 3H), 3.94-4.06 (m, 3H), 4.65 (d, J = 12.1 Hz, 1H); ^{13}C NMR (100 MHz, $CDCl_3$) δ (ppm) 24.3, 25.5, 27.6, 27.8, 37.7, 40.8, 41.0, 44.4, 45.8, 124.6, 125.8, 128.3, 128.9, 130.5, 159.4, 164.2, 172.4; LRMS (ESI) m/z calcd for $C_{20}H_{24}F_3N_4O_2$ $[M + H]^+$, 409.2; found 409.2.

(4-(tert-Butyl)piperidin-1-yl)(1-(5-phenyl-1,3,4-oxadiazol-2-yl)piperidin-4-yl)methanone **(P18).** A white solid (73%): R*f* = 0.47 (MeOH/DCM = 1/9); ^{1}H NMR (400 MHz, $CDCl_3$) δ (ppm) 0.87 (s, 9H), 1.10-1.29 (m, 3H), 1.73-2.08 (m, 6H), 2.43-2.49 (m, 1H), 2.71-2.79 (m, 1H), 2.97-3.03 (m, 1H), 3.12-3.19 (m, 2H), 3.98 (d, J = 13.3 Hz, 1H), 4.14-4.17 (m, 2H), 4.72 (d, J = 13.2 Hz, 1H), 7.44-7.45 (m, 3H), 7.90-7.92 (m, 2H); ^{13}C NMR (100 MHz, $CDCl_3$) δ (ppm) 26.8, 27.4, 27.7, 28.0, 28.1, 32.3, 37.8, 42.9, 46.0, 46.5, 47.0, 124.8, 125.9, 128.9, 130.5, 159.4, 164.3, 172.1; LRMS (ESI) m/z calcd for $C_{230}H_{33}N_4O_2$ $[M + H]^+$, 397.2; found 397.2.

(4-Isopropylpiperidin-1-yl)(1-(5-phenyl-1,3,4-oxadiazol-2-yl)piperidin-4-yl)methanone **(P19).** A white solid (75%): R*f* = 0.49 (MeOH/DCM = 1/9); ^{1}H NMR (400 MHz, $CDCl_3$) δ (ppm) 0.86 (d, J = 6.8 Hz, 6H), 1.06-1.18 (m, 2H), 1.23-1.30 (m, 1H), 1.40-1.48 (m, 1H), 1.68-1.99 (m, 6H), 2.44-2.50 (m, 1H), 2.76-2.82 (m, 1H), 2.97-3.03 (m, 1H), 3.17 (t, J = 12.5 Hz, 2H), 3.95 (d, J = 12.9 Hz, 2H), 4.13 (d, J = 11.0 Hz, 2H), 4.66 (d, J = 12.9 Hz, 1H), 7.41-7.43 (m, 3H), 7.88-7.90 (m, 1H); ^{13}C NMR (100 MHz, $CDCl_3$) δ (ppm) 18.4, 26.3, 26.5, 27.7, 28.9, 31.0, 36.2, 41.2, 41.4, 44.5, 44.7, 123.3, 124.4, 127.5, 129.1, 157.8, 162.9, 170.7; LRMS (ESI) m/z calcd for $C_{23}H_{31}N_4O_2$ $[M + H]^+$, 383.2; found 383.2.

(3,4-Dihydroisoquinolin-2(1H)-yl)(1-(5-phenyl-1,3,4-oxadiazol-2-yl)piperidin-4-yl)methanone **(P20).** A white solid (69%): R*f* = 0.43 (MeOH/DCM = 1/9); ^{1}H NMR (400 MHz, $CDCl_3$) δ (ppm) 1.86-2.02 (m, 4H), 2.80-2.88 (m, 2H), 2.95 (t, J = 5.7 Hz, 1H), 3.16-3.22 (m, 2H), 3.75-3.78 (m, 1H), 3.84-3.87 (m, 1H), 4.16-4.19 (m, 2H), 4.73 (d, J = 15.7 Hz, 2H), 7.15-7.24 (m, 4H), 7.44-7.45 (m, 3H), 7.90-7.92 (m, 2H); ^{13}C NMR (100 MHz, $CDCl_3$) δ (ppm) 27.5, 27.6, 28.4, 29.8, 38.1 (d), 40.1, 43.1, 44.5, 45.7, 47.4, 124.6, 125.7, 125.9, 126.4, 126.6, 127.1, 128.3, 128.8, 129.0, 130.4, 132.4, 133.3, 133.8, 135.0, 159.2, 164.1, 172.7 (d); LRMS (ESI) m/z calcd for $C_{23}H_{25}N_4O_2$ $[M + H]^+$, 389.2; found 389.2.

(1-(5-Phenyl-1,3,4-oxadiazol-2-yl)piperidin-4-yl)(4-phenylpiperidin-1-yl)methanone **(P21).** A white solid (76%): R*f* = 0.42 (MeOH/DCM = 1/9); ^{1}H NMR (400 MHz, $CDCl_3$) δ (ppm) 1.60-1.63 (m, 2H), 1.87-2.01 (m, 6H), 2.60-2.66 (m, 1H), 2.72-2.84 (m, 2H), 3.17 (t, J = 12.0 Hz, 3H), 4.03-4.16 (m, 3H), 4.79 (d, J = 12.8 Hz, 1H), 7.18-7.22 (m,3H), 7.28-7.32 (m, 2H), 7.40-7.42 (m, 3H), 7.88-7.91 (m, 2H); ^{13}C NMR (100 MHz, $CDCl_3$) δ (ppm) 27.4, 27.6, 32.6, 34.0, 37.4, 42.3, 42.4, 45.6, 45.9, 124.4, 125.5, 126.3, 126.5, 128.4, 128.6, 130.2, 144.8, 160.0, 163.9, 172.0; LRMS (ESI) m/z calcd for $C_{25}H_{29}N_4O_2$ $[M + H]^+$, 417.2; found 417.2.

(3-Benzylpiperidin-1-yl)(1-(5-phenyl-1,3,4-oxadiazol-2-yl)piperidin-4-yl)methanone **(P22).** A white solid (78%): R*f* = 0.6 (MeOH/DCM = 1/9); ^{1}H NMR (400 MHz, $CDCl_3$) δ (ppm) 1.17-1.48 (m, 3H), 1.67-1.95 (m, 6H), 2.29-2.45 (m, 2H), 2.58-2.75 (m, 3H), 2.87-3.18 (m, 2H), 3.60-3.84 (m, 1H), 3.95-4.16 (m, 2H), 4.45-4.54 (m, 1H), 7.13-7.35 (m, 5H), 7.44-7.45 (m, 3H), 7.90-7.92 (m, 2H; ^{13}C NMR (100 MHz, $CDCl_3$) δ (ppm) 24.9, 26.0, 27.3, 27.6, 30.4, 31.5, 37.6, 37.8, 39.7, 40.0, 40.2, 42.6, 45.6, 46.2, 47.8, 50.6, 124.5, 125.6, 126.0, 126.4, 128.2, 128.5, 128.7, 129.0, 130.3, 139.4, 159.2, 164.0, 171.9, 172.1; LRMS (ESI) m/z calcd for $C_{26}H_{31}N_4O_2$ $[M + H]^+$, 431.2; found 431.2.

(3-Benzylpyrrolidin-1-yl)(1-(5-phenyl-1,3,4-oxadiazol-2-yl)piperidin-4-yl)methanone **(P23).** A yellow oil (75%): R*f* = 0.59 (MeOH/DCM = 1/9); ^{1}H NMR (400 MHz, $CDCl_3$) *δ* (ppm) 1.59-1.99 (m, 6H), 2.39-2.60 (m, 2H), 2.64-2.78 (m, 2H), 3.07-3.19 (m, 3H), 3.34-3.50 (m, 1H), 3.58-3.71 (m, 2H), 4.14-4.18 (m, 2H), 7.15-7.34 (m, 5H), 7.43-7.45 (m, 3H), 7.89-7.92 (m, 2H); ^{13}C NMR (100 MHz, $CDCl_3$) *δ* (ppm) 25.9, 28.7, 30.5, 37.8, 37.9 (d), 38.3, 38.4, 39.9, 44.3, 44.5, 44.9, 49.9, 50.3, 123.4, 124.5, 125.0, 125.2, 127.3, 127.4, 127.5, 127.6, 129.2, 138.6, 138.7, 157.9, 162.9, 171.3, 171.4; LRMS (ESI) m/z calcd for $C_{25}H_{29}N_4O_2$ $[M + H]^+$, 417.2; found 417.2.

N-(4-Benzylcyclohexyl)-1-(5-phenyl-1,3,4-oxadiazol-2-yl)piperidine-4-carboxamide **(P24).** A white solid (79%): R*f* = 0.62 (MeOH/DCM = 1/9); ^{1}H NMR (400 MHz, $CDCl_3$) *δ* (ppm) 1.00-1.16 (m, 4H), 1.44-1.49 (m, 1H), 1.73-1.97 (m, 8H), 2.23-2.30 (m, 1H), 2.48-2.57 (m, 2H), 3.69-3.75 (m, 2H), 4.12-4.15 (m, 2H), 5.34 (d, *J* = 8.0 Hz, 1H), 7.12-7.14 (m, 2H), 7.16-7.20 (m, 1H), 7.25-7.29 (m, 1H), 7.43-7.46 (m, 3H); ^{13}C NMR (100 MHz, $CDCl_3$) *δ* (ppm) 27.9, 29.3, 31.6, 33.1, 39.0, 42.6, 43.5, 45.9, 48.6, 124.6, 125.8, 128.2, 128.9, 129.1, 130.6, 140.9, 159.3, 164.2, 173.1; LRMS (ESI) m/z calcd for $C_{27}H_{33}N_4O_2$ $[M + H]^+$, 445.2; found 445.2.

(4-(Cyclohexylmethyl)piperidin-1-yl)(1-(5-phenyl-1,3,4-oxadiazol-2-yl)piperidin-4-yl)methanone **(P25).** A white solid (77%): R*f* = 0.59 (MeOH/DCM = 1/9); ^{1}H NMR (400 MHz, $CDCl_3$) *δ* (ppm) 0.79-0.87 (m, 2H), 0.96-1.32 (m, 8H), 1.56-1.81 (m, 10H), 1.89-1.95 (m, 2H), 2.49-2.55 (m, 1H), 2.68-2.75 (m, 1H), 2.98-3.04 (m, 1H), 3.09-3.16 (m, 2H), 3.86 (d, *J* = 13.1 Hz, 1H), 4.12 (d, *J* = 12.6 Hz, 2H), 4.57 (d, *J* = 13.1 Hz, 1H), 7.40-7.42 (m, 3H), 7.87-7.89 (m, 2H); ^{13}C NMR (100 MHz, $CDCl_3$) *δ* (ppm) 26.4, 26.7, 27.6, 27.9, 32.3, 33.0, 33.7, 34.2, 37.7, 42.4, 44.3, 45.9, 124.7, 125.8, 128.8, 130.4, 159.3, 164.2, 172.1; HRMS (ESI) calcd for $C_{26}H_{37}N_4O_2$ $[M + H]^+$ 437.2917, found 437.2921.

(4-Benzylidenepiperidin-1-yl)(1-(5-phenyl-1,3,4-oxadiazol-2-yl)piperidin-4-yl)methanone **(P26).** An off-white solid (74%): R*f* = 0.59 (MeOH/DCM = 1/9); ^{1}H NMR (400 MHz, $CDCl_3$) *δ* (ppm) 1.84-2.03 (m, 4H), 2.38-2.56 (m, 4H), 2.74-2.80 (m, 1H), 3.12-3.20 (m, 2H), 3.48-3.72 (m, 4H), 4.16 (d, *J* = 10.3 Hz, 2H), 6.42 (s, 1H), 7.19-7.27 (m, 3H), 7.33 (t, *J* = 7.3 Hz, 2H), 7.43-7.45 (m, 3H), 7.91 (d, *J* = 3.4 Hz, 2H); ^{13}C NMR (100 MHz, $CDCl_3$) *δ* (ppm) 27.9, 29.1, 30.3, 35.9, 37.1, 37.9, 42.9, 43.8, 45.9, 46.2, 47.0, 124.8, 125.5, 125.9, 126.7, 128.4, 129.0, 130.6, 137.2, 159.4, 164.3, 172.5; LRMS (ESI) m/z calcd for $C_{26}H_{29}N_4O_2$ $[M + H]^+$, 429.2; found 429.2.

(1-(5-Phenyl-1,3,4-oxadiazol-2-yl)piperidin-4-yl)(4-(1-phenylethyl)piperidin-1-yl)methanone **(P27).** A white solid (76%): R*f* = 0.61 (MeOH/DCM = 1/9); ^{1}H NMR (400 MHz, $CDCl_3$) *δ* (ppm) 0.93-1.03 (m, 1H), 1.10 (m, 1H), 1.23 (d, *J* = 7 Hz, 3H), 1.38-1.45 (m, 1H), 1.57-1.65 (m, 1H), 1.74-1.79 (m, 1H), 1.86-1.91 (m, 3H), 2.34-2.52 (m, 2H), 2.63-2.74 (m, 1H), 2.85-2.99 (m, 1H), 3.04-3.11 (m, 2H), 4.50-4.67 (m, 1H), 7.10 (d, *J* = 6.8 Hz, 2H), 7.14-7.18 (m, 1H), 7.24-7.28 (m, 2H), 7.39-7.40 (m, 3H), 7.86 (d, *J* = 2.8 Hz, 2H); ^{13}C NMR (100 MHz, $CDCl_3$) *δ* (ppm) 18.7 (d), 27.6 (d), 29.8, 30.3, 31.7, 37.5, 42.2, 42.7, 45.1, 45.7, 124.6, 125.7, 126.1, 127.5, 128.3, 128.8, 130.4, 145.6 (d), 159.1, 164.1, 172; LRMS (ESI) m/z calcd for $C_{27}H_{33}N_4O_2$ $[M + H]^+$, 445.2; found 445.2.

(4-Phenoxypiperidin-1-yl)(1-(5-phenyl-1,3,4-oxadiazol-2-yl)piperidin-4-yl)methanone **(P28).** A white solid (79%): R*f* = 0.49 (MeOH/DCM = 1/9); ^{1}H NMR (400 MHz, $CDCl_3$) *δ* (ppm) 1.83-2.02 (m, 8H), 2.73-2.81 (m, 2H), 3.47-3.52 (m, 1H), 3.69-3.80 (m, 3H), 4.16 (d, *J* = 10.8 Hz, 2H), 4.56-4.61 (m, 1H), 6.92-6.99 (m, 3H), 7.26-7.32 (m, 2H), 7.45 (t, *J* = 3.3 Hz, 3H), 7.91-7.93 (m, 2H); ^{13}C NMR (100 MHz, $CDCl_3$) *δ* (ppm) 27.7, 30.2, 31.5, 37.6, 38.5, 42.2, 45.8,

71.4, 116.2, 121.3, 124.7, 125.8, 128.9, 129.7, 130.5, 157.0, 159.3, 164.2, 172.3; LRMS (ESI) m/z calcd for $C_{25}H_{29}N_4O_3$ [M + H]$^+$, 433.2; found 433.2.

(4-Benzoylpiperidin-1-yl)(1-(5-phenyl-1,3,4-oxadiazol-2-yl)piperidin-4-yl)methanone **(P29).** A white solid (80%): R*f* = 0.53 (MeOH/DCM = 1/9); ^{1}H NMR (400 MHz, $CDCl_3$) δ (ppm) 1.63-1.69 (m, 1H), 1.83-1.97 (m, 7H), 2.77-2.90 (m, 2H), 3.14-3.20 (m, 2H), 3.26-3.32 (m, 1H), 3.54-3.59 (m, 1H), 4.03 (d, J = 13.1 Hz, 1H), 4.14 (d, J = 13.0 Hz, 2H), 4.57-4.60 (m, 1H), 7.42-7.49 (m, 5H), 7.57 (t, J = 7.3 Hz, 1H), 7.89-7.91 (m, 2H), 7.94 (d, J = 7.5 Hz, 2H); ^{13}C NMR (100 MHz, $CDCl_3$) δ (ppm) 27.5 (d), 28.6 (d), 37.5, 41.3, 43.0, 44.8, 45.6, 124.5, 125.6, 128.1, 128.7, 130.3, 133.2, 135.5, 159.1, 164.0, 172.2, 201.5; LRMS (ESI) m/z calcd for $C_{26}H_{29}N_4O_3$ [M + H]$^+$, 445.2; found 445.2.

(1-(5-Phenyl-1,3,4-oxadiazol-2-yl)piperidin-4-yl)(4-(4-(trifluoromethyl)benzyl)piperidin-1-yl)methanone **(P30).** A white solid (77%): R*f* = 0.59 (MeOH/DCM = 1/9); ^{1}H NMR (400 MHz, $CDCl_3$) δ (ppm) 1.11-1.17 (m, 2H), 1.65-1.94 (m, 7H), 2.44-2.74 (m, 4H), 2.98 (t, J = 12.4 Hz, 1H), 3.08-3.15 (m, 2H), 3.88 (d, J = 13.1 Hz, 1H), 4.08-4.13 (m, 2H), 4.66 (d, J = 13.0 Hz, 1H), 7.22 (d, J = 7.9 Hz, 2H), 7.40 (t, J = 3.4 Hz, 3H), 7.51 (d, J = 8.0 Hz, 2H), 7.86-7.88 (m, 2H); ^{13}C NMR (100 MHz, $CDCl_3$) δ (ppm) 27.7 (d), 31.7, 32.8, 37.6, 38.1, 42.1, 42.6, 45.8, 124.6, 125.2, 125.7, 128.8, 129.3, 130.4, 144.0, 159.2, 164.1, 172.2; HRMS (ESI) calcd for $C_{27}H_{30}N_4O_2F_3$ [M + H]$^+$ 499.2321, found 499.2314.

2-2. Biology

Efficacy and validating inhibition of compounds against Mtb in vitro and ex vivo. The antimicrobial susceptibility testing against *Mtb* H37Rv (ATCC 27294) and the **P1**-resistant strains was performed in either GBSA (4.7 g/L Middlebrook 7H9 base, 5 g/L albumin fraction V, 4 g/L glucose, 0.81 g/L NaCl, and 0.05% Tyloxapol) or GCas (4.7 g/L Middlebrook 7H9 base, 4 g/L glucose, 0.81 g/L NaCl, 0.3 g/L Casitone, and 0.05% Tyloxapol). Clinical strains were tested in 7H9/ADC/Tw (4.7 g/L Middlebrook 7H9 base, 5 g/L albumin fraction V, 2 g/L glucose, 0.81 g/L NaCl, 0.02% glycerol, and 0.05% Tween 80). Bacteria were grown in the corresponding media up to an OD of 0.2–0.4 and diluted 1000-fold in the respective growth medium. An equal volume (50 μL) of diluted cells was added to a two-fold serial dilution series of the test compounds in the same medium in a sterile 96-well round bottom plate. Plates were incubated at 37°C for 2 weeks prior to assessing growth using an enlarging inverted mirror. The MIC is defined here as the drug concentration that completely inhibits growth of cells.

For assessment of intramacrophage activity, J774 cells (4×10^4 cells/well) were seeded in flat-bottom 24 well plates (Corning Inc.) in macrophage growth medium consisting of DMEM GlutaMAX (Gibco/ThermoFisher Scientific) supplemented with 10% fetal bovine serum, 20 mM HEPES + 0.5 mM sodium pyruvate (hereafter abbreviated DMEM/FBS). Cells were infected with *Mtb* at a multiplicity of infection of 1:1 for 24 hours, followed by medium removal and washing (2×) with an equal volume of DMEM. Infected cells were fed the macrophage growth medium supplemented with compounds or DMSO at the indicated concentration. Cells were incubated at 37°C, 95% humidity, 5% CO_2 incubator with drug-containing or vehicle control medium supplemented every 2 days. After 7 days incubation (8 days after infection), the medium was removed and replaced with 7H9/ADC/Tw containing 0.1% SDS. After 5 min, the lysate was thoroughly mixed and diluted in 7H9/ADC/Tw and appropriate dilutions plated in duplicate on Middlebrook 7H11/OAD (per liter: 20.5g Middlebrook 7H11 medium - Sigma Aldrich/ 4mL glycerol/ 2g glucose/ 0.85g NaCl/ 5g BSA fraction V/ 0.06 mL oleic acid) agar plates to determine bacterial counts after 6–8 weeks of incubation at 37°C.

Generation of resistant mutants. *Mtb* H37Rv (ATCC 27294) was grown to OD_{650nm} of 0.4 in 7H9/ADC/Tw and harvested by centrifugation. Cells were diluted in 7H9/ADC/Tw to 1×10^{10} cells/mL, 1×10^9 cells/mL and 1×10^8 cells/mL and 0.1mL volumes plated on Middlebrook 7H11/OAD agar medium containing **P1** at 5-fold liquid MIC concentrations. Appropriate dilutions of cells were plated on drug-free agar to enumerate number of cells plated on the drug-containing plates. After 4 weeks' incubation at 37°C, colonies were counted and picked from **P1**-containing plates into 7H9/ADC/Tw liquid medium for outgrowth. MIC testing of resistant mutants was performed as described above.

Cytotoxicity testing against Mammalian cells. The *in vitro* cytotoxicity of the compounds was measured against HepG2 cells grown in DMEM supplemented with 10% FBS using either glucose (5 mM) or galactose (10 mM) as carbon source. Briefly, 1×10^4 cells (HepG2) were seeded onto each well of a sterile 96-well tissue culture plate. The next day, media was aspirated and replaced with a two-fold serial dilution of compounds in the same media. Viability was determined after 24 using CellTiter-Glo (Promega) according to manufacturer's instructions.

3. Copies of NMR and HRMS spectra

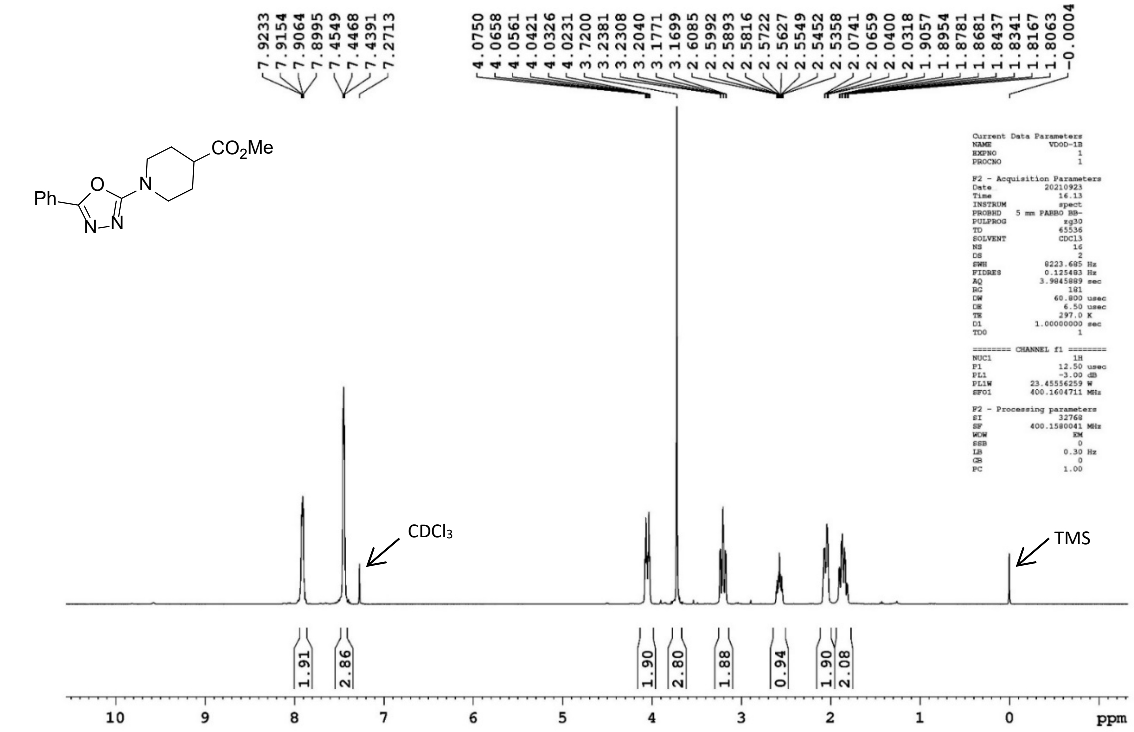

^{1}H NMR spectrum of methyl 1-(5-phenyl-1,3,4-oxadiazol-2-*yl*)piperidine-4-carboxylate (**3**)

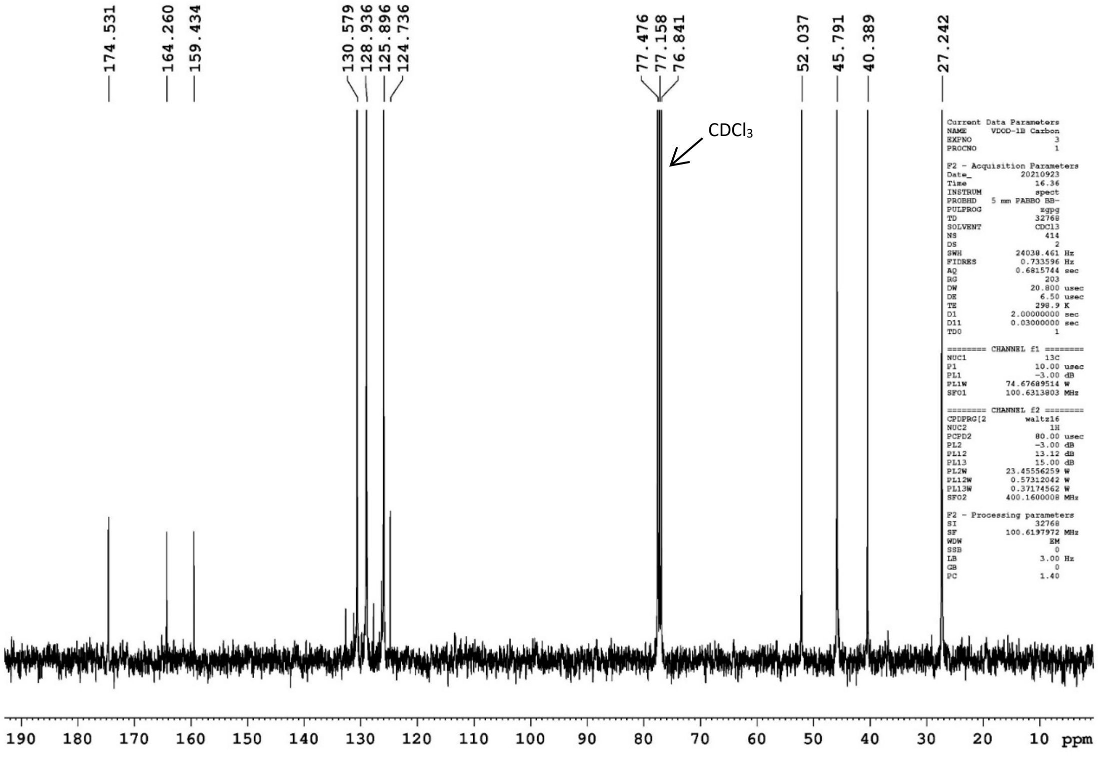

^{13}C NMR spectrum of methyl 1-(5-phenyl-1,3,4-oxadiazol-2-*yl*)piperidine-4-carboxylate (**3**)

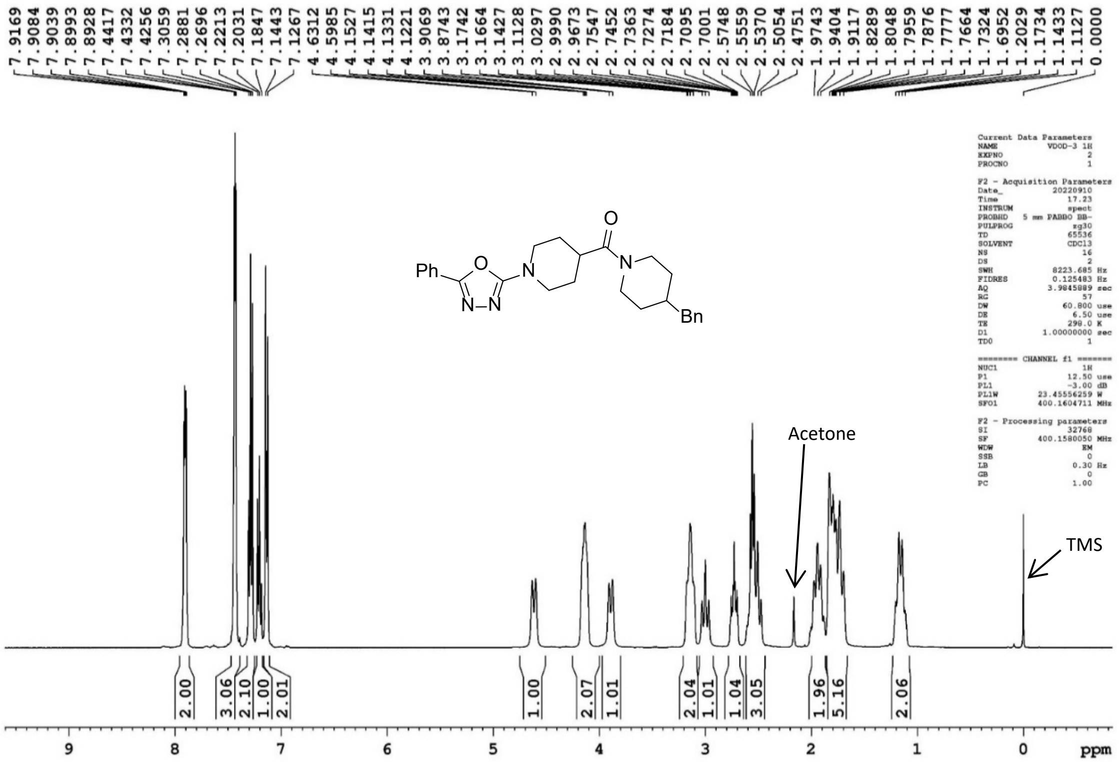

^{1}H NMR spectrum of (4-benzylpiperidin-1-*yl*)(1-(5-phenyl-1,3,4-oxadiazol-2-*yl*)piperidin-4-*yl*)methanone (**P1**).

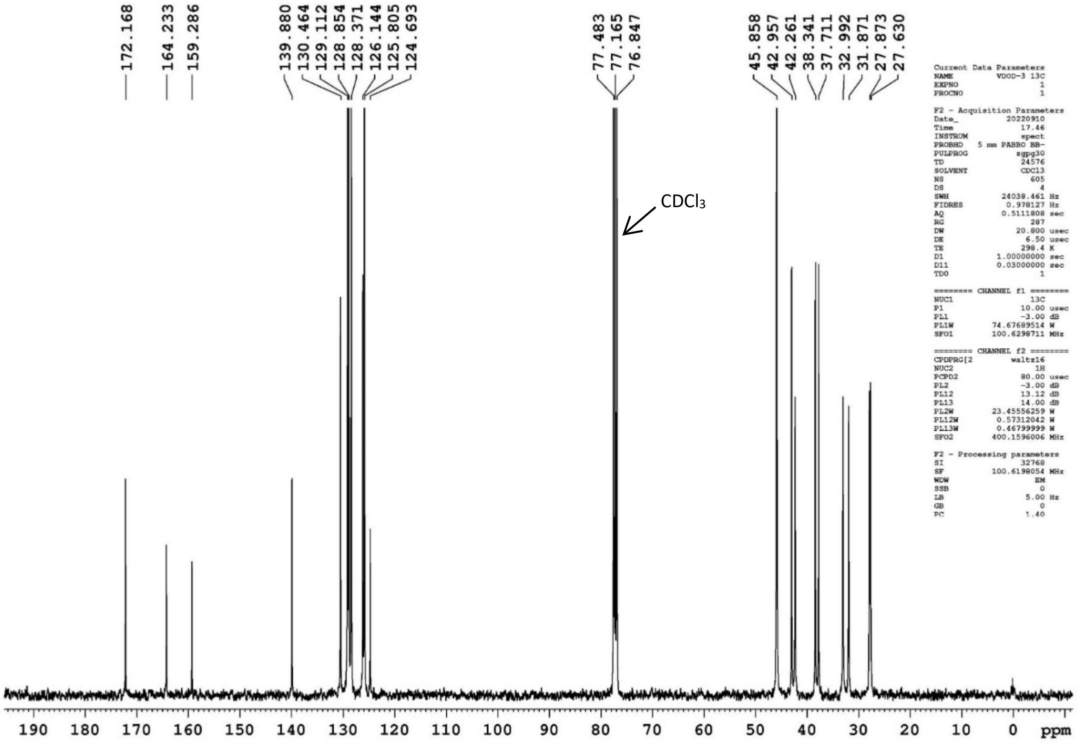

^{13}C NMR spectrum of spectrum of (4-benzylpiperidin-1-*yl*)(1-(5-phenyl-1,3,4-oxadiazol-2-*yl*)piperidin-4-*yl*)methanone (**P1**)

HRMS, HPLC and LRMS of P1

Single Mass Analysis
Tolerance = 5.0 mDa / DBE: min = -1.5, max = 100.0
Element prediction: Off
Number of isotope peaks used for i-FIT = 3

Monoisotopic Mass, Even Electron Ions
83 formula(e) evaluated with 1 results within limits (up to 50 closest results for each mass)
Elements Used:
C: 0-200 H: 0-200 N: 4-4 O: 0-20
VDOD-09JAN23-3 124 (2.114) AM2 (Ar,25000.0,0.00,0.00); ABS
TOF MS ES+

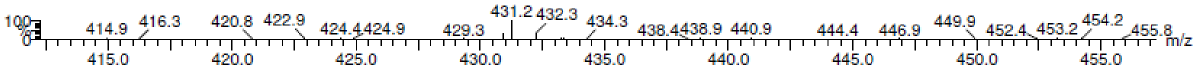

Mass	Calc. Mass	mDa	PPM	DBE	i-FIT	Norm	Conf(%)	Formula
Minimum:				-1.5				
Maximum:		5.0	5.0	100.0				
431.2453	431.2447	0.6	1.4	13.5	454.4	n/a	n/a	C26 H31 N4 O2

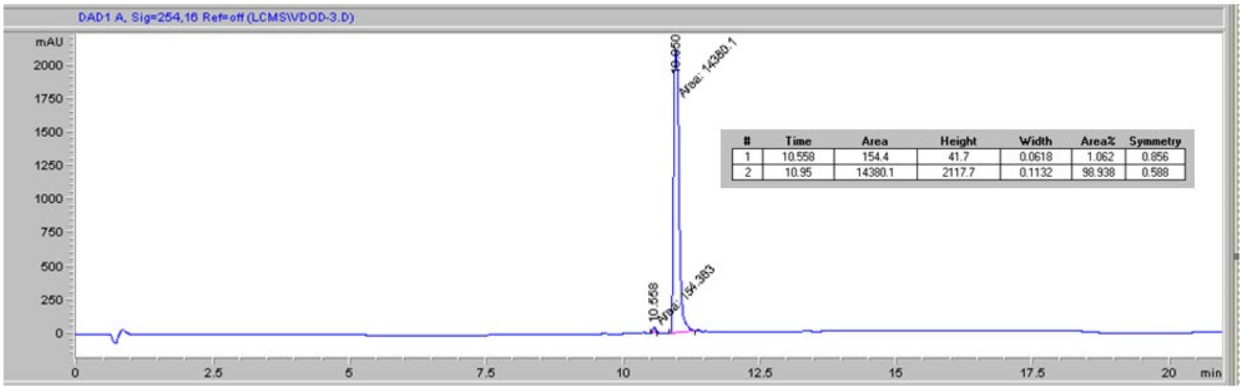

#	Time	Area	Height	Width	Area%	Symmetry
1	10.558	154.4	41.7	0.0618	1.062	0.856
2	10.95	14380.1	2117.7	0.1132	98.938	0.588

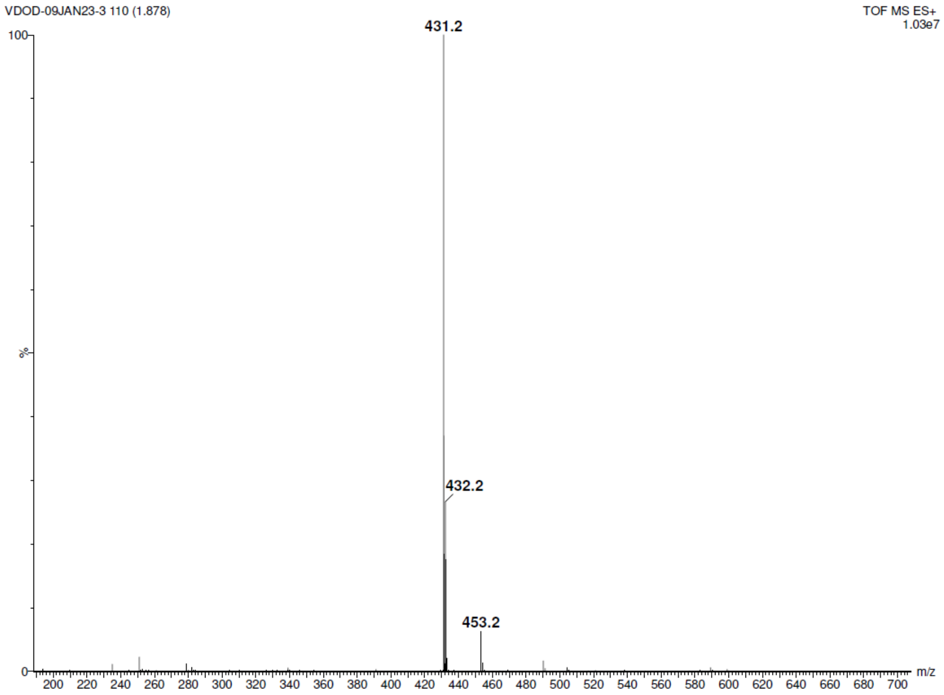

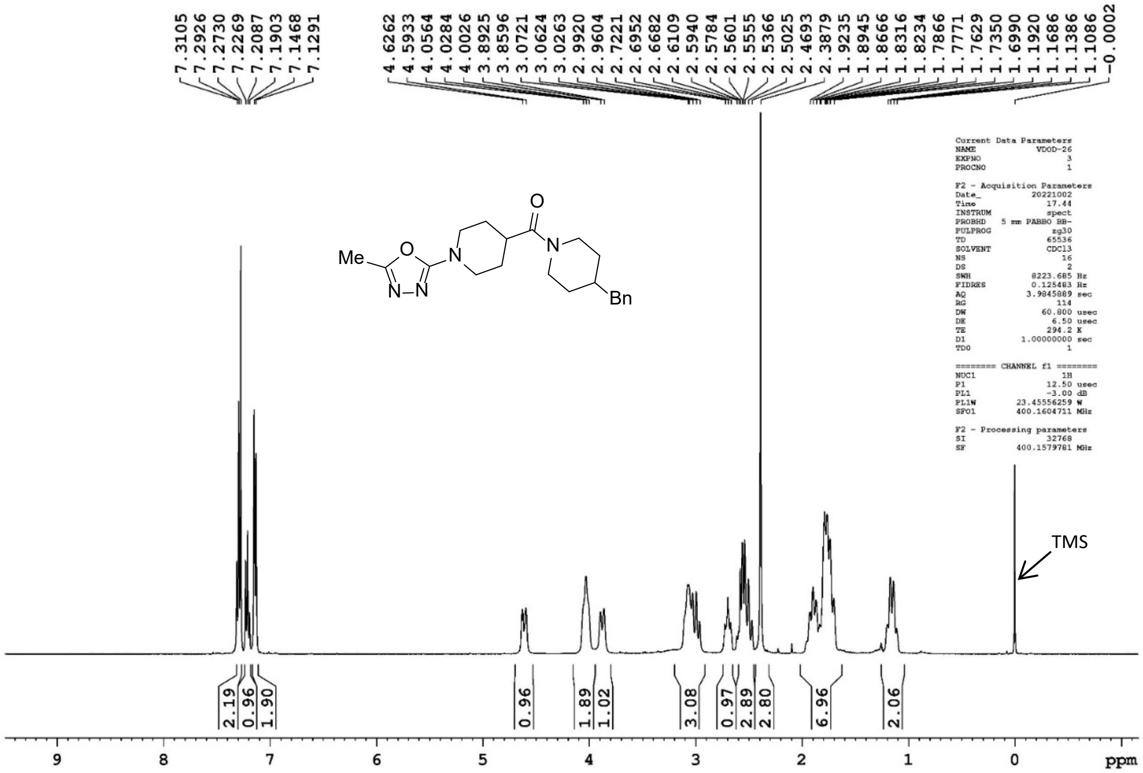

^{1}H NMR spectrum of of (4-benzylpiperidin-1-*yl*)(1-(5-methyl-1,3,4-oxadiazol-2-*yl*)piperidin-4-*yl*)methanone (**P2**)

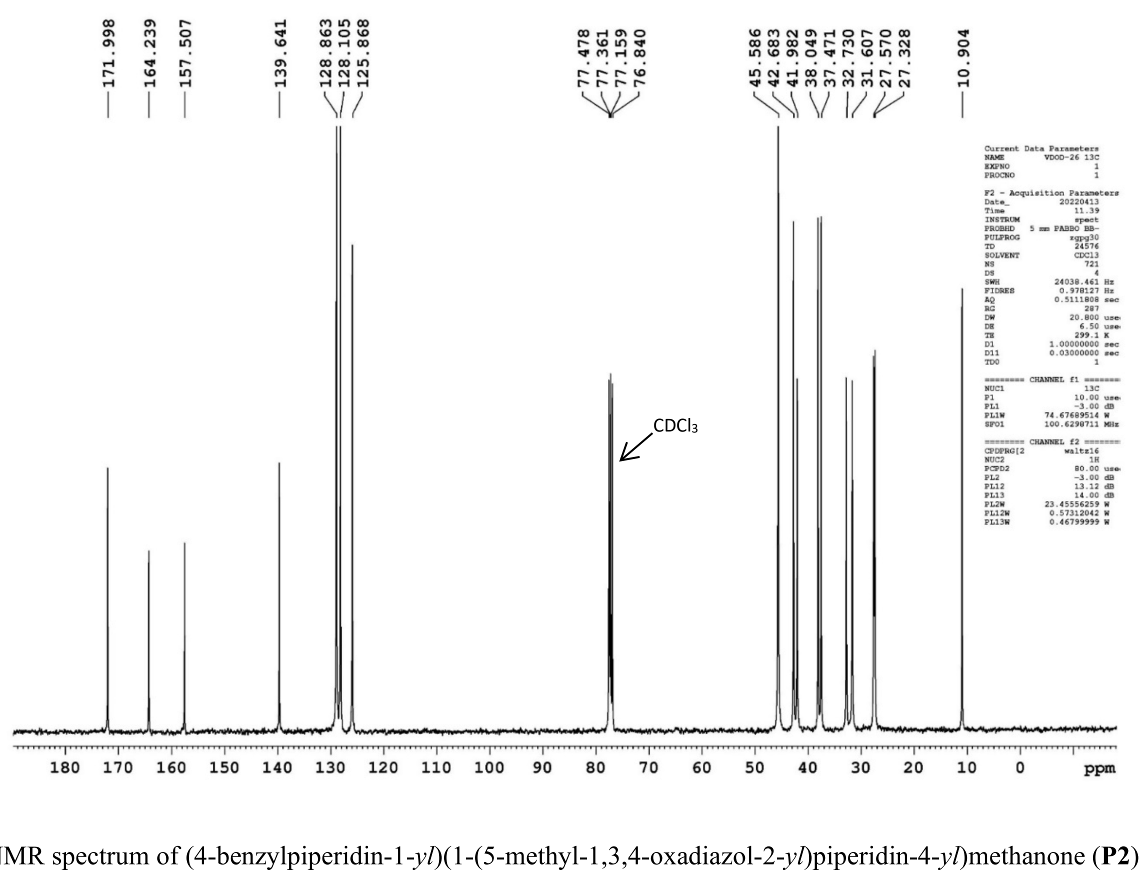

^{13}C NMR spectrum of (4-benzylpiperidin-1-*yl*)(1-(5-methyl-1,3,4-oxadiazol-2-*yl*)piperidin-4-*yl*)methanone (**P2**)

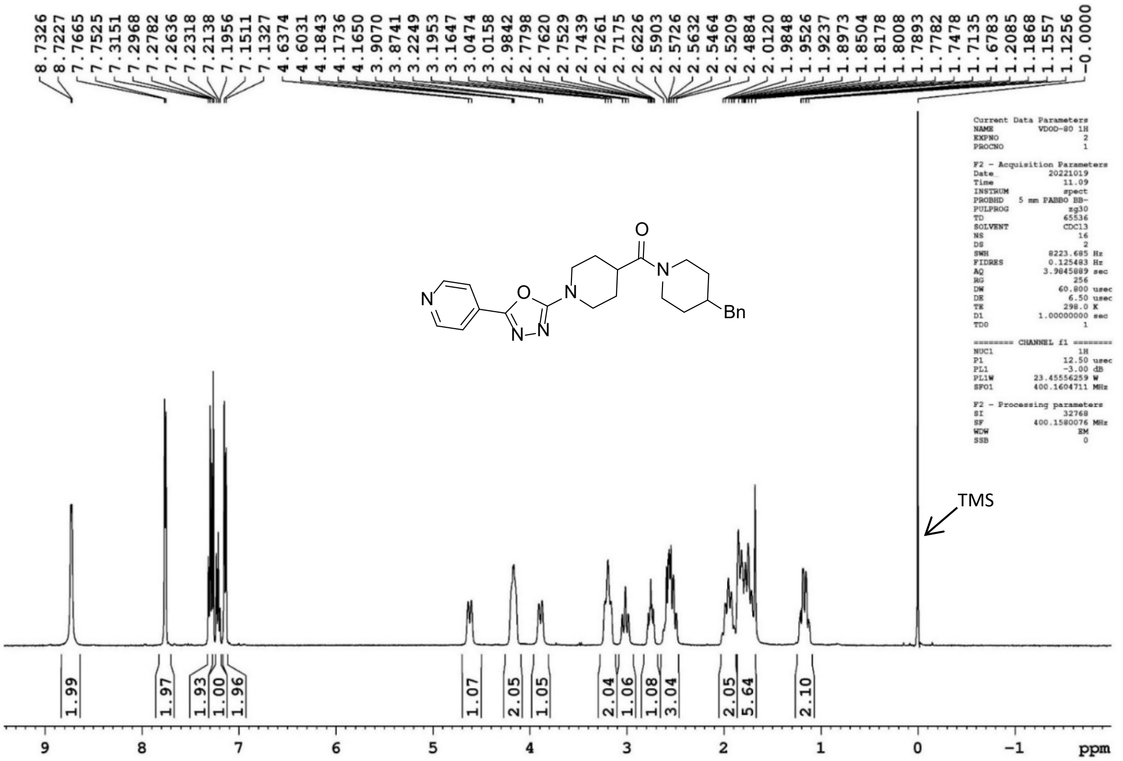

^{1}H NMR spectrum of 4-benzylpiperidin-1-*yl*)(1-(5-(pyridin-4-*yl*)-1,3,4-oxadiazol-2-*yl*)piperidin-4-*yl*)methanone (**P3**)

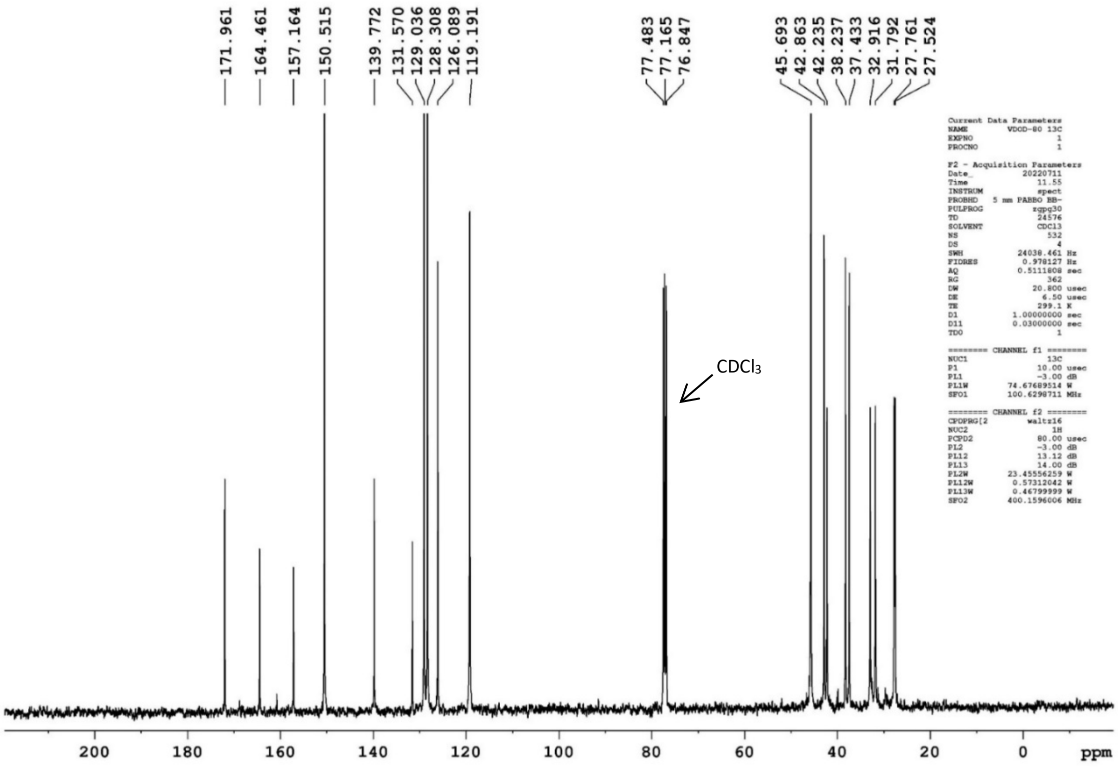

^{13}C NMR spectrum of (4-benzylpiperidin-1-*yl*)(1-(5-(pyridin-4-*yl*)-1,3,4-oxadiazol-2-*yl*)piperidin-4-*yl*)methanone (**P3**)

HRMS, HPLC and LRMS of P3

Single Mass Analysis
Tolerance = 5.0 mDa / DBE: min = -1.5, max = 100.0
Element prediction: Off
Number of isotope peaks used for i-FIT = 3

Monoisotopic Mass, Even Electron Ions
76 formula(e) evaluated with 1 results within limits (up to 50 closest results for each mass)
Elements Used:
C: 0-200 H: 0-200 N: 5-5 O: 0-20
VDOD-09JAN23-80 136 (2.317) AM2 (Ar,25000.0,0.00,0.00); ABS
TOF MS ES+

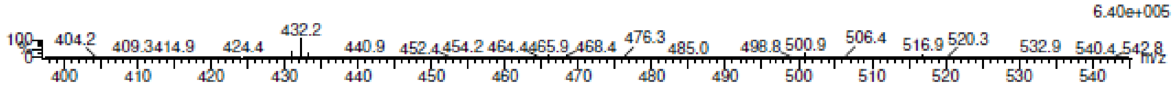

Minimum:				-1.5				
Maximum:		5.0	5.0	100.0				
Mass	Calc. Mass	mDa	PPM	DBE	i-FIT	Norm	Conf(%)	Formula
432.2406	432.2400	0.6	1.4	13.5	452.6	n/a	n/a	C25 H30 N5 O2

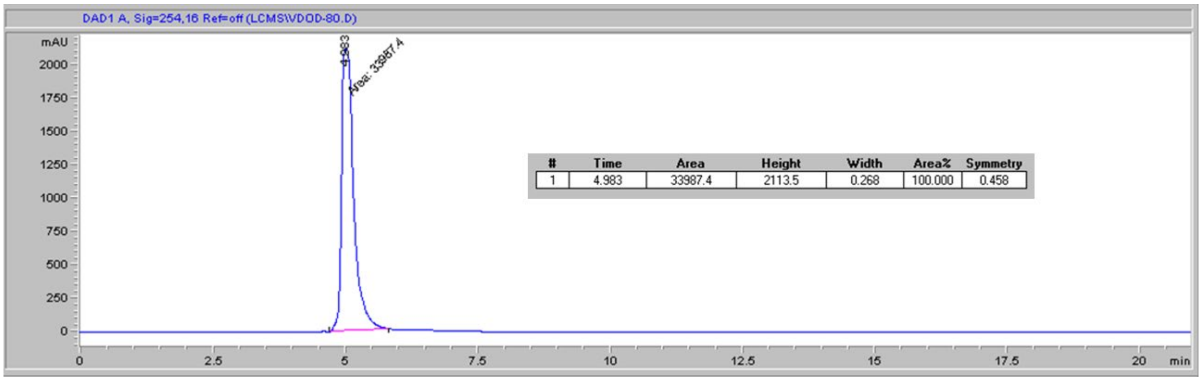

#	Time	Area	Height	Width	Area%	Symmetry
1	4.983	33987.4	2113.5	0.268	100.000	0.458

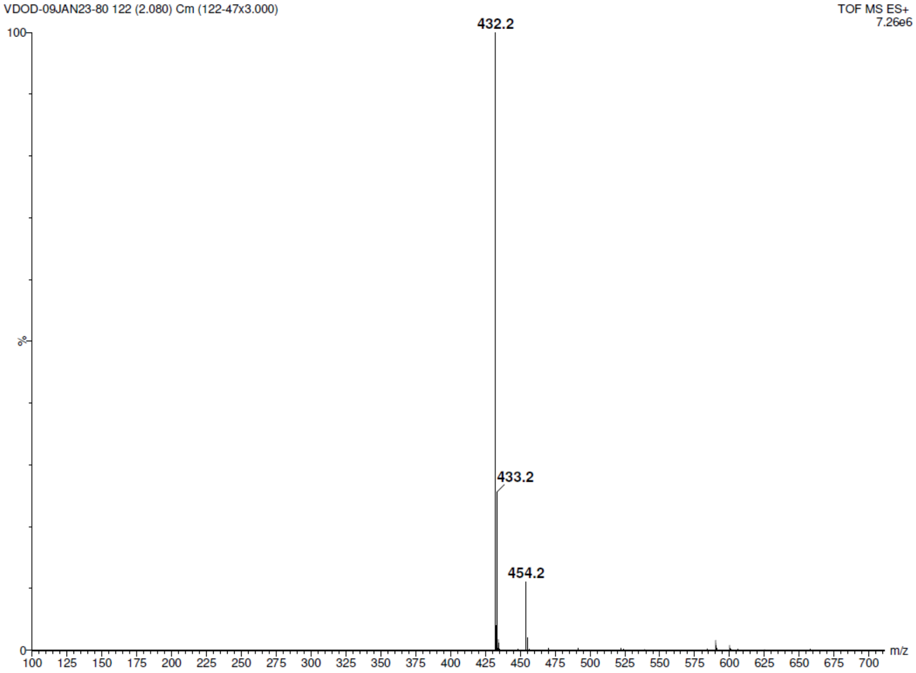

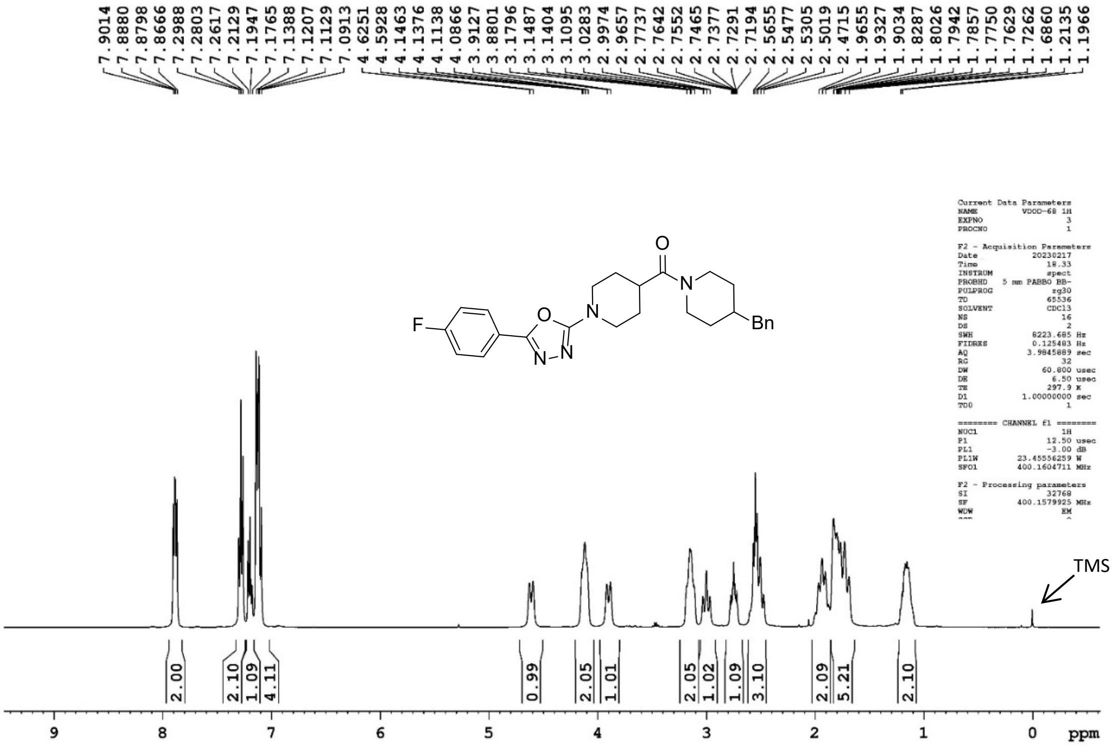

^{1}H NMR spectrum of (4-benzylpiperidin-1-*yl*)(1-(5-(4-fluorophenyl)-1,3,4-oxadiazol-2-*yl*)piperidin-4-*yl*)methanone (**P4**)

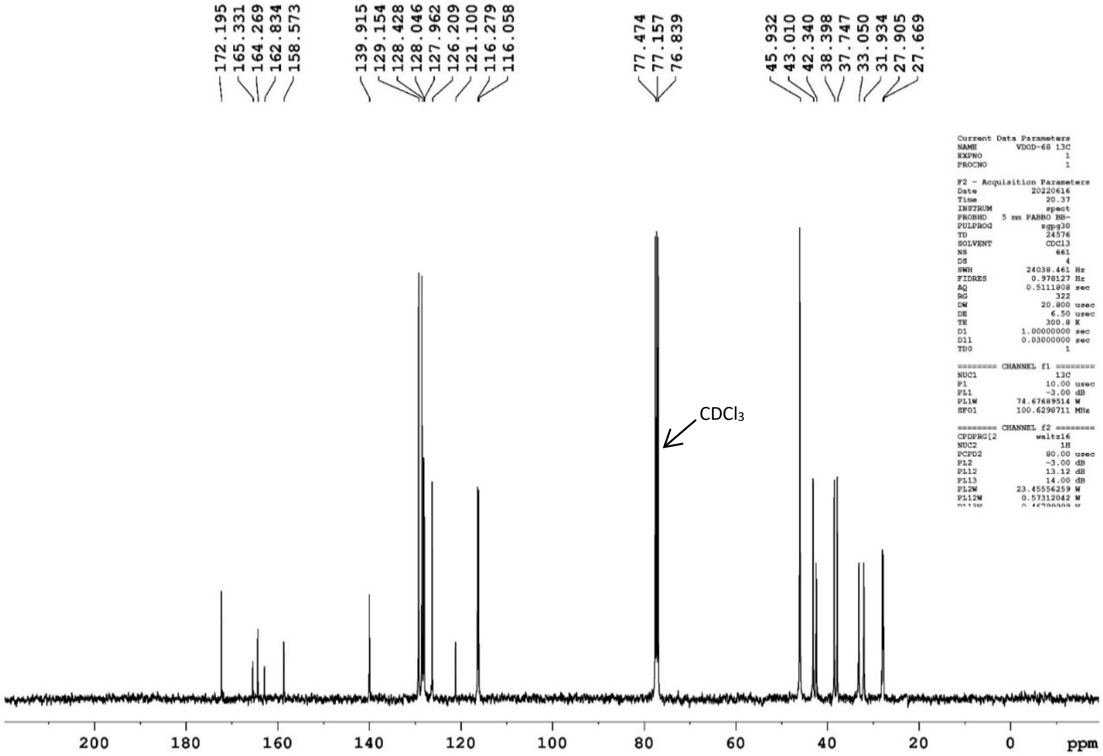

^{13}C NMR spectrum of (4-benzylpiperidin-1-*yl*)(1-(5-(4-fluorophenyl)-1,3,4-oxadiazol-2-*yl*)piperidin-4-*yl*)methanone (**P4**)

HRMS, HPLC and LRMS of P4

Single Mass Analysis
Tolerance = 5.0 mDa / DBE: min = -1.5, max = 100.0
Element prediction: Off
Number of isotope peaks used for i-FIT = 3

Monoisotopic Mass, Even Electron Ions
76 formula(e) evaluated with 1 results within limits (up to 50 closest results for each mass)
Elements Used:
C: 0-200 H: 0-200 N: 4-4 O: 0-20 F: 1-1
VDOD-09JAN23-68 181 (3.079) AM2 (Ar,25000.0,0.00,0.00); ABS
TOF MS ES+

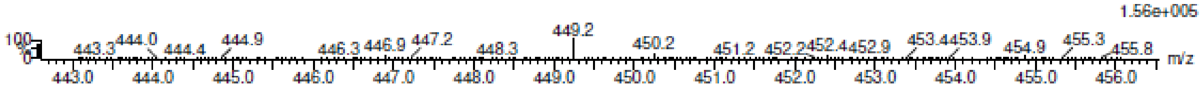

Minimum:				-1.5				
Maximum:		5.0	5.0	100.0				
Mass	Calc. Mass	mDa	PPM	DBE	i-FIT	Norm	Conf(%)	Formula
449.2357	449.2353	0.4	0.9	13.5	456.8	n/a	n/a	C26 H30 N4 O2 F

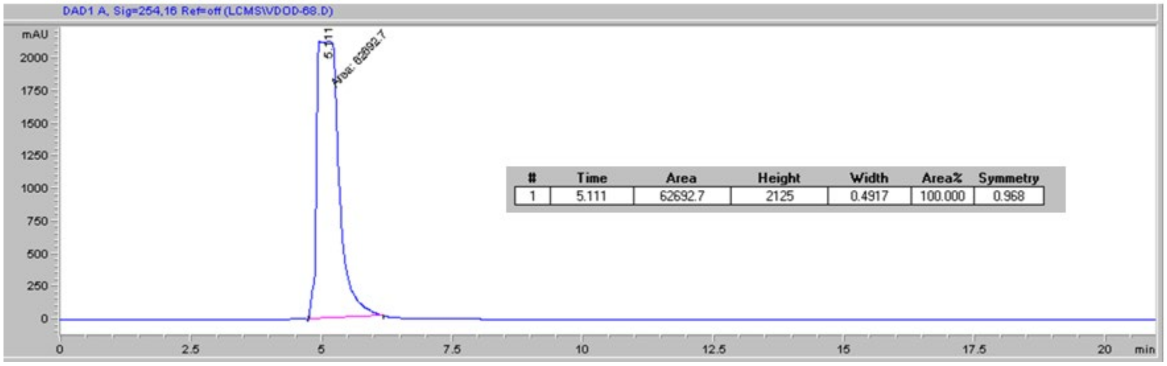

#	Time	Area	Height	Width	Area%	Symmetry
1	5.111	62692.7	2125	0.4917	100.000	0.968

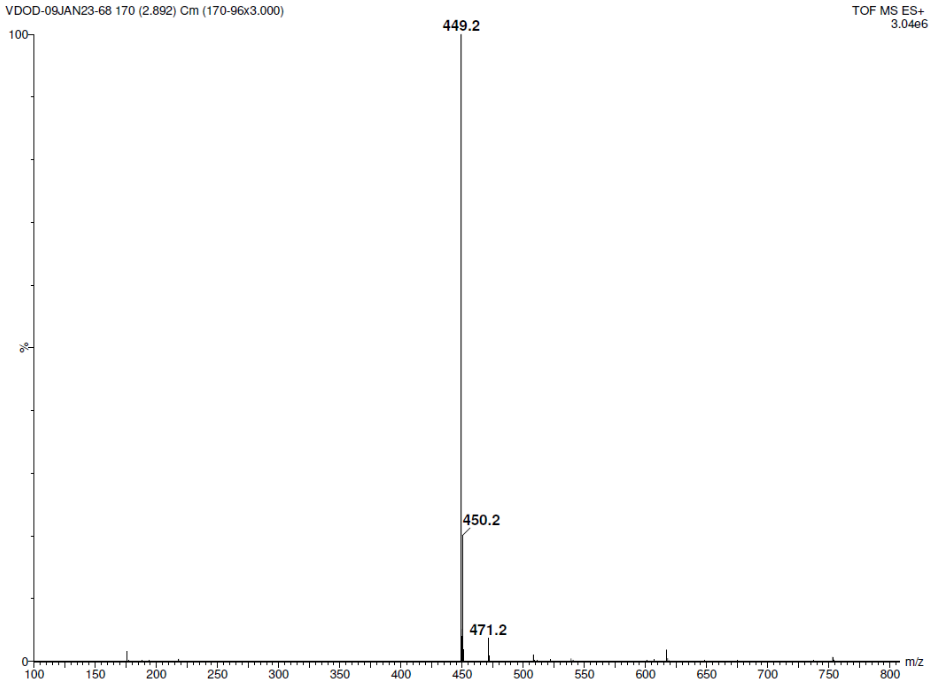

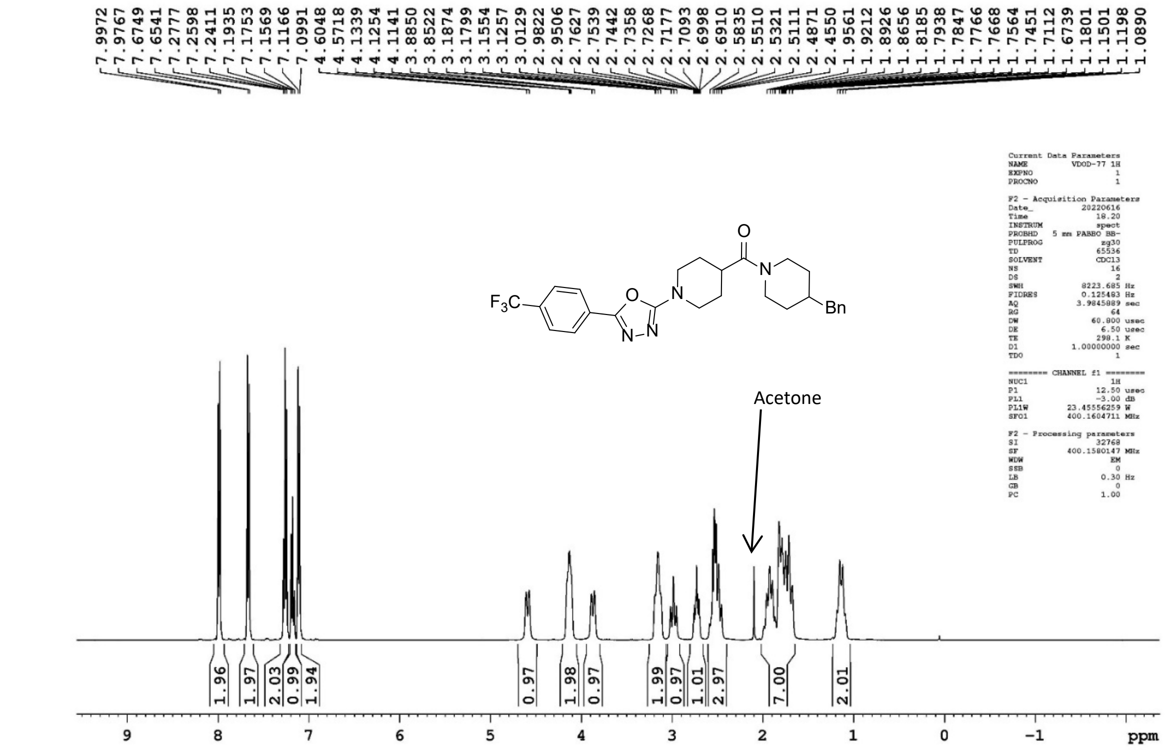

^{1}H NMR spectrum of (4-benzylpiperidin-1-*yl*)(1-(5-(4-(trifluoromethyl)phenyl)-1,3,4-oxadiazol-2-*yl*)piperidin-4-*yl*)methanone (**P5**).

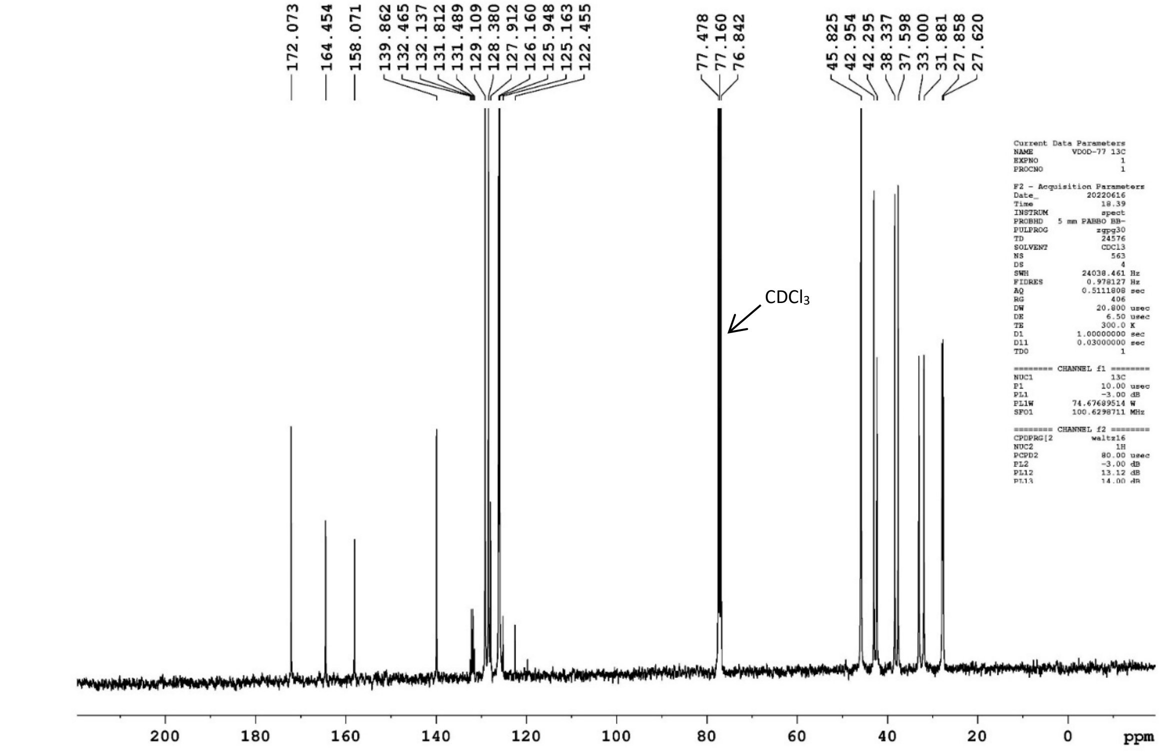

^{13}C NMR spectrum of (4-benzylpiperidin-1-*yl*)(1-(5-(4-(trifluoromethyl)phenyl)-1,3,4-oxadiazol-2-*yl*)piperidin-4-*yl*)methanone (**P5**)

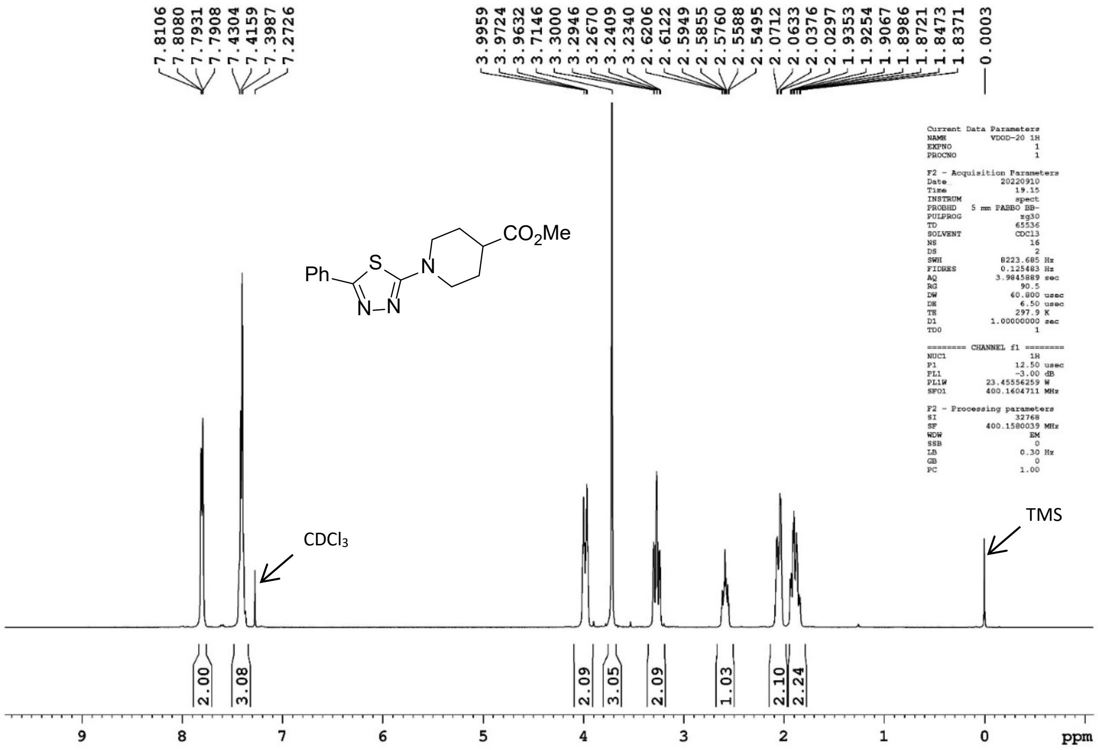

^{1}H NMR spectrum of methyl 1-(5-phenyl-1,3,4-thiadiazol-2-*yl*)piperidine-4-carboxylate (**5a**)

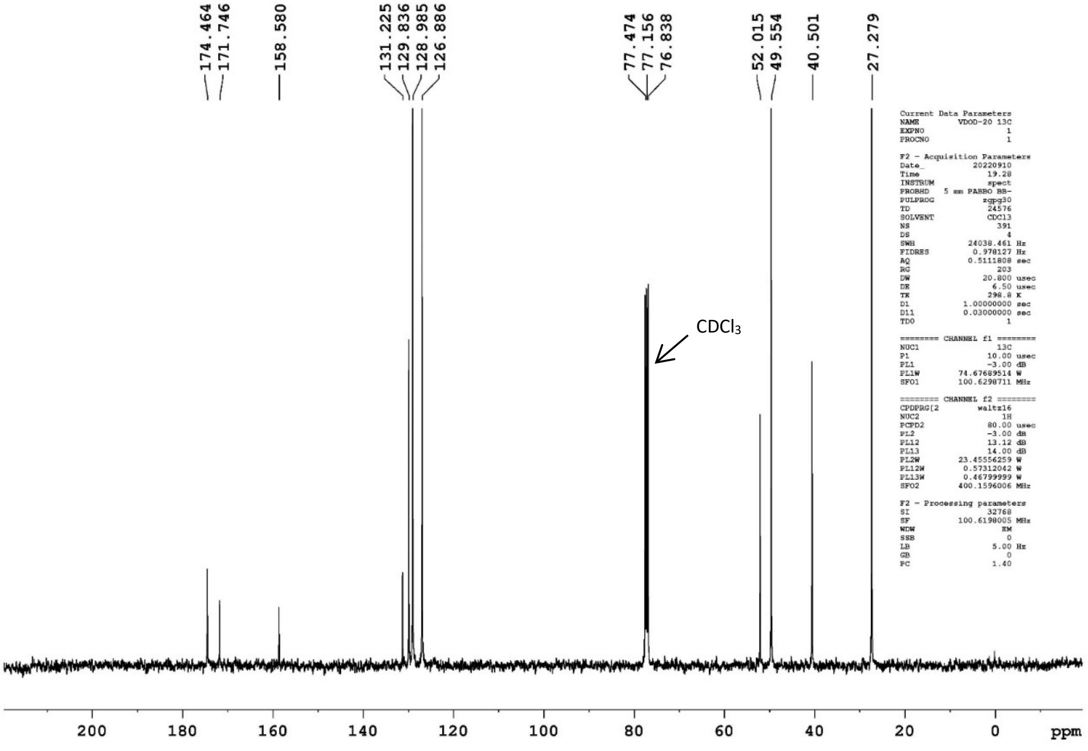

^{13}C NMR spectrum of methyl 1-(5-phenyl-1,3,4-thiadiazol-2-*yl*)piperidine-4-carboxylate (**5a**)

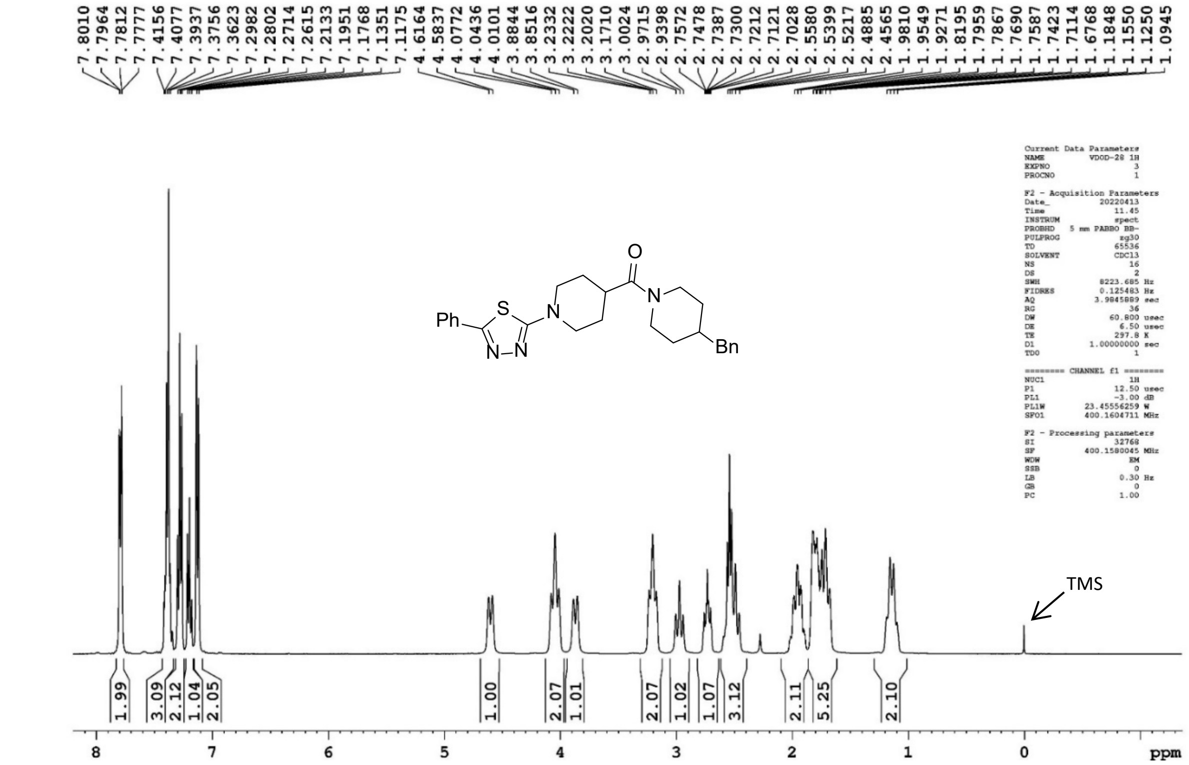

^{1}H NMR spectrum of (4-benzylpiperidin-1-*yl*)(1-(5-phenyl-1,3,4-thiadiazol-2-*yl*)piperidin-4-*yl*)methanone (**P6**)

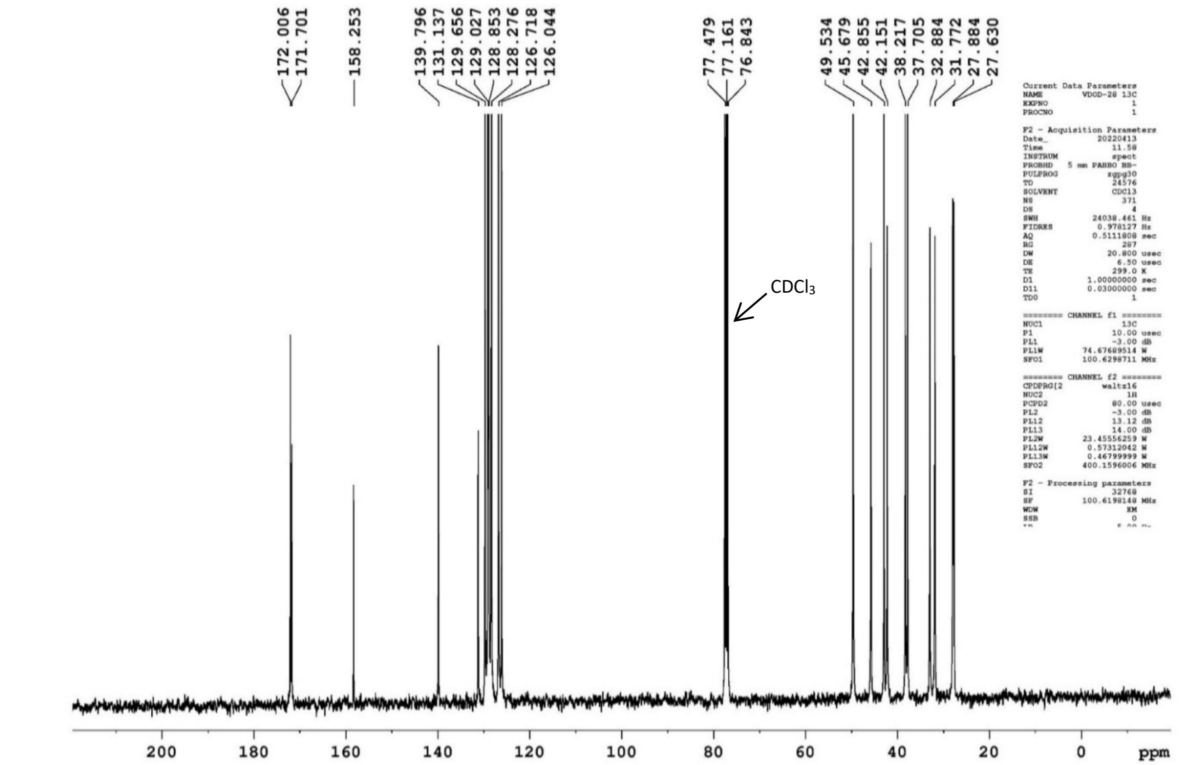

^{13}C NMR spectrum of (4-benzylpiperidin-1-*yl*)(1-(5-phenyl-1,3,4-thiadiazol-2-*yl*)piperidin-4-*yl*)methanone (**P6**)

HRMS, HPLC and LRMS of P6

Single Mass Analysis
Tolerance = 5.0 mDa / DBE: min = -1.5, max = 100.0
Element prediction: Off
Number of isotope peaks used for i-FIT = 3

Monoisotopic Mass, Even Electron Ions
79 formula(e) evaluated with 1 results within limits (up to 50 closest results for each mass)
Elements Used:
C: 0-200 H: 0-200 N: 4-4 O: 0-20 32S: 1-1
VDOD-09JAN23-28 169 (2.875) AM2 (Ar,25000.0,0.00,0.00); ABS
TOF MS ES+

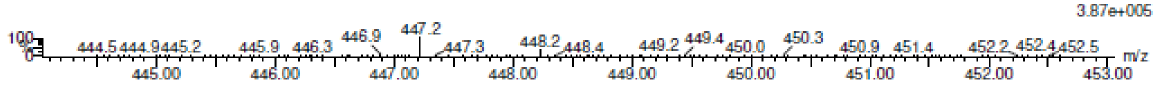

Minimum:				-1.5				
Maximum:		5.0	5.0	100.0				
Mass	Calc. Mass	mDa	PPM	DBE	i-FIT	Norm	Conf(%)	Formula
447.2227	447.2219	0.8	1.8	13.5	441.2	n/a	n/a	C26 H31 N4 O 32S

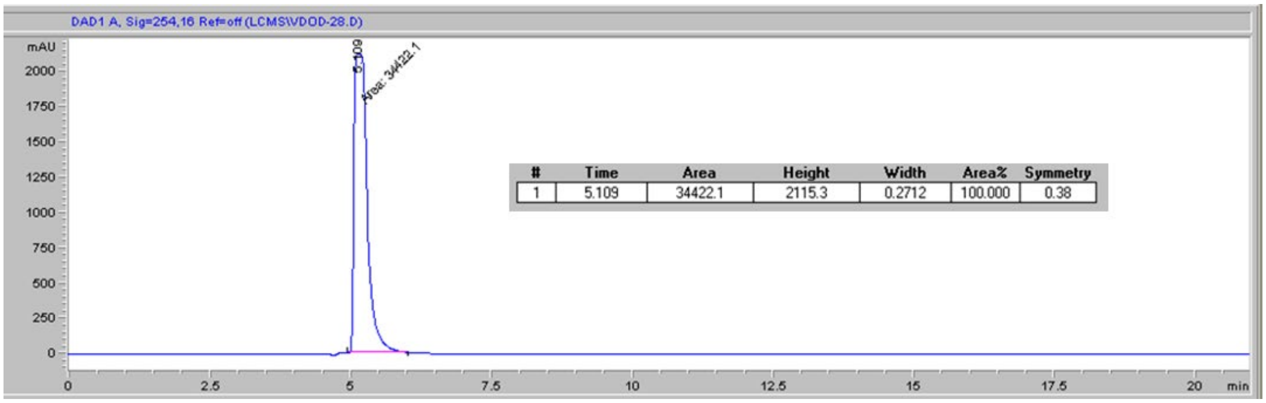

#	Time	Area	Height	Width	Area%	Symmetry
1	5.109	34422.1	2115.3	0.2712	100.000	0.38

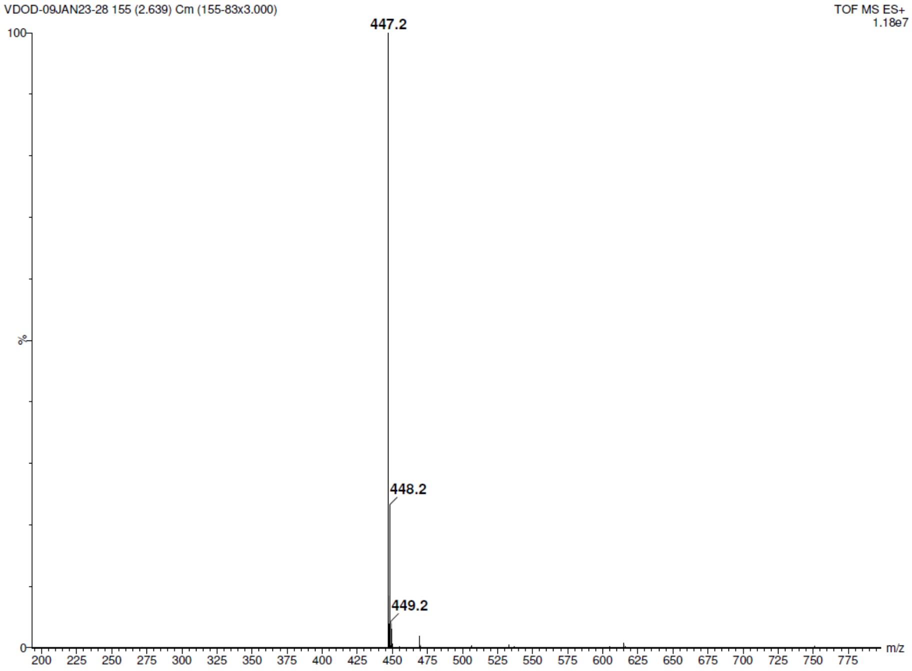

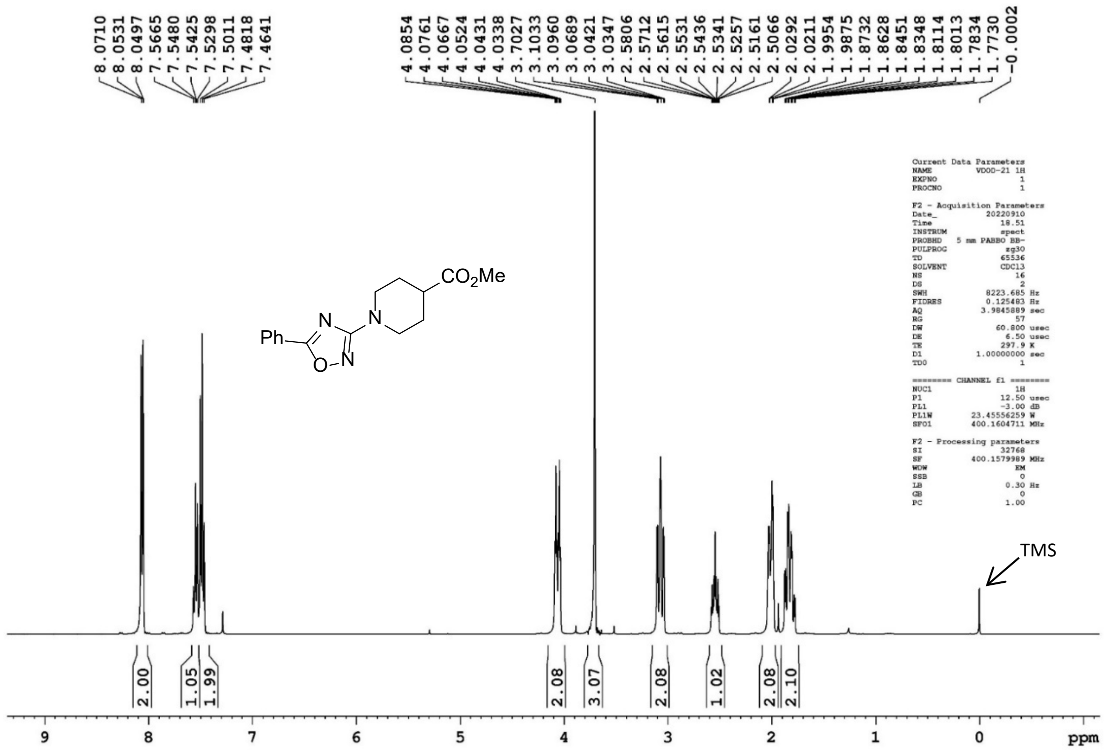

^{1}H NMR spectrum of methyl 1-(5-phenyl-1,2,4-oxadiazol-3-*yl*)piperidine-4-carboxylate (**5b**)

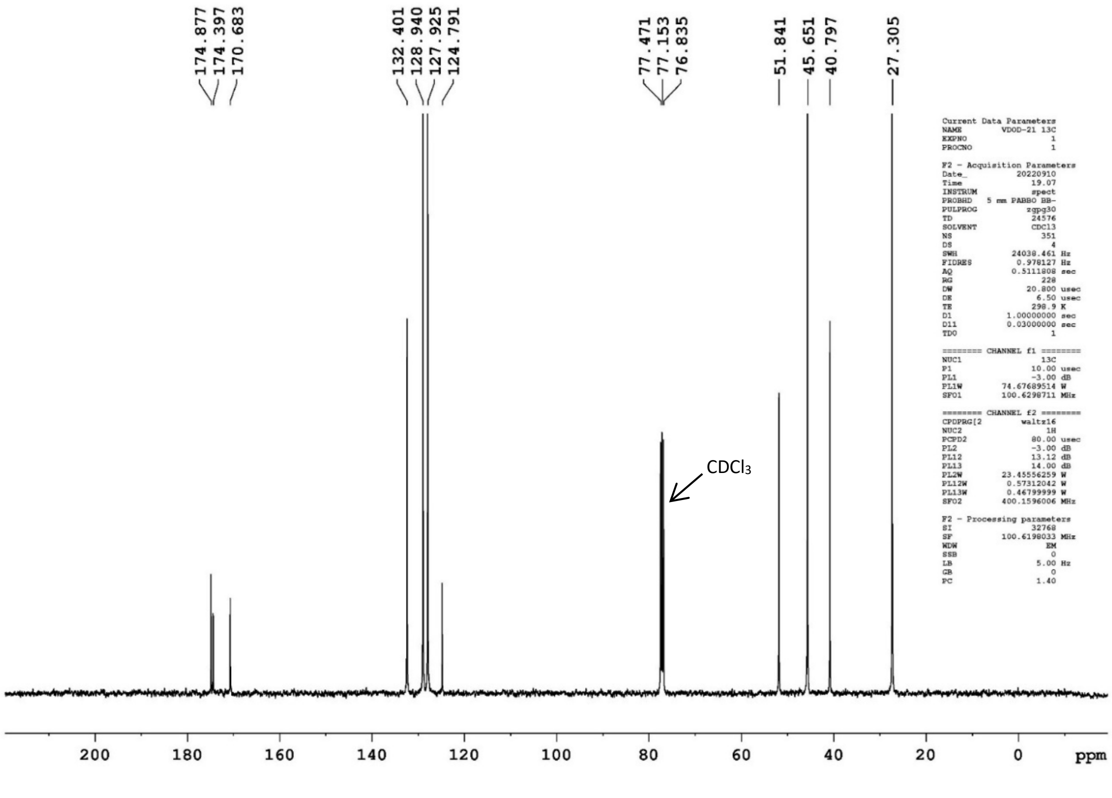

^{13}C NMR spectrum of methyl 1-(5-phenyl-1,2,4-oxadiazol-3-*yl*)piperidine-4-carboxylate (**5b**)

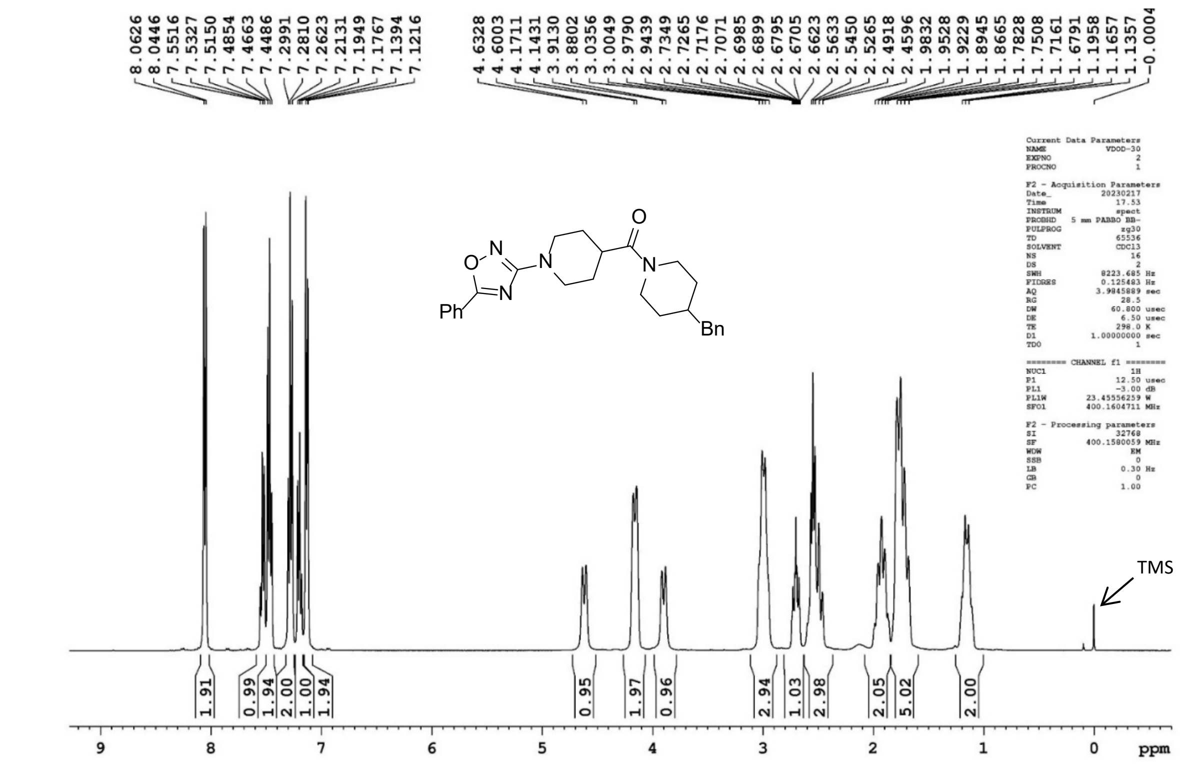

^{1}H NMR spectrum of (4-benzylpiperidin-1-*yl*)(1-(5-phenyl-1,2,4-oxadiazol-3-*yl*)piperidin-4-*yl*)methanone (**P7**)

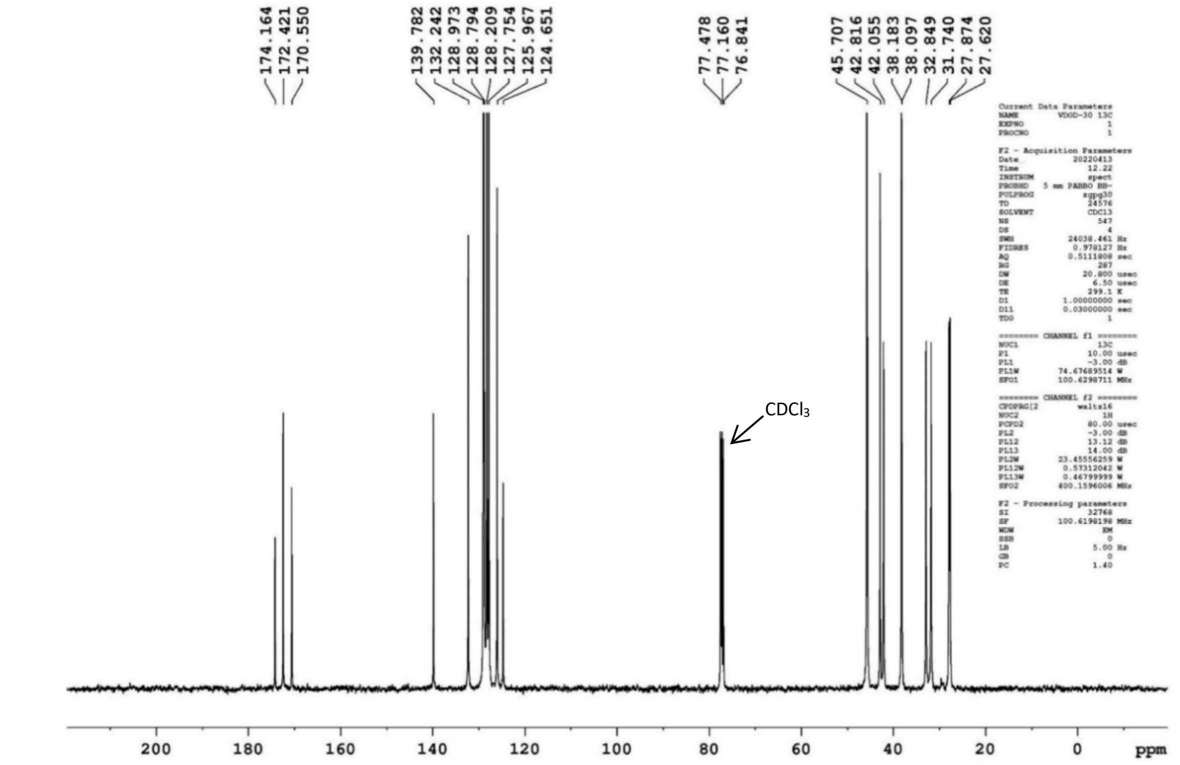

^{13}C NMR spectrum of (4-benzylpiperidin-1-*yl*)(1-(5-phenyl-1,2,4-oxadiazol-3-*yl*)piperidin-4-*yl*)methanone (**P7**)

HRMS, HPLC and LRMS of P7

Single Mass Analysis
Tolerance = 5.0 mDa / DBE: min = -1.5, max = 100.0
Element prediction: Off
Number of isotope peaks used for i-FIT = 3

Monoisotopic Mass, Even Electron Ions
83 formula(e) evaluated with 1 results within limits (up to 50 closest results for each mass)
Elements Used:
C: 0-200 H: 0-200 N: 4-4 O: 0-20
VDOD-09JAN23-30 204 (3.468) AM2 (Ar,25000.0,0.00,0.00); ABS
TOF MS ES+

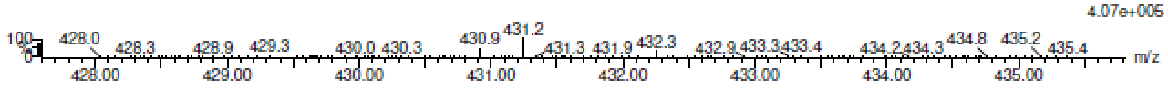

Minimum:				-1.5				
Maximum:		5.0	5.0	100.0				
Mass	Calc. Mass	mDa	PPM	DBE	i-FIT	Norm	Conf(%)	Formula
431.2452	431.2447	0.5	1.2	13.5	452.3	n/a	n/a	C26 H31 N4 O2

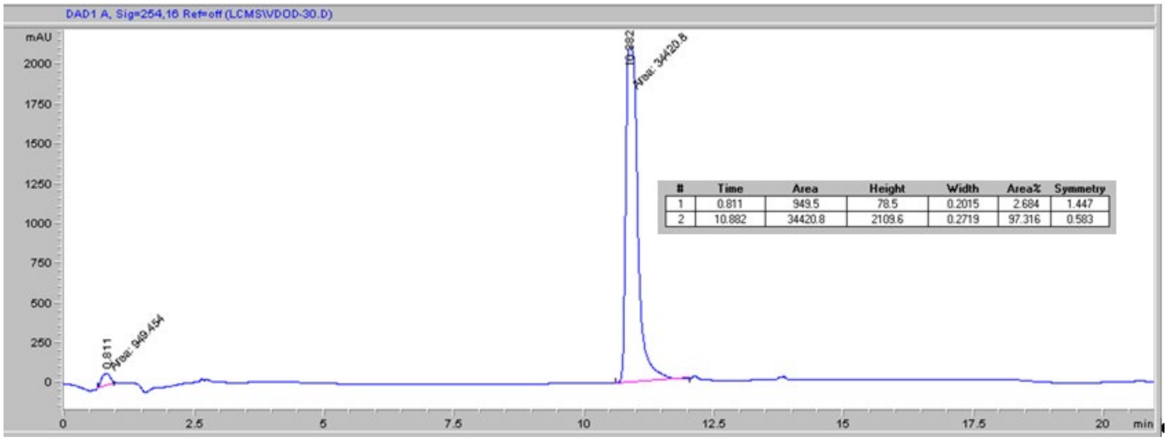

#	Time	Area	Height	Width	Area%	Symmetry
1	0.811	949.5	78.5	0.2015	2.684	1.447
2	10.882	34420.8	2109.6	0.2719	97.316	0.583

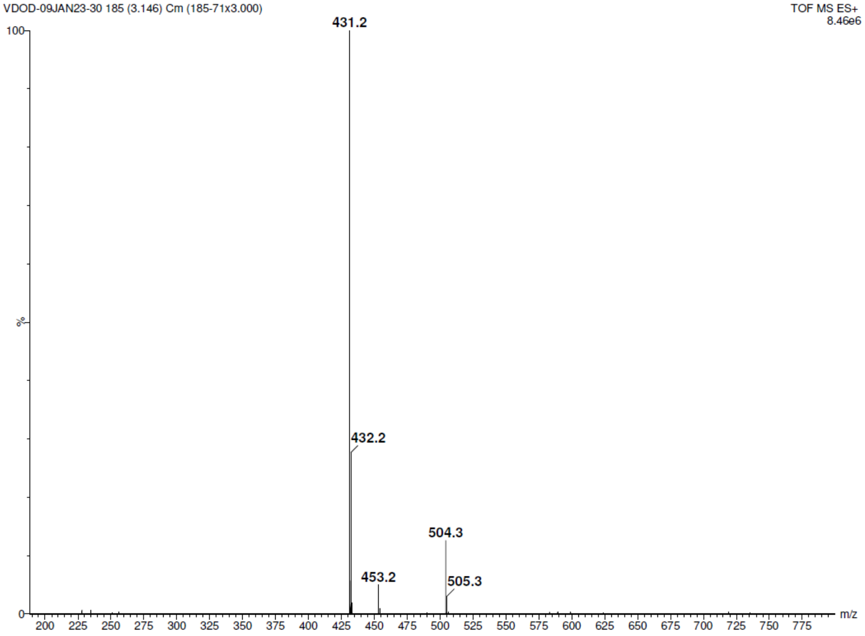

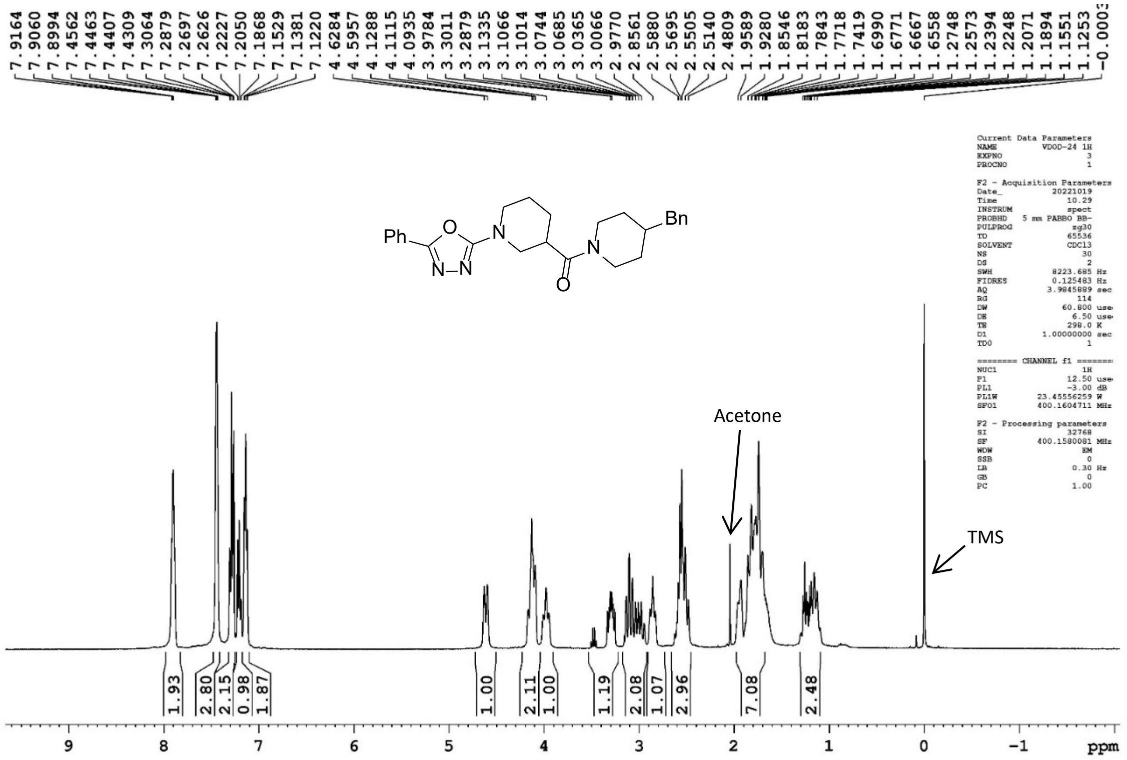

^{1}H NMR spectrum of (4-benzylpiperidin-1-*yl*)(1-(5-phenyl-1,3,4-oxadiazol-2-*yl*)piperidin-3-*yl*)methanone **(P8)**

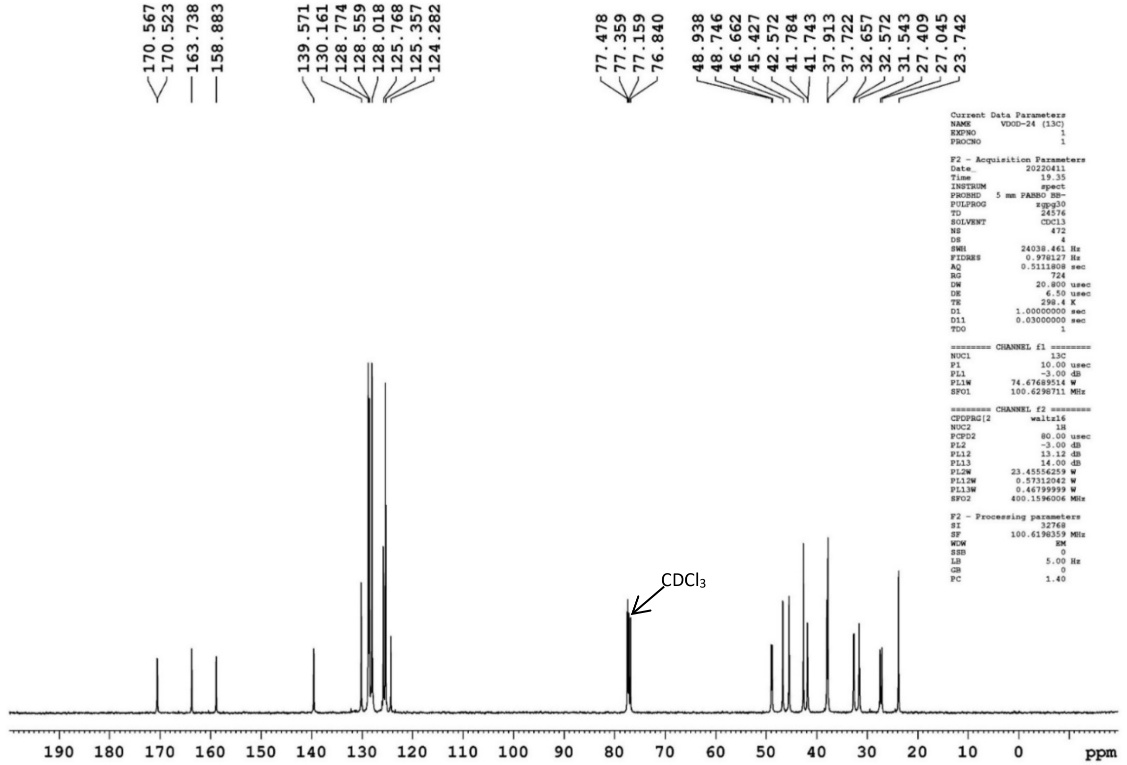

^{13}C NMR spectrum of (4-benzylpiperidin-1-*yl*)(1-(5-phenyl-1,3,4-oxadiazol-2-*yl*)piperidin-3-*yl*)methanone **(P8)**

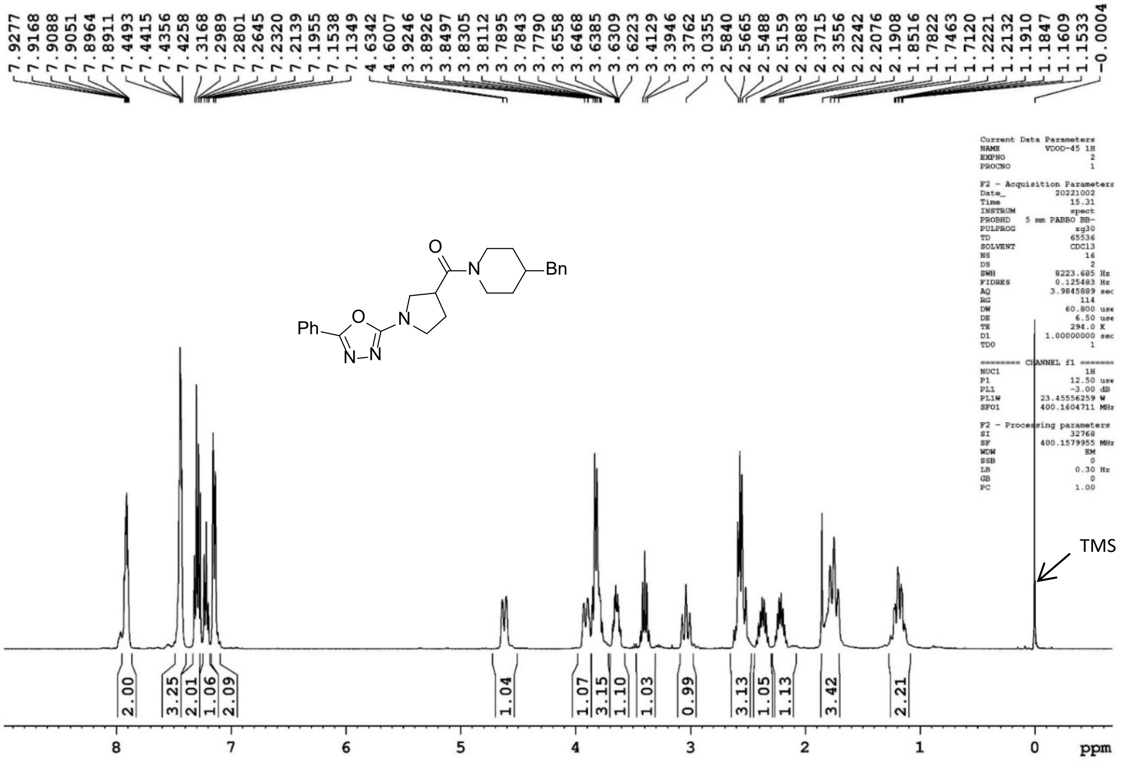

^{1}H NMR spectrum of (4-benzylpiperidin-1-*yl*)(1-(5-phenyl-1,3,4-oxadiazol-2-*yl*)pyrrolidin-3-*yl*)methanone **(P9)**

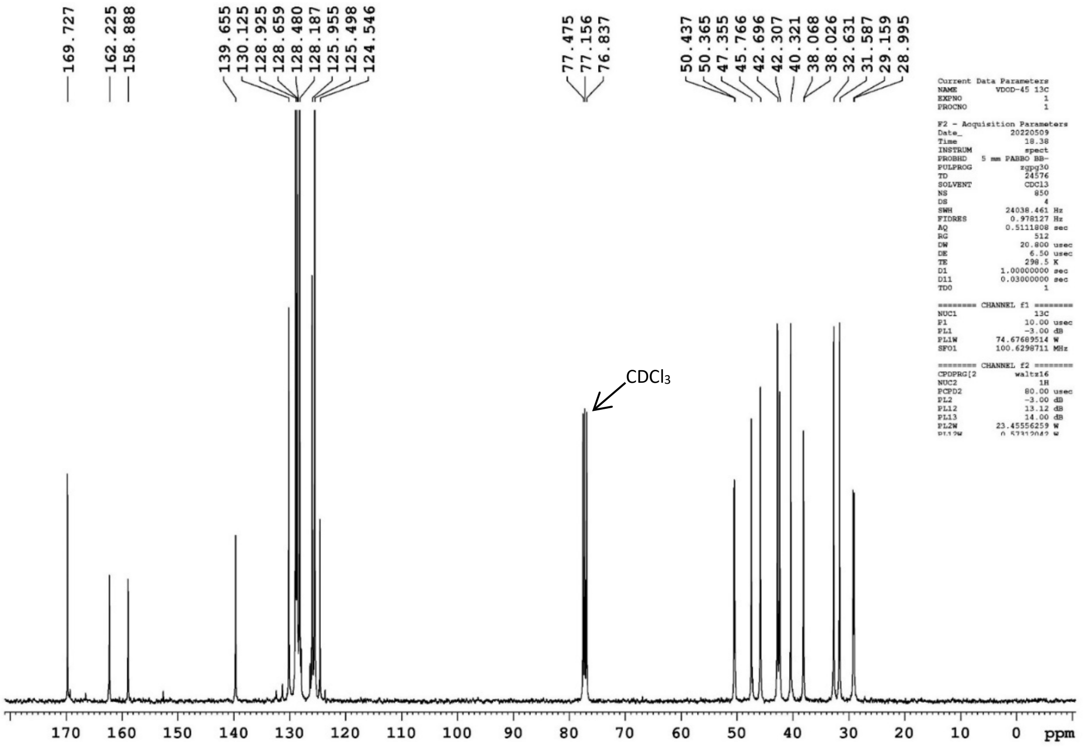

^{13}C NMR spectrum of (4-benzylpiperidin-1-*yl*)(1-(5-phenyl-1,3,4-oxadiazol-2-*yl*)pyrrolidin-3-*yl*)methanone **(P9)**

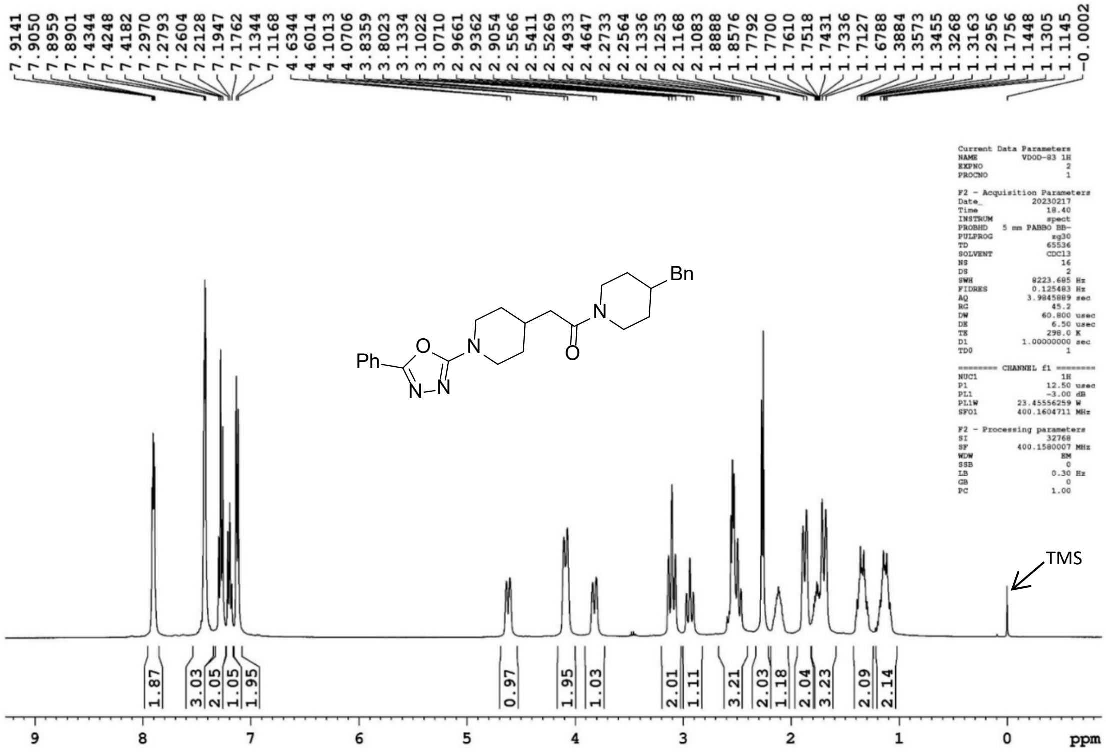

^{1}H NMR spectrum of 1-(4-benzylpiperidin-1-*yl*)-2-(1-(5-phenyl-1,3,4-oxadiazol-2-*yl*)piperidin-4-*yl*)ethan-1-one **(P10)**

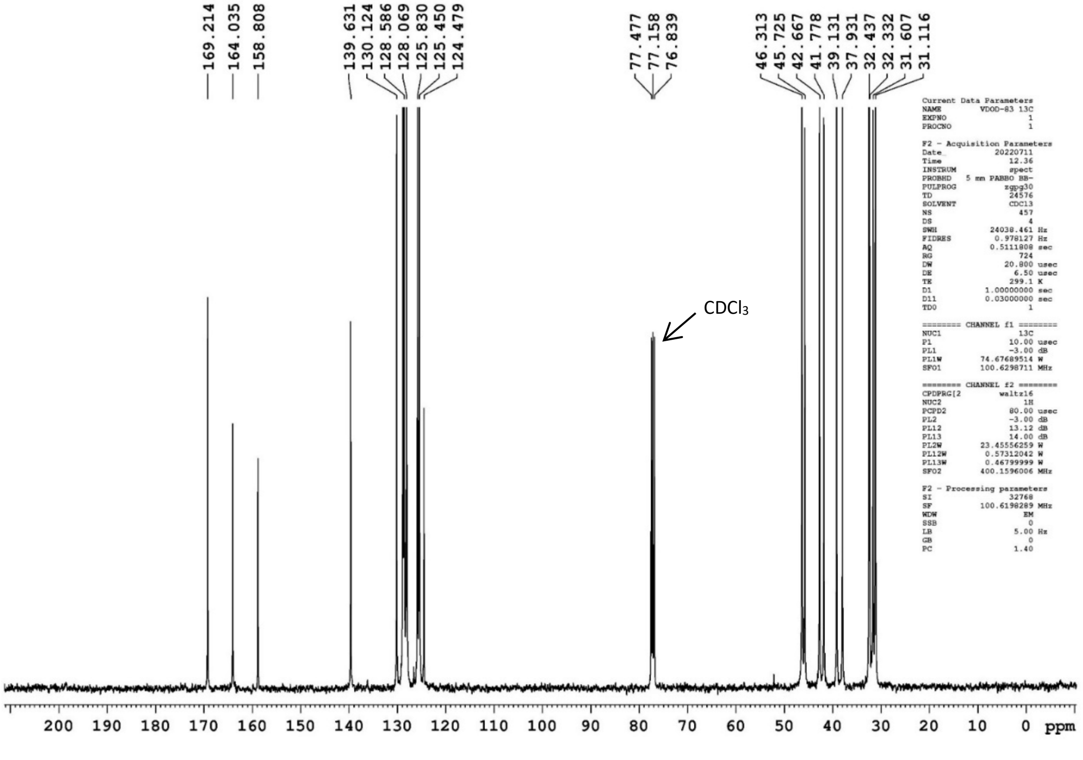

^{13}C NMR spectrum of 1-(4-benzylpiperidin-1-*yl*)-2-(1-(5-phenyl-1,3,4-oxadiazol-2-*yl*)piperidin-4-*yl*)ethan-1-one **(P10)**

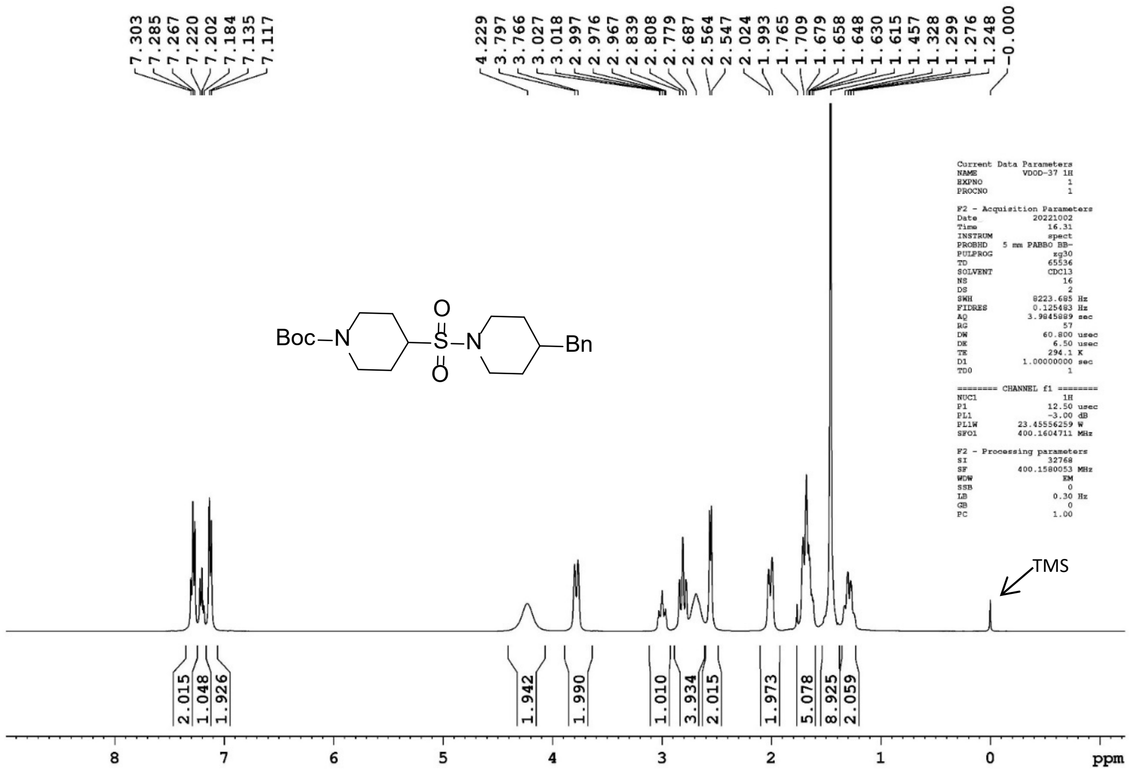

^{1}H NMR spectrum of *tert*-butyl 4-((4-benzylpiperidin-1-*yl*)sulfonyl)piperidine-1-carboxylate (**7**)

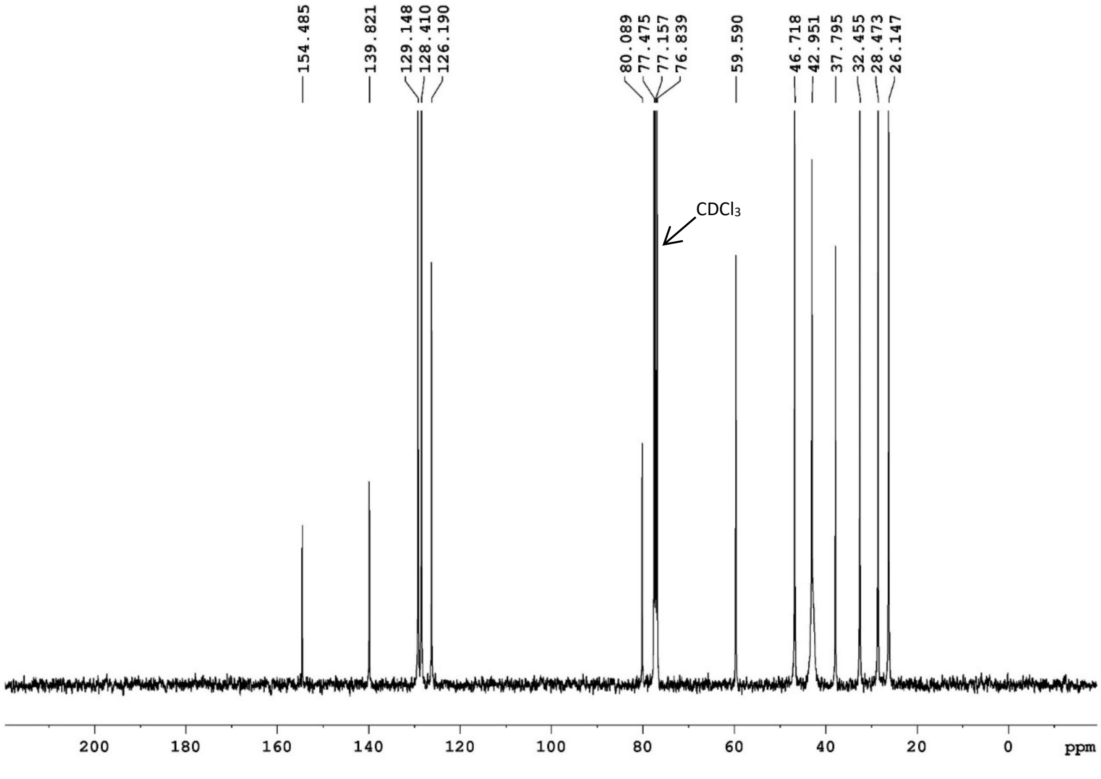

^{13}C NMR spectrum of *tert*-butyl 4-((4-benzylpiperidin-1-*yl*)sulfonyl)piperidine-1-carboxylate (**7**)

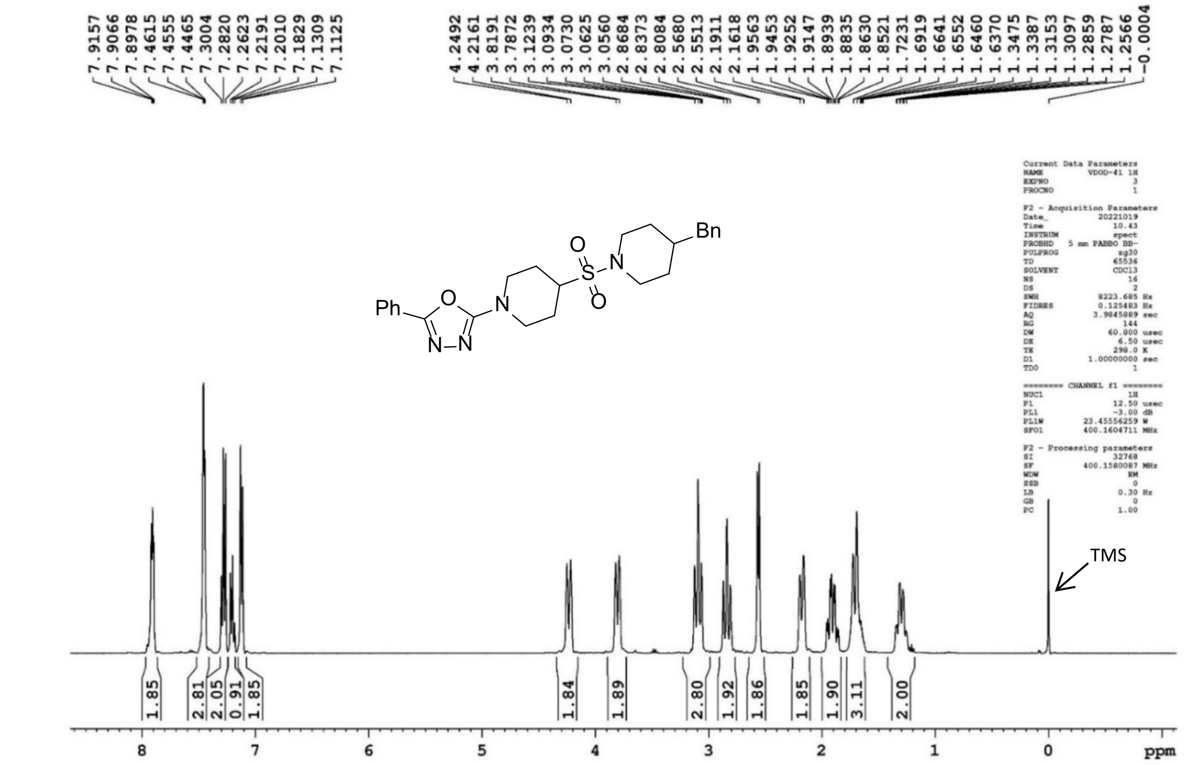

^{1}H NMR spectrum of 2-(4-((4-benzylpiperidin-1-*yl*)sulfonyl)piperidin-1-*yl*)-5-phenyl-1,3,4-oxadiazole **(P11)**

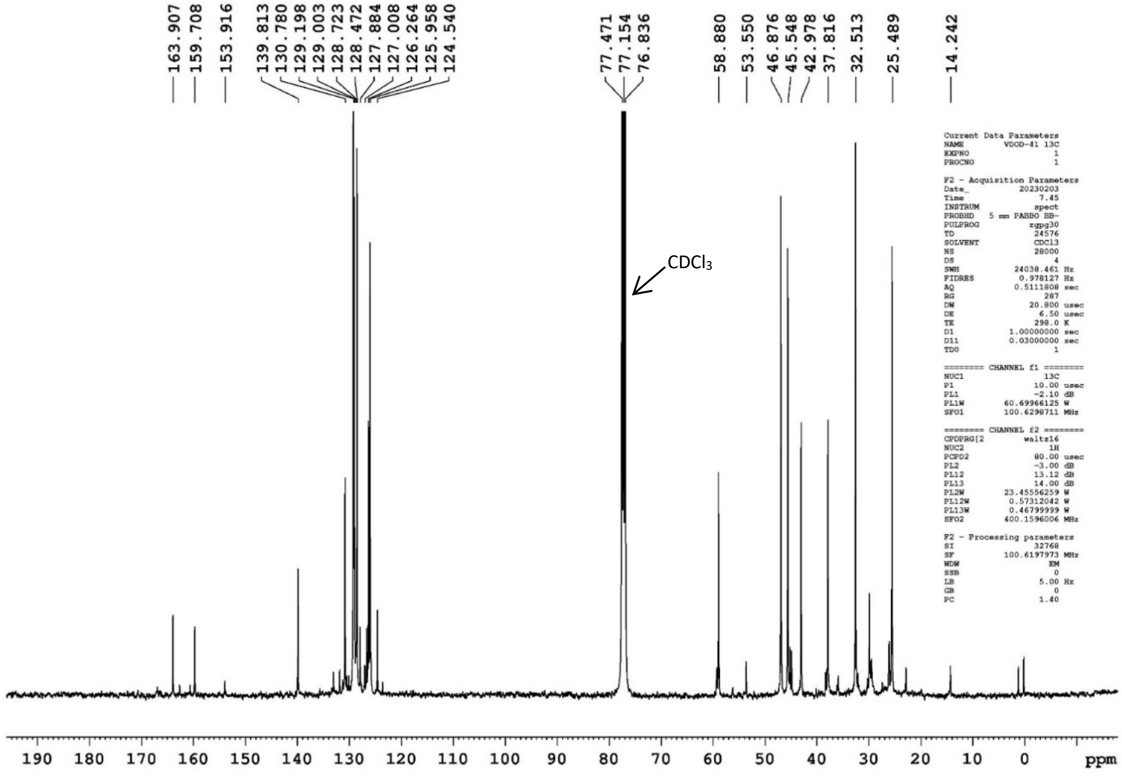

^{13}C NMR spectrum of 2-(4-((4-benzylpiperidin-1-*yl*)sulfonyl)piperidin-1-*yl*)-5-phenyl-1,3,4-oxadiazole **(P11)**

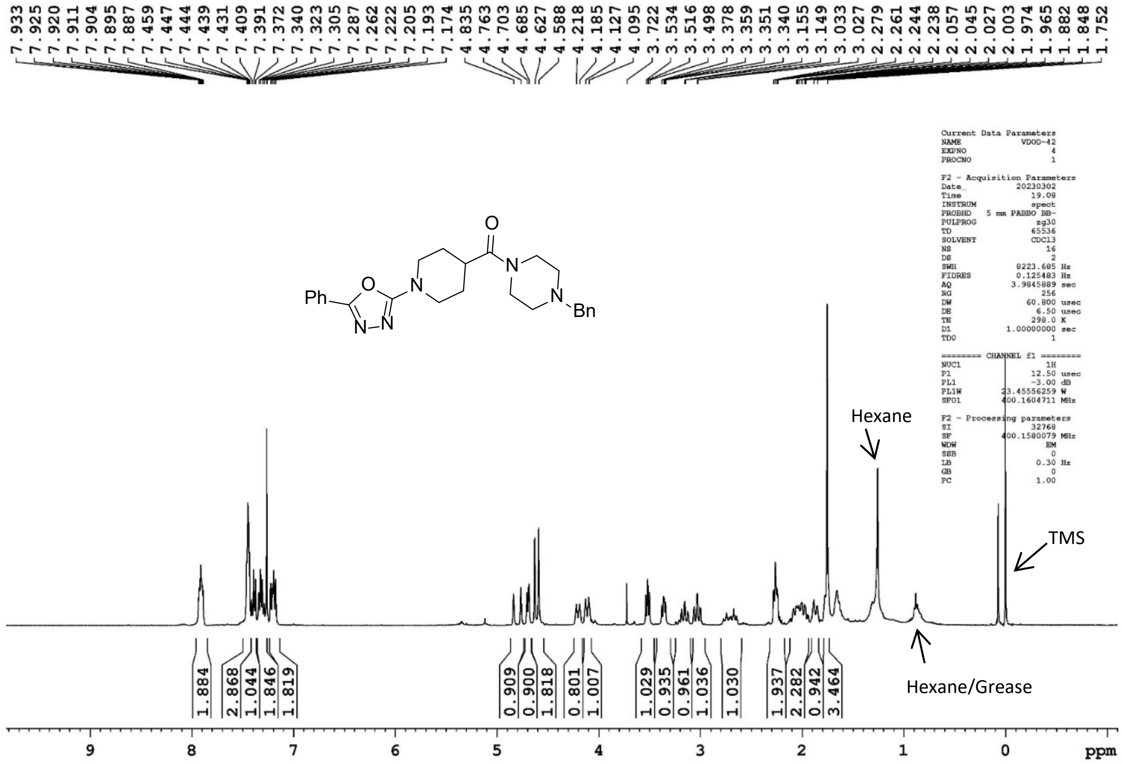

1H NMR spectrum of (4-benzylpiperazin-1-*yl*)(1-(5-phenyl-1,3,4-oxadiazol-2-*yl*)piperidin-4-*yl*)methanone **(P12)**

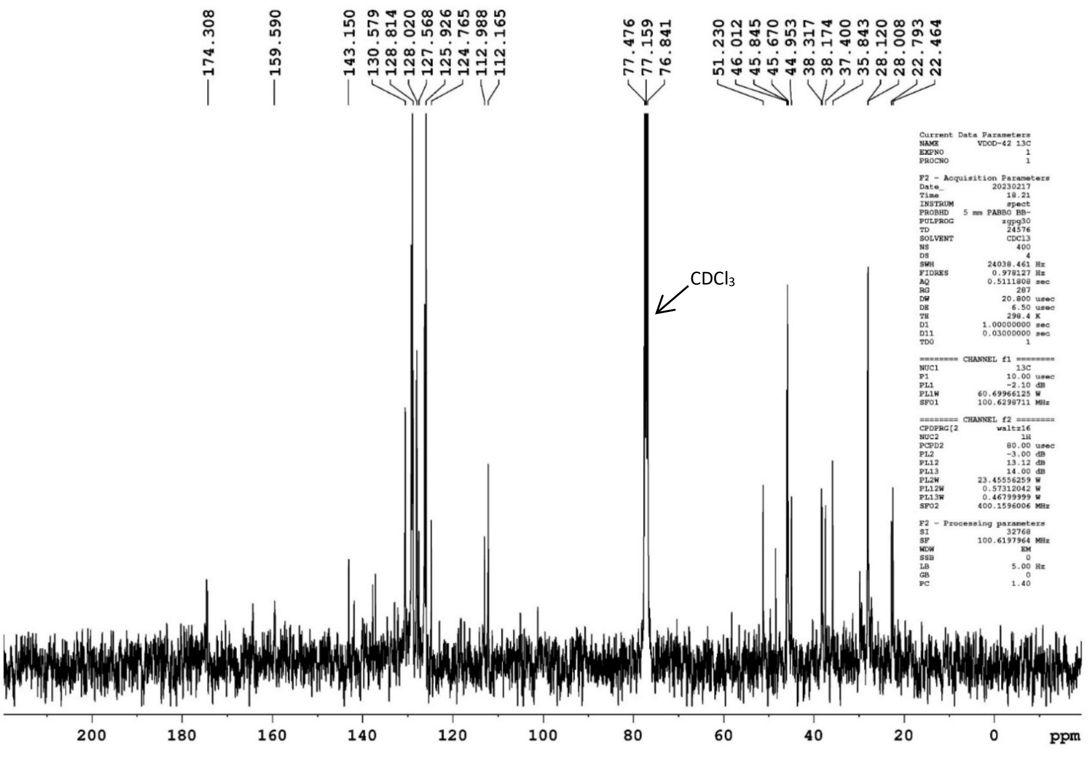

^{13}C NMR spectrum of (4-benzylpiperazin-1-*yl*)(1-(5-phenyl-1,3,4-oxadiazol-2-*yl*)piperidin-4-*yl*)methanone **(P12)**

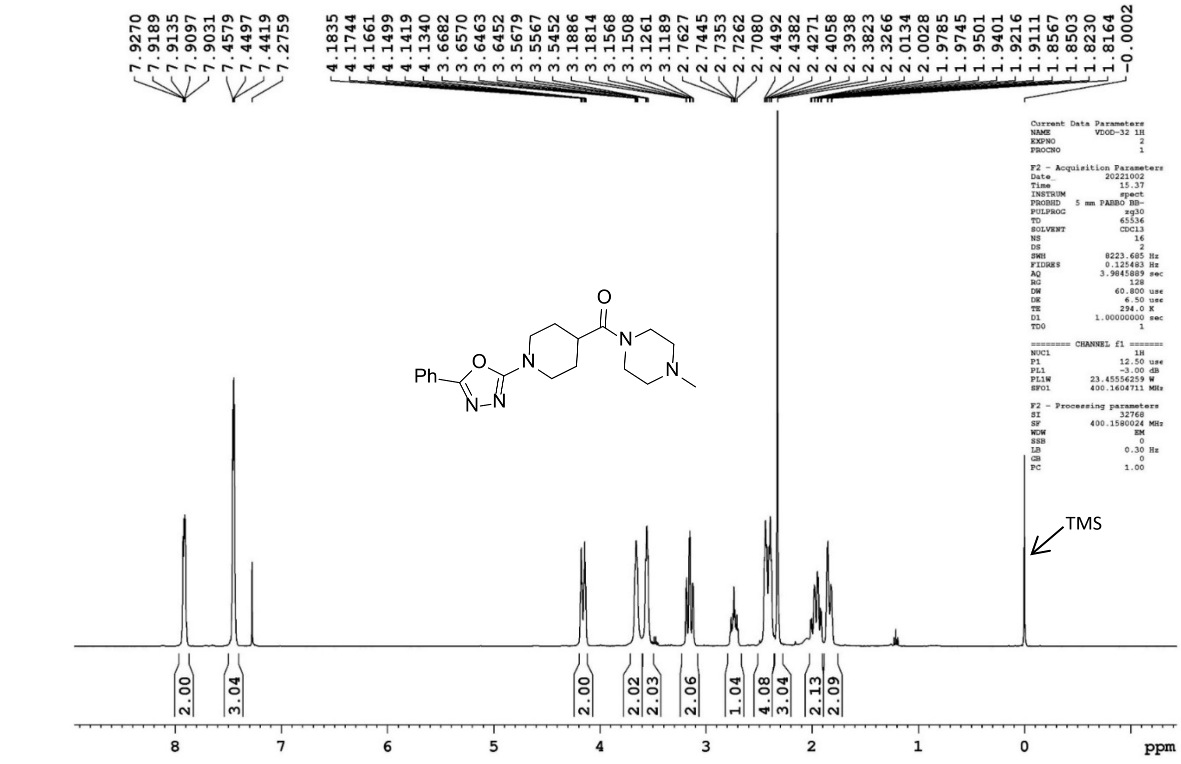

^{1}H NMR spectrum of (4-methylpiperazin-1-*yl*)(1-(5-phenyl-1,3,4-oxadiazol-2-*yl*)piperidin-4-*yl*)methanone **(P13)**

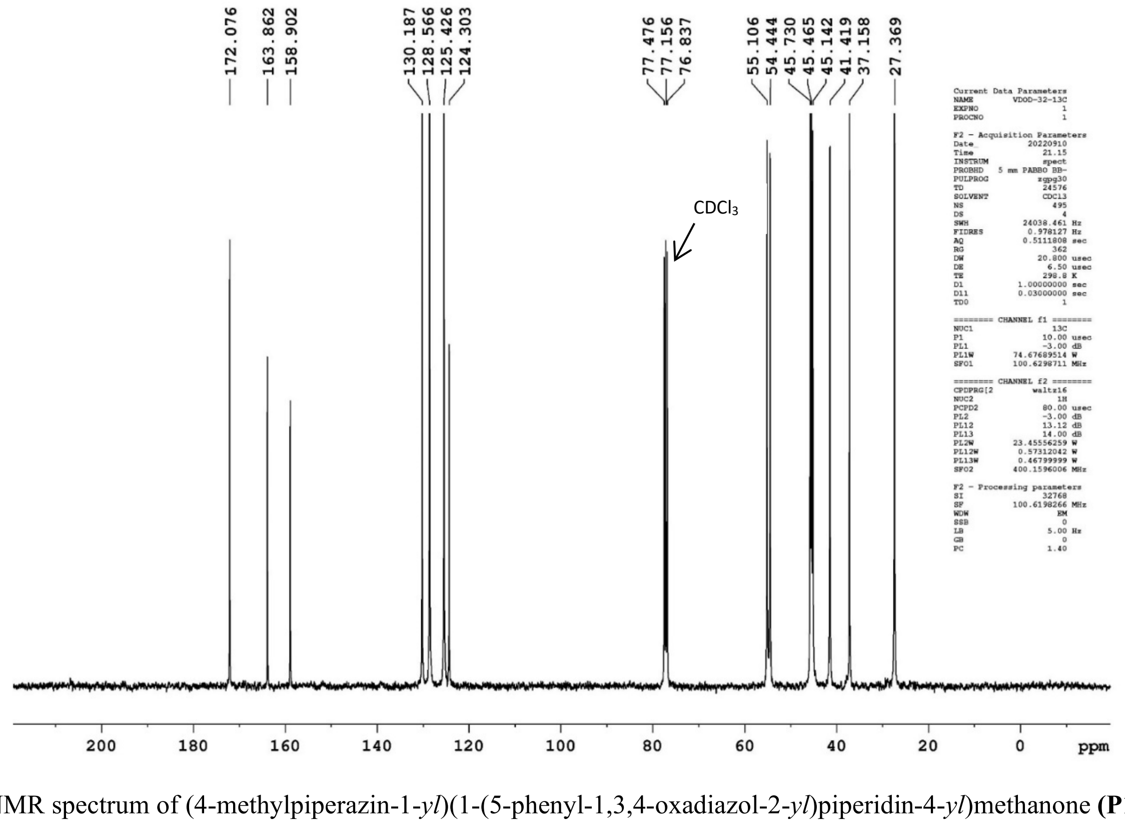

^{13}C NMR spectrum of (4-methylpiperazin-1-*yl*)(1-(5-phenyl-1,3,4-oxadiazol-2-*yl*)piperidin-4-*yl*)methanone **(P13)**

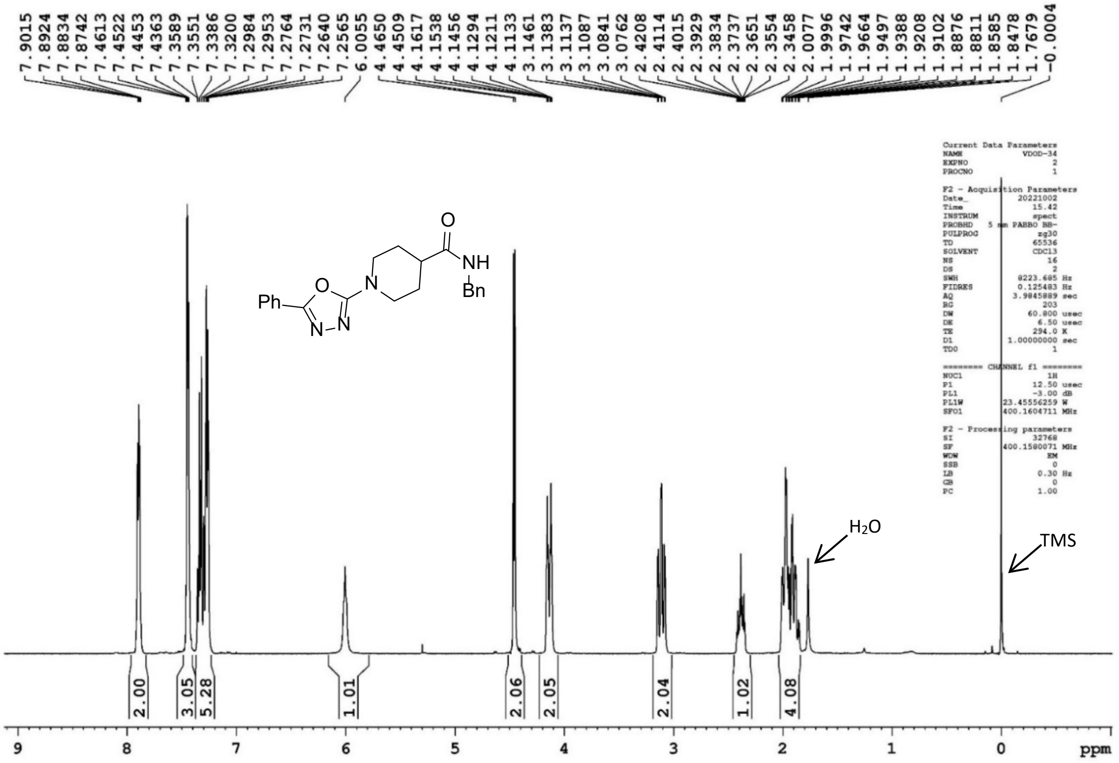

^{1}H NMR spectrum of *N*-benzyl-1-(5-phenyl-1,3,4-oxadiazol-2-*yl*)piperidine-4-carboxamide **(P14)**

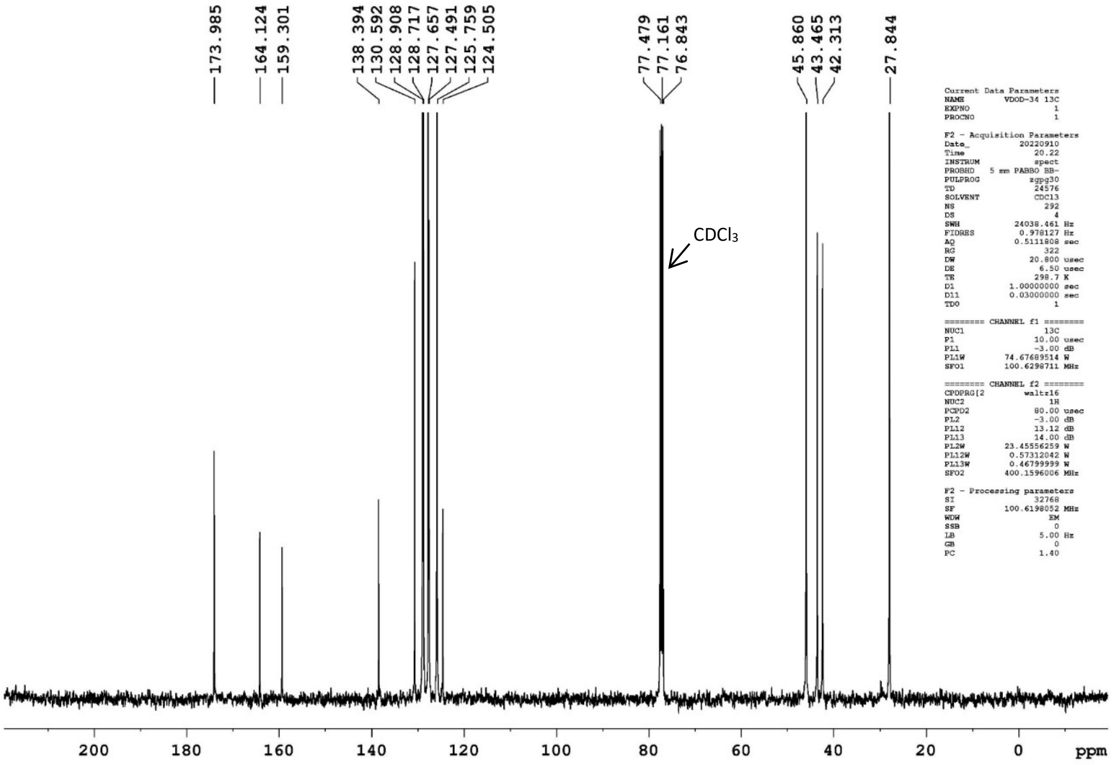

^{13}C NMR spectrum of *N*-benzyl-1-(5-phenyl-1,3,4-oxadiazol-2-*yl*)piperidine-4-carboxamide **(P14)**

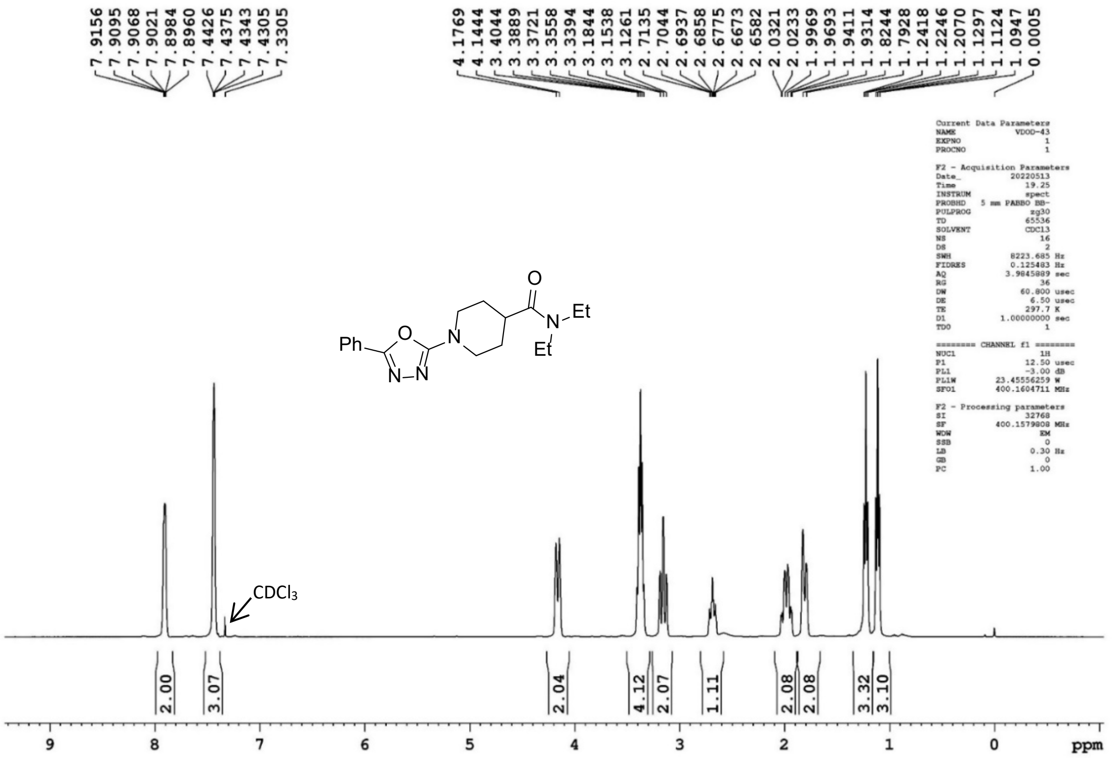

^{1}H NMR spectrum of *N,N*-diethyl-1-(5-phenyl-1,3,4-oxadiazol-2-*yl*)piperidine-4-carboxamide **(P15)**

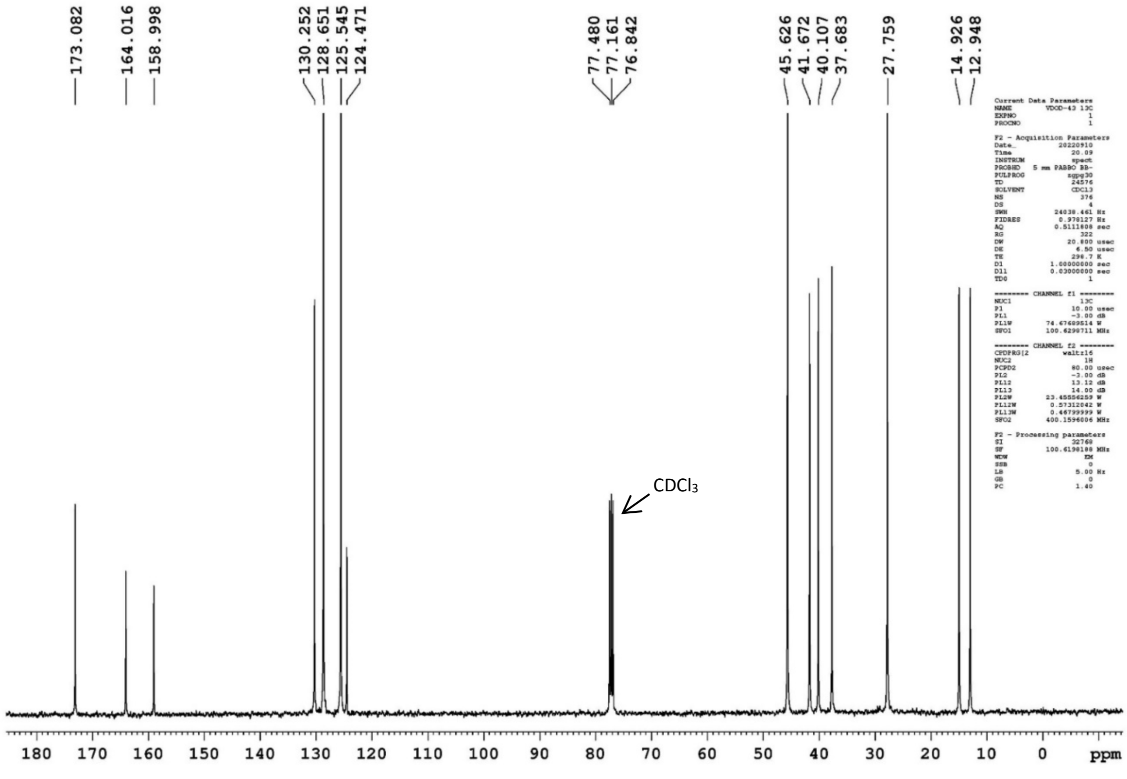

^{13}C NMR spectrum of *N,N*-diethyl-1-(5-phenyl-1,3,4-oxadiazol-2-*yl*)piperidine-4-carboxamide **(P15)**

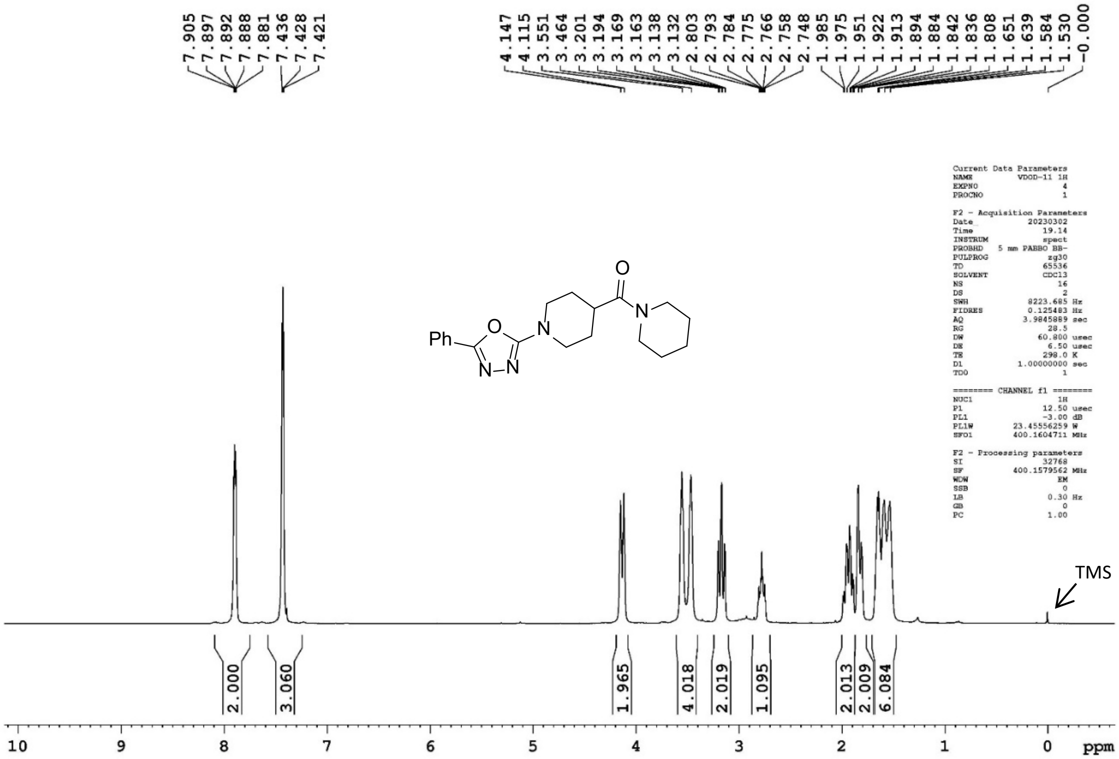

^{1}H NMR spectrum of (1-(5-phenyl-1,3,4-oxadiazol-2-*yl*)piperidin-4-*yl*)(piperidin-1-*yl*)methanone (**P16**)

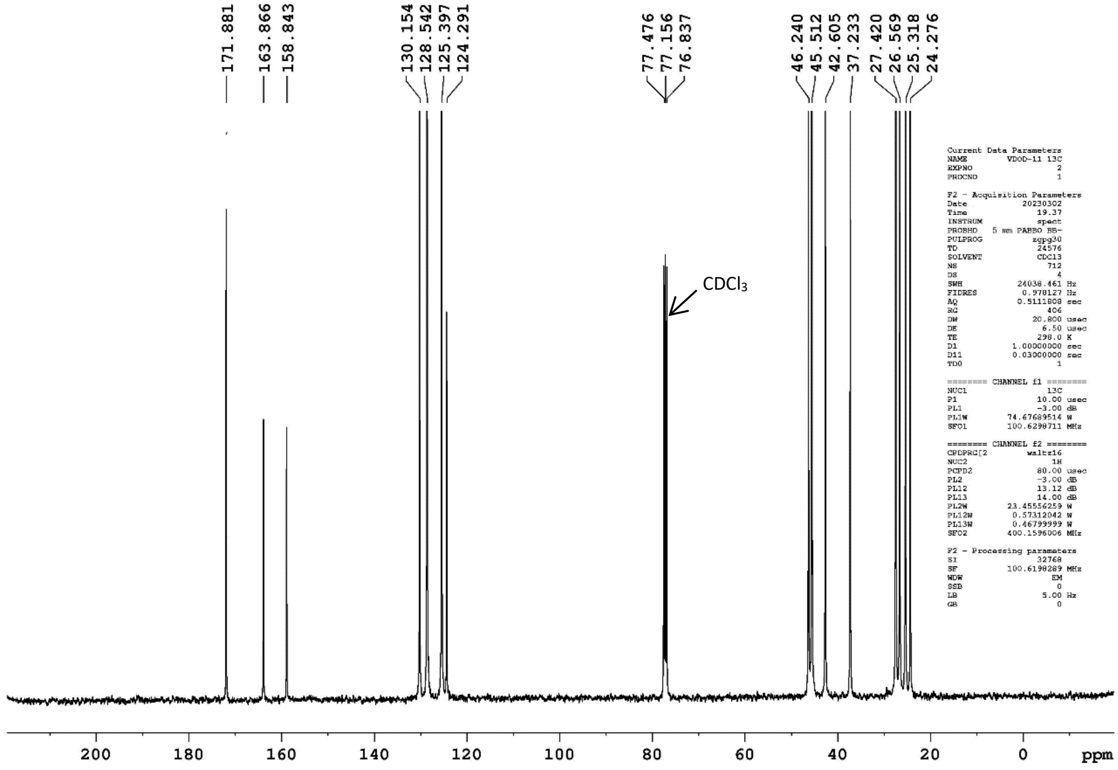

^{13}C NMR spectrum of (1-(5-phenyl-1,3,4-oxadiazol-2-*yl*)piperidin-4-*yl*)(piperidin-1-*yl*)methanone (**P16**)

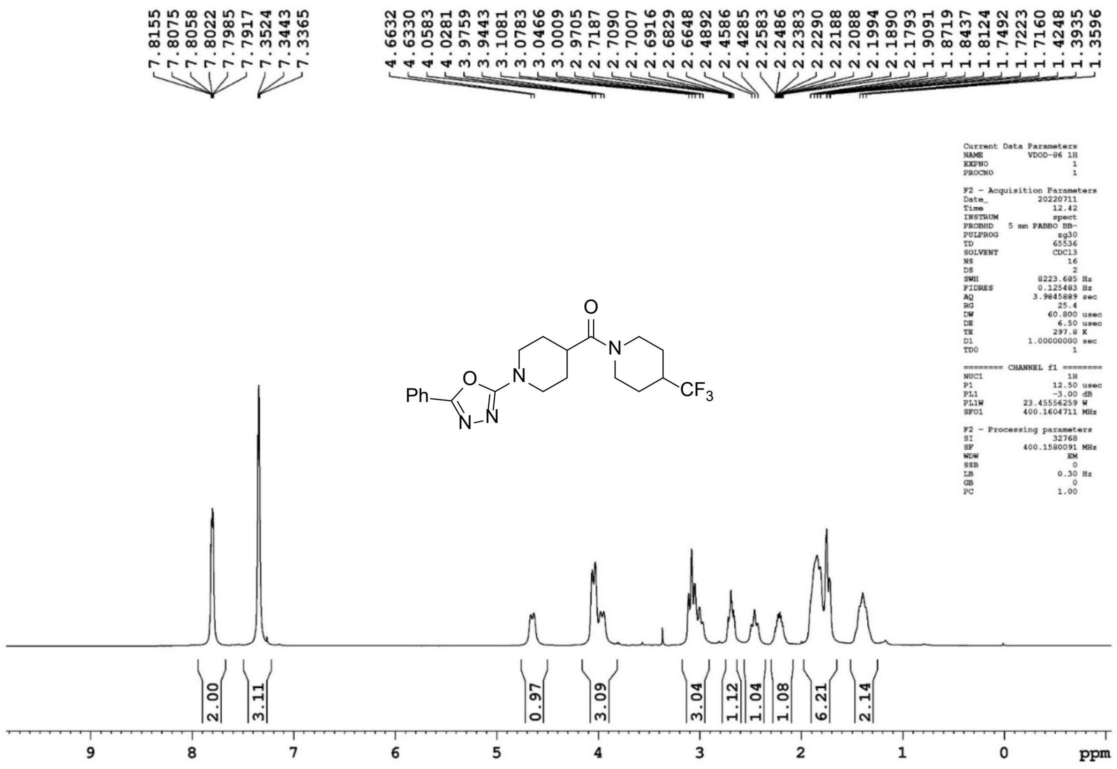

^{1}H NMR spectrum of (1-(5-phenyl-1,3,4-oxadiazol-2-*yl*)piperidin-4-*yl*)(4-(trifluoromethyl)piperidin-1-*yl*)methanone (**P17**)

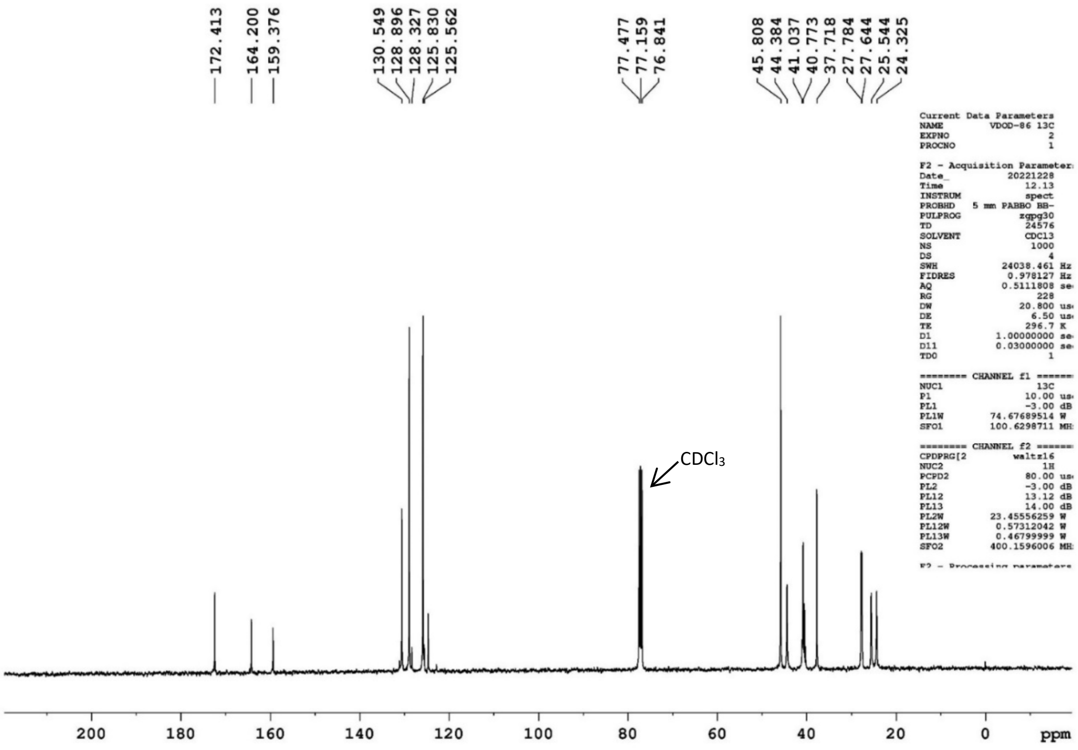

^{13}C NMR spectrum of (1-(5-phenyl-1,3,4-oxadiazol-2-*yl*)piperidin-4-*yl*)(4-(trifluoromethyl)piperidin-1-*yl*)methanone (**P17**)

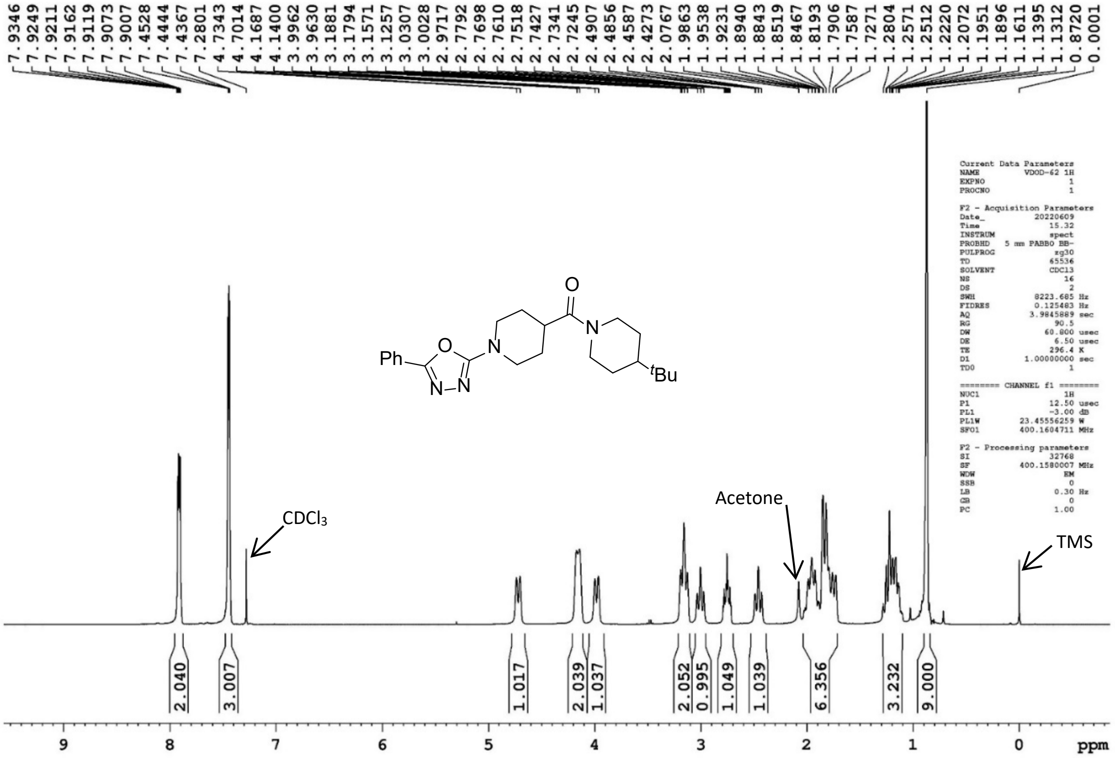

^{1}H NMR spectrum of (4-(*tert*-butyl)piperidin-1-*yl*)(1-(5-phenyl-1,3,4-oxadiazol-2-*yl*)piperidin-4-*yl*)methanone **(P18)**

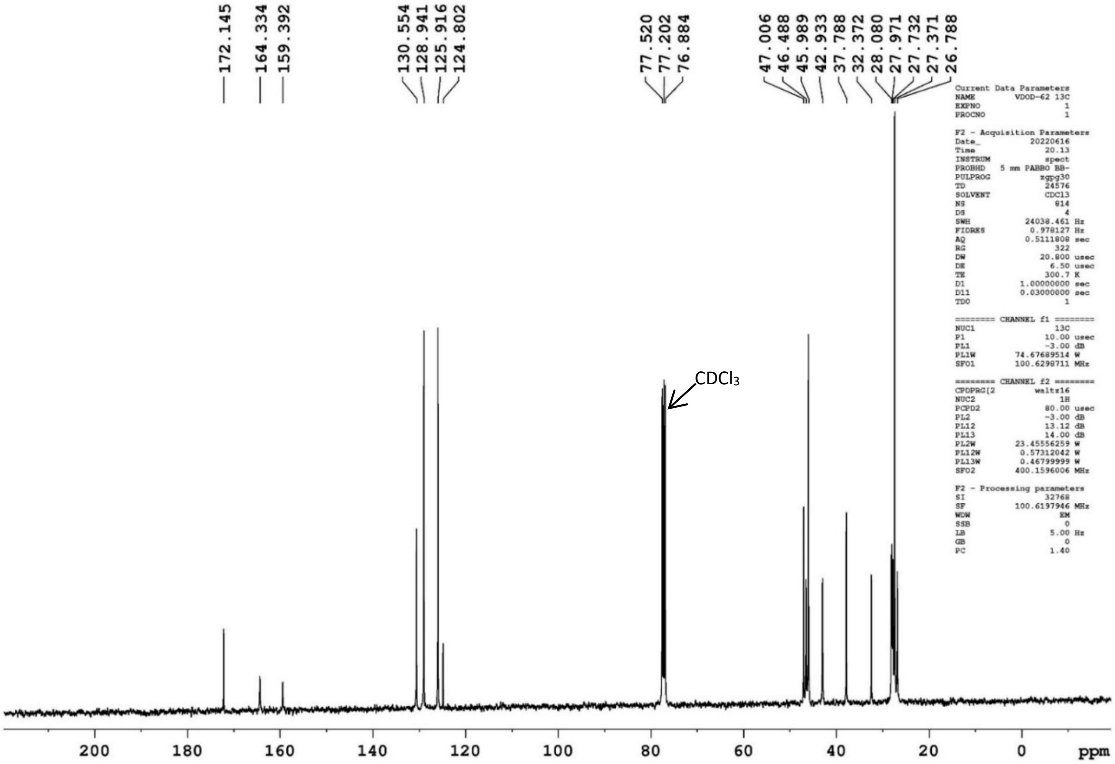

^{13}C NMR spectrum of (4-(*tert*-butyl)piperidin-1-*yl*)(1-(5-phenyl-1,3,4-oxadiazol-2-*yl*)piperidin-4-*yl*)methanone **(P18)**

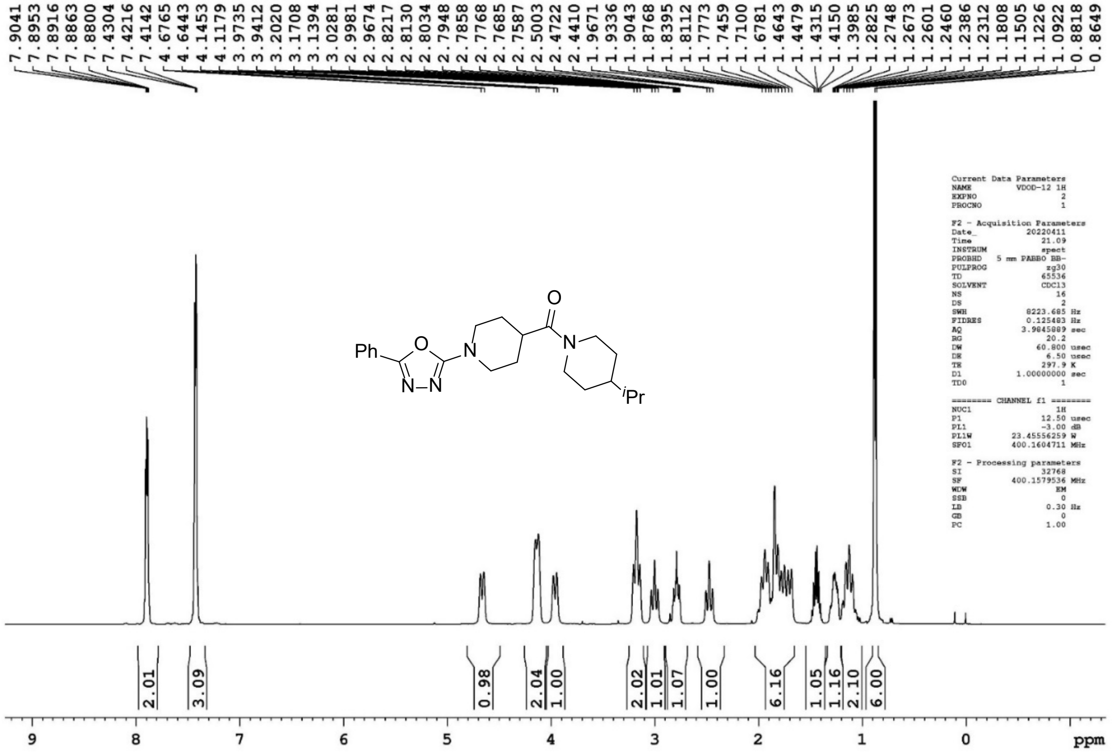

^{1}H NMR spectrum of (4-isopropylpiperidin-1-*yl*)(1-(5-phenyl-1,3,4-oxadiazol-2-*yl*)piperidin-4-*yl*)methanone **(P19)**

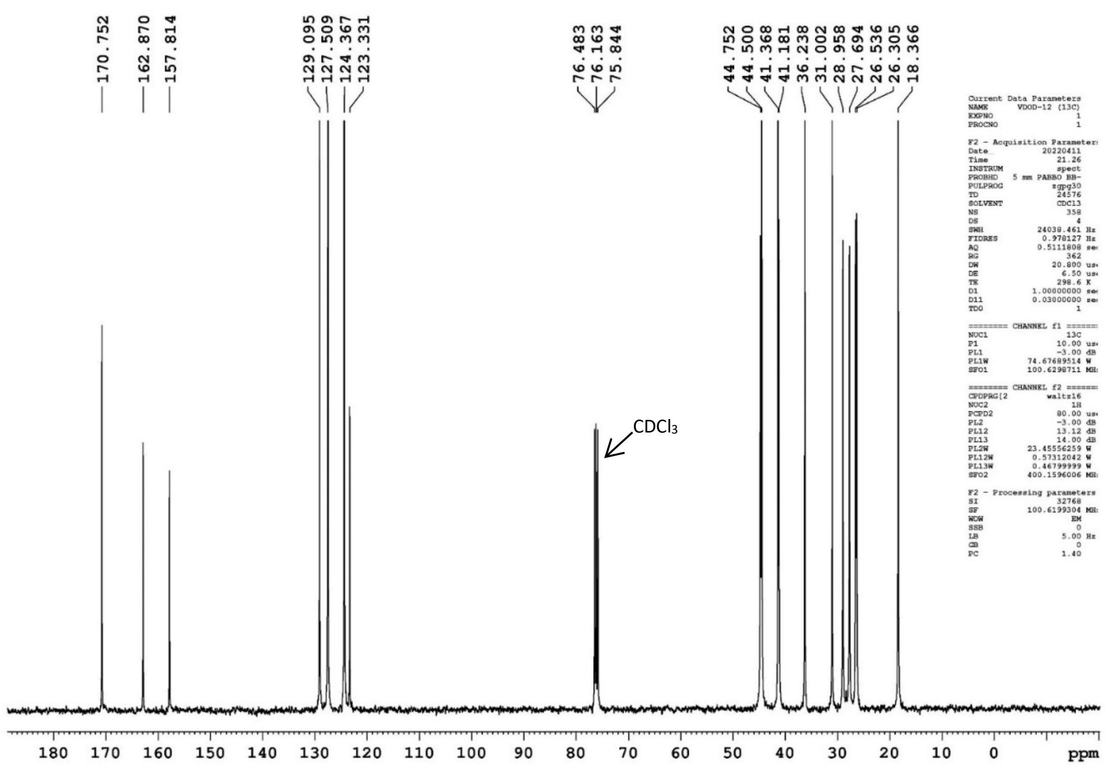

^{13}C NMR spectrum of (4-isopropylpiperidin-1-*yl*)(1-(5-phenyl-1,3,4-oxadiazol-2-*yl*)piperidin-4-*yl*)methanone **(P19)**

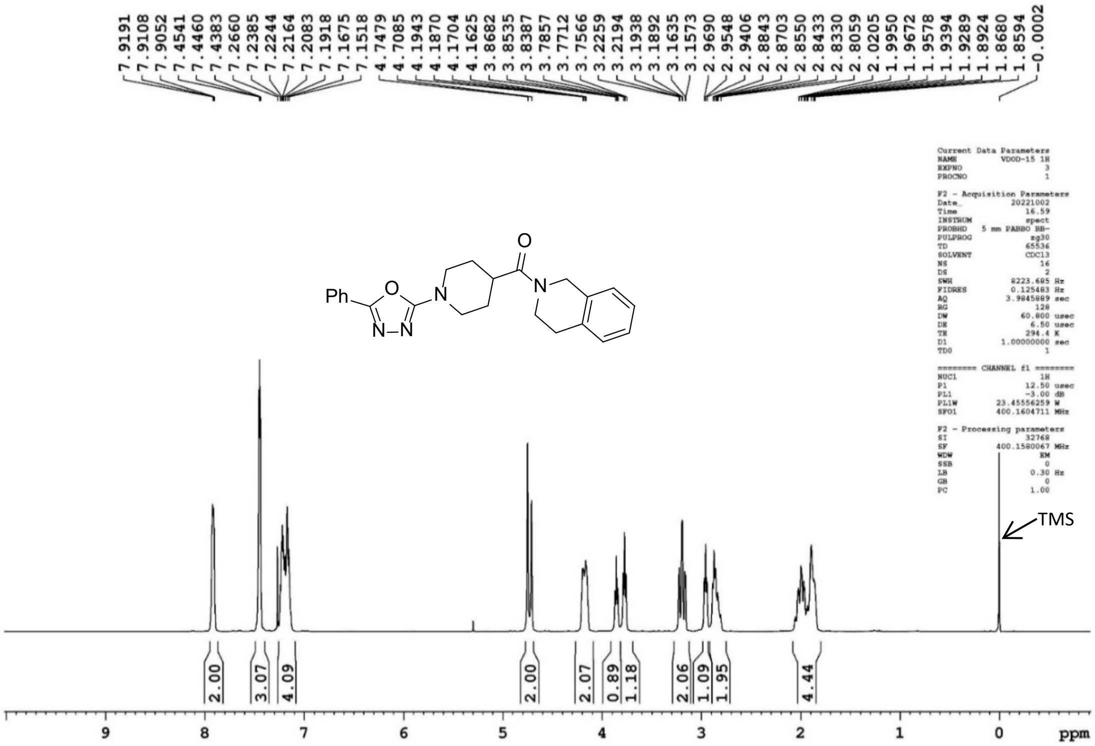

^{1}H NMR spectrum of (3,4-dihydroisoquinolin-2(1*H*)-*yl*)(1-(5-phenyl-1,3,4-oxadiazol-2-*yl*)piperidin-4-*yl*)methanone **(P20)**

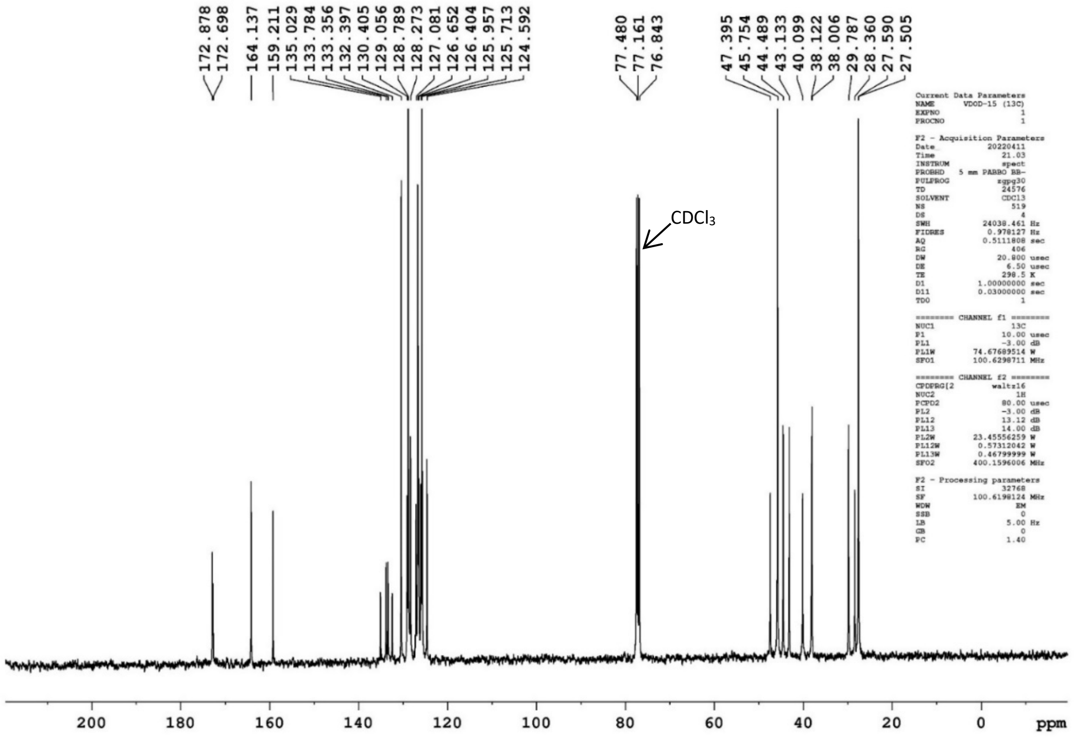

^{13}C NMR spectrum of (3,4-dihydroisoquinolin-2(1*H*)-*yl*)(1-(5-phenyl-1,3,4-oxadiazol-2-*yl*)piperidin-4-*yl*)methanone **(P20)**

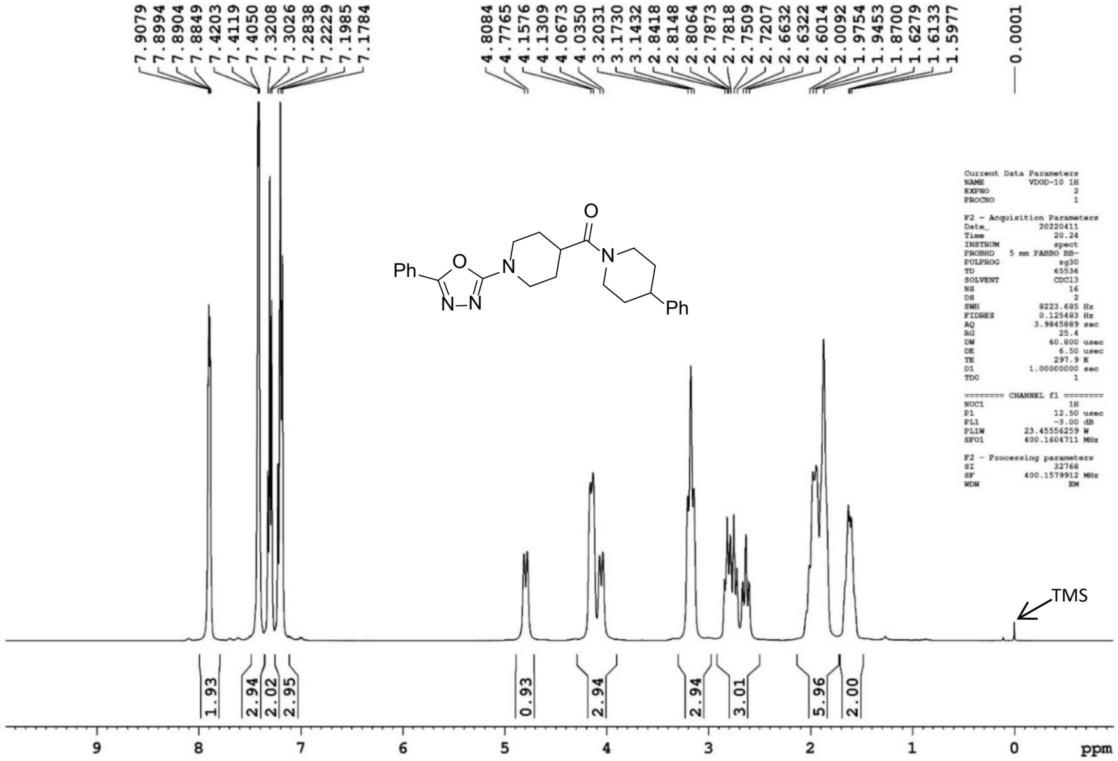

^{1}H NMR spectrum of (1-(5-phenyl-1,3,4-oxadiazol-2-*yl*)piperidin-4-*yl*)(4-phenylpiperidin-1-*yl*)methanone **(P21)**

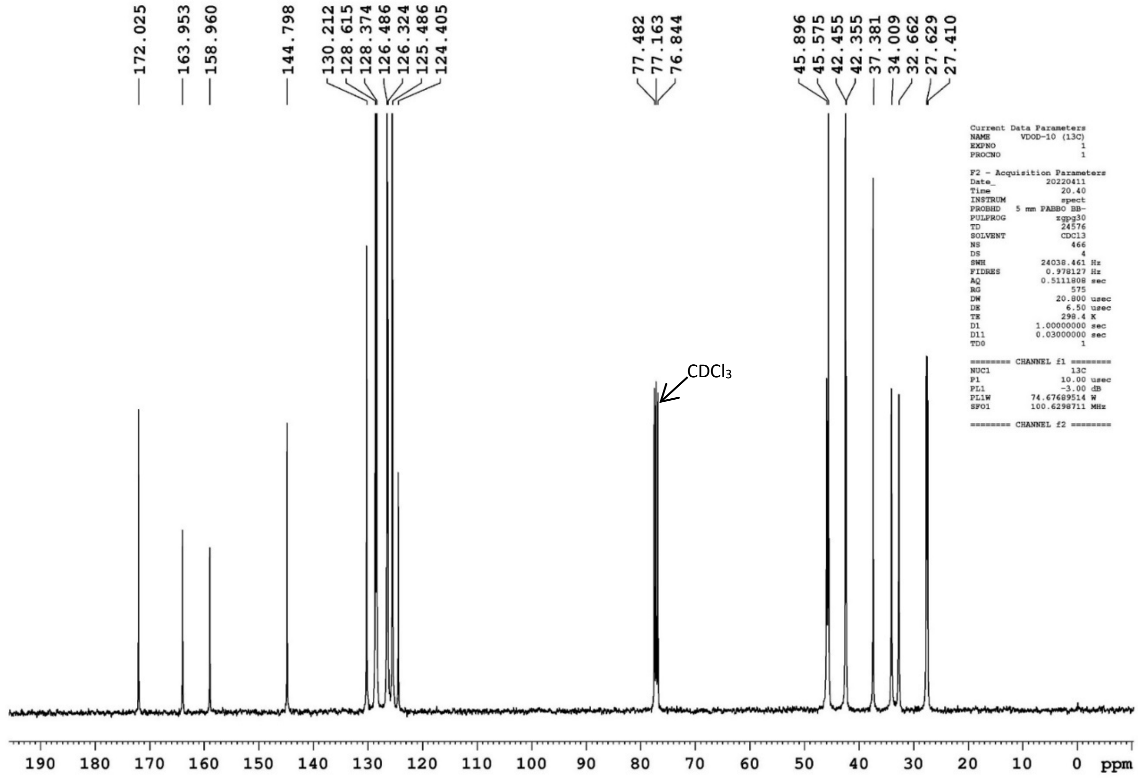

^{13}C NMR spectrum of (1-(5-phenyl-1,3,4-oxadiazol-2-*yl*)piperidin-4-*yl*)(4-phenylpiperidin-1-*yl*)methanone **(P21)**

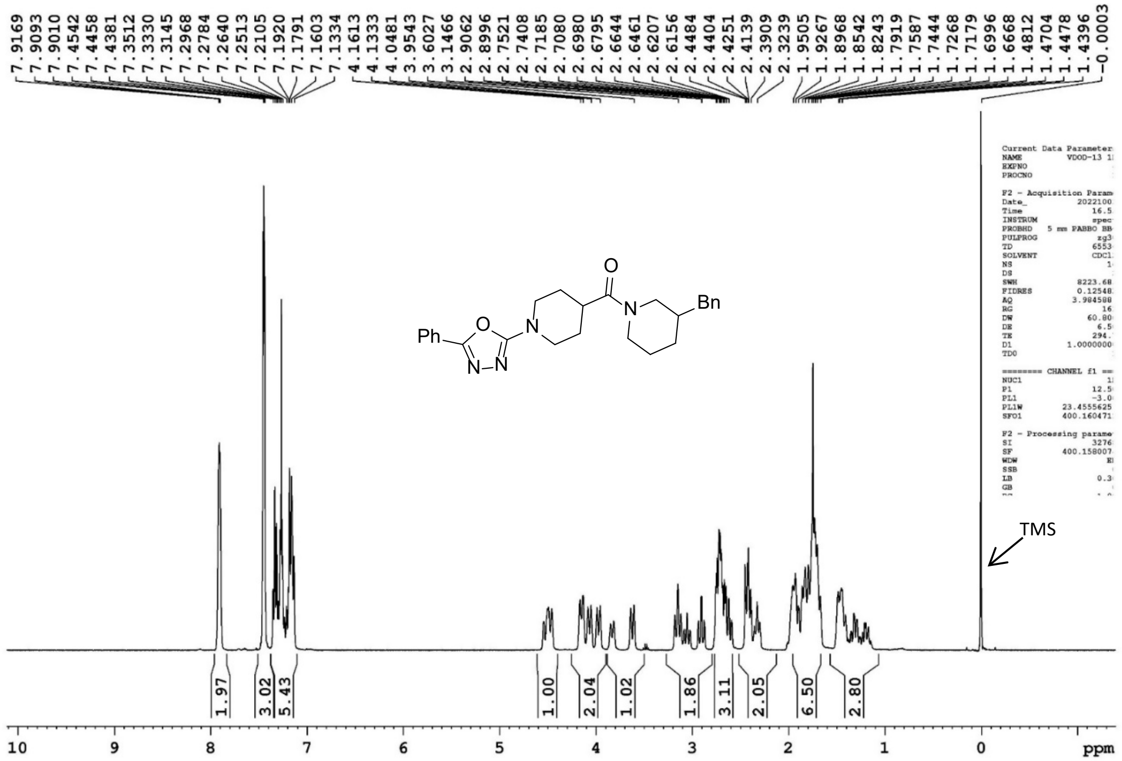

^{1}H NMR spectrum of (3-benzylpiperidin-1-*yl*)(1-(5-phenyl-1,3,4-oxadiazol-2-*yl*)piperidin-4-yl)methanone **(P22)**

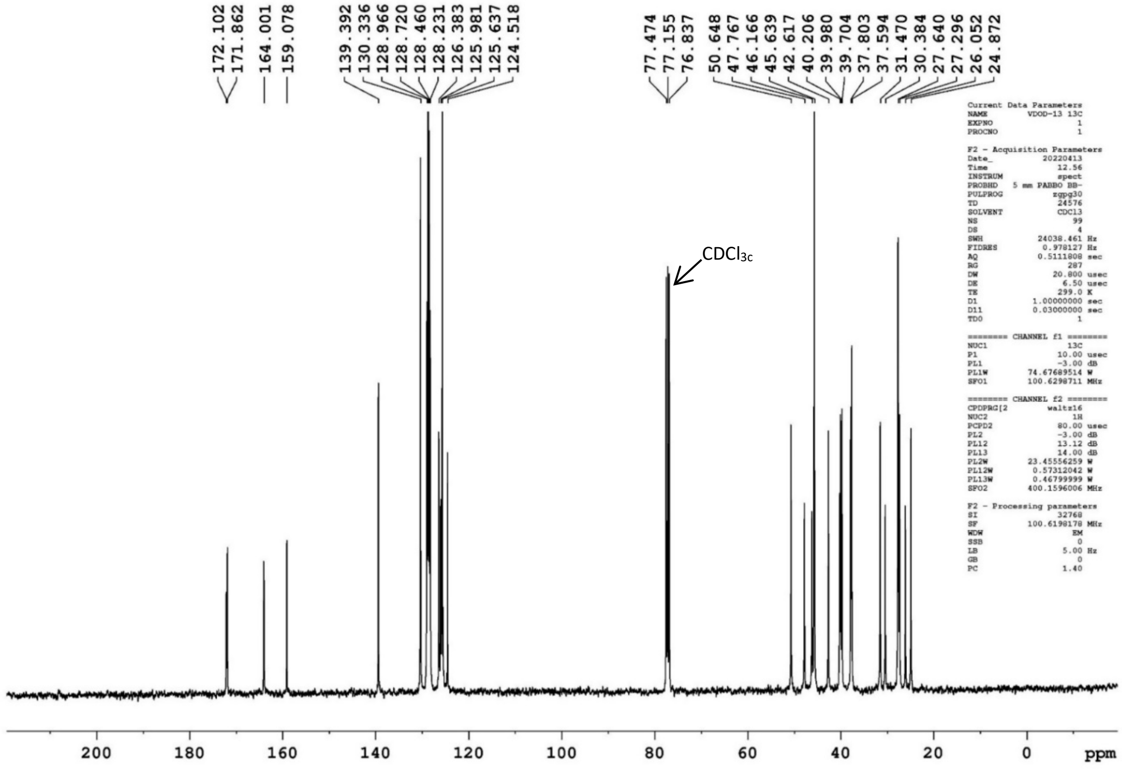

^{13}C NMR spectrum of (3-benzylpiperidin-1-*yl*)(1-(5-phenyl-1,3,4-oxadiazol-2-*yl*)piperidin-4-yl)methanone **(P22)**

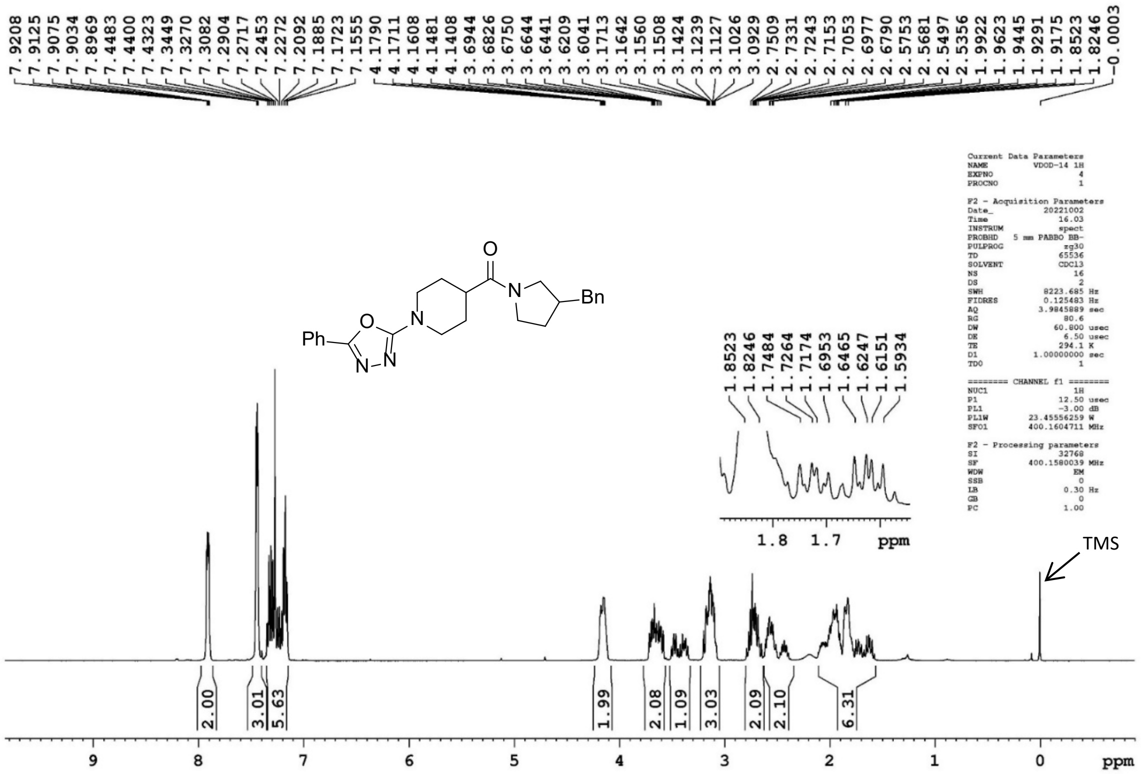

^{1}H NMR spectrum of (3-benzylpyrrolidin-1-*yl*)(1-(5-phenyl-1,3,4-oxadiazol-2-*yl*)piperidin-4-*yl*)methanone **(P23).**

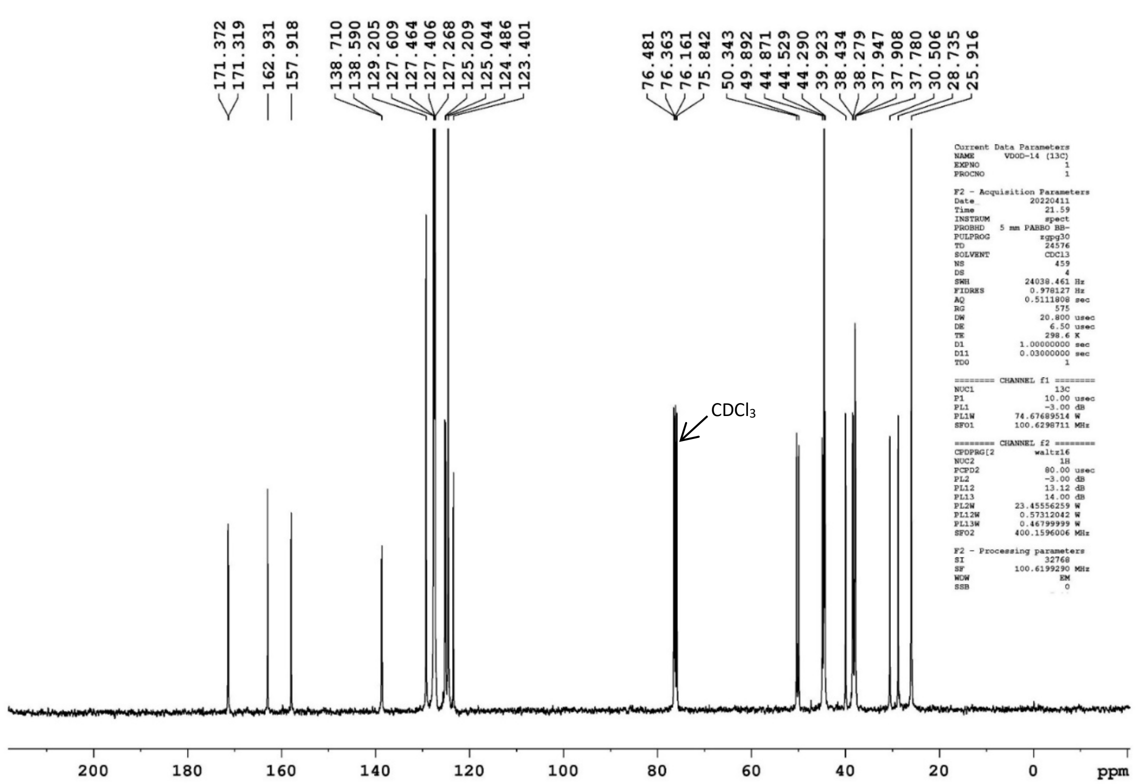

^{13}C NMR spectrum of (3-benzylpyrrolidin-1-*yl*)(1-(5-phenyl-1,3,4-oxadiazol-2-*yl*)piperidin-4-*yl*)methanone **(P23)**

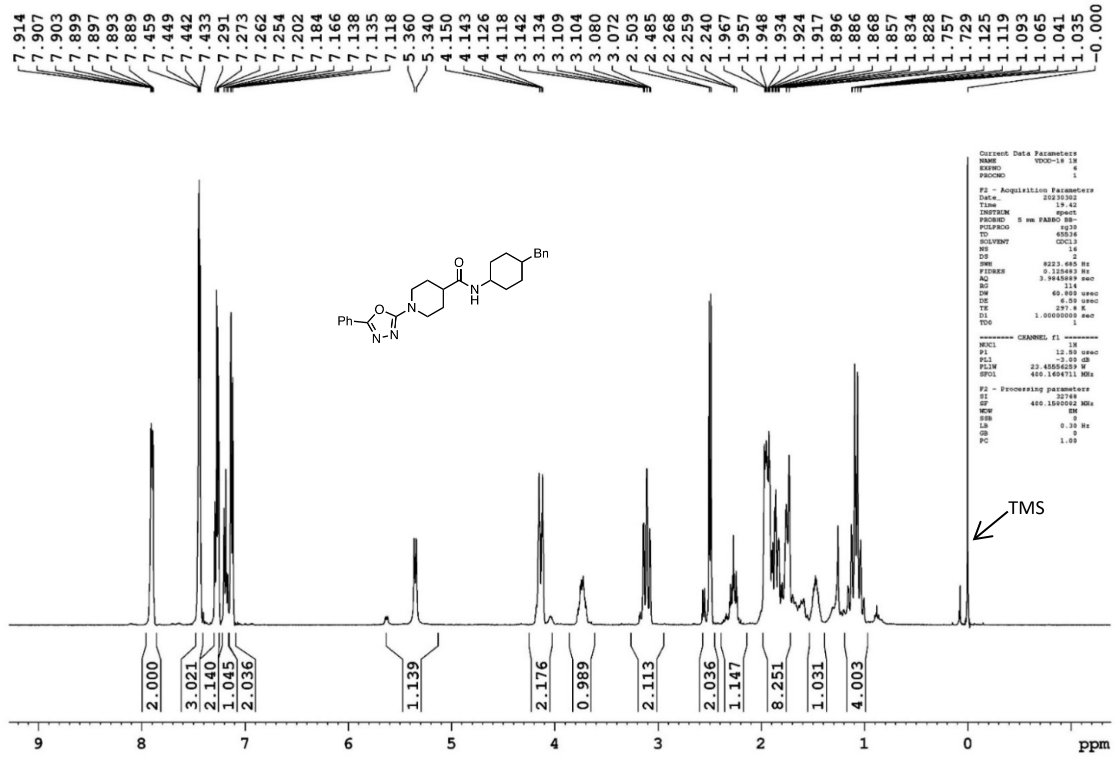

^{1}H NMR spectrum of *N*-(4-benzylcyclohexyl)-1-(5-phenyl-1,3,4-oxadiazol-2-*yl*)piperidine-4-carboxamide **(P24).**

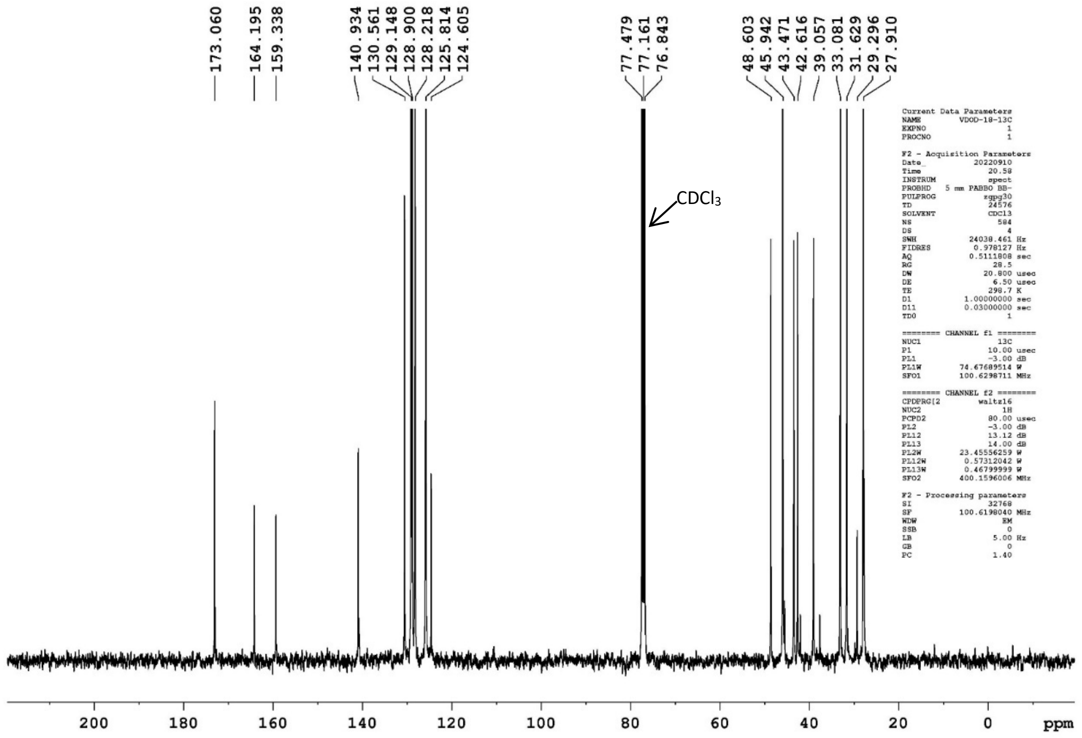

^{13}C NMR spectrum of *N*-(4-benzylcyclohexyl)-1-(5-phenyl-1,3,4-oxadiazol-2-*yl*)piperidine-4-carboxamide **(P24).**

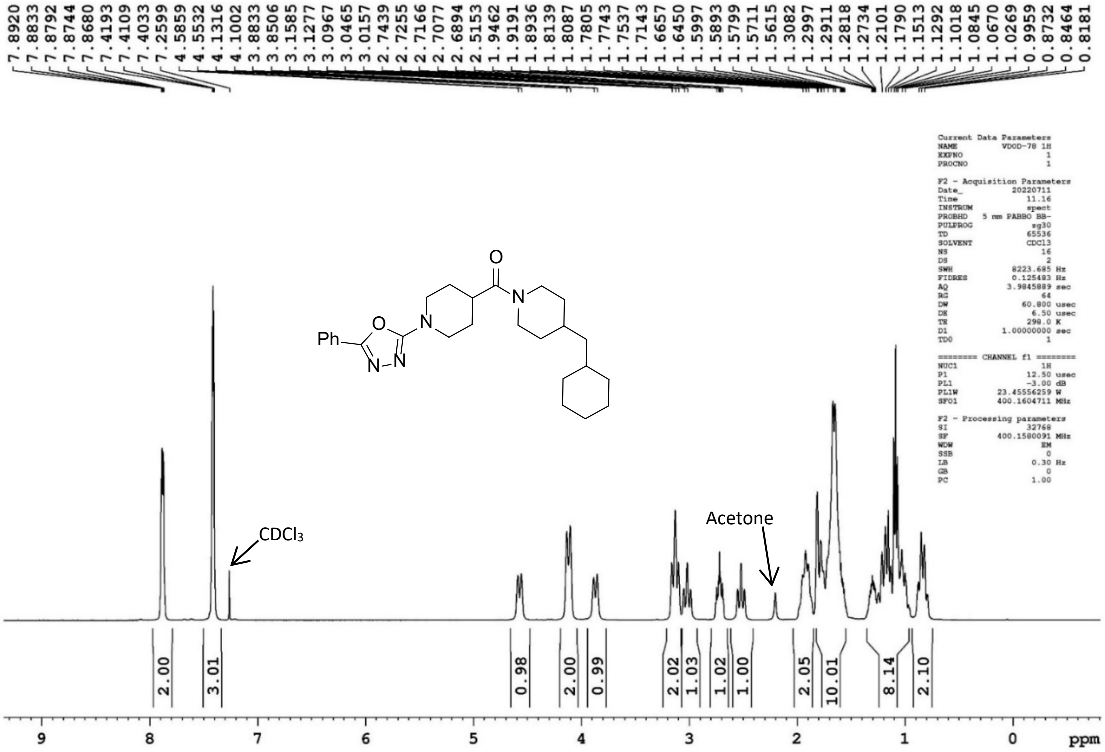

^{1}H NMR spectrum of (4-(cyclohexylmethyl)piperidin-1-yl)(1-(5-phenyl-1,3,4-oxadiazol-2-yl)piperidin-4-yl)methanone **(P25)**

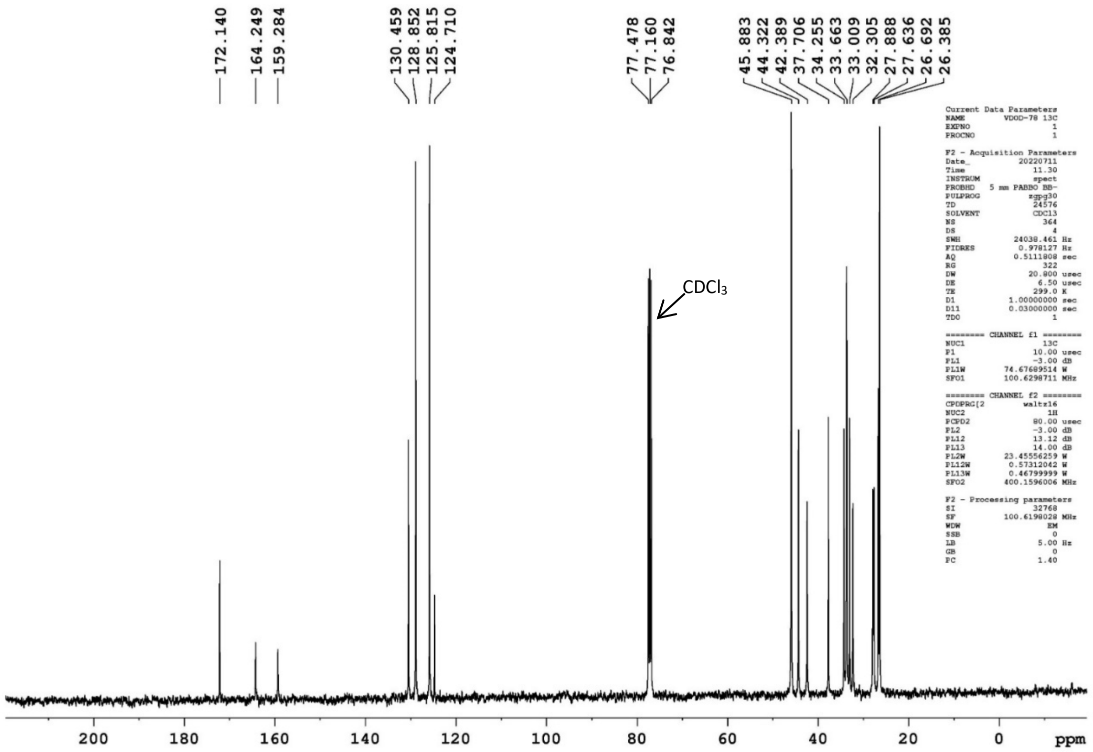

^{13}C NMR spectrum of (4-(cyclohexylmethyl)piperidin-1-*yl*)(1-(5-phenyl-1,3,4-oxadiazol-2-*yl*)piperidin-4-yl)methanone **(P25)**

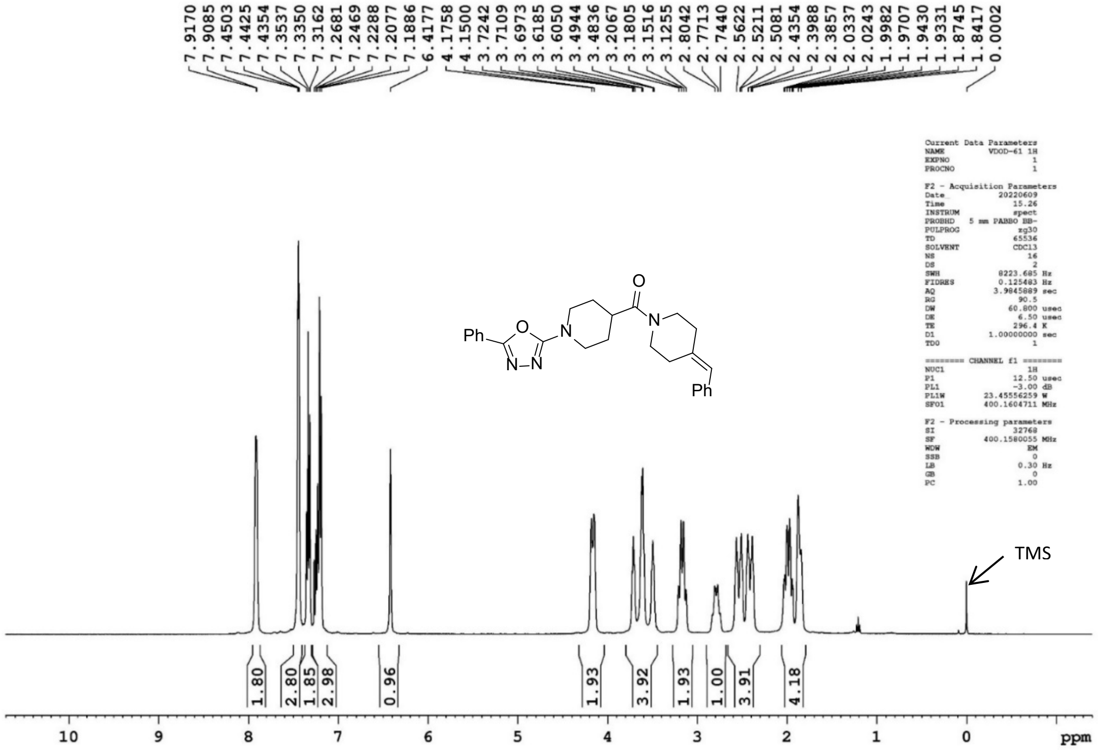

^{1}H NMR spectrum of (4-benzylidenepiperidin-1-*yl*)(1-(5-phenyl-1,3,4-oxadiazol-2-*yl*)piperidin-4-*yl*)methanone **(P26)**

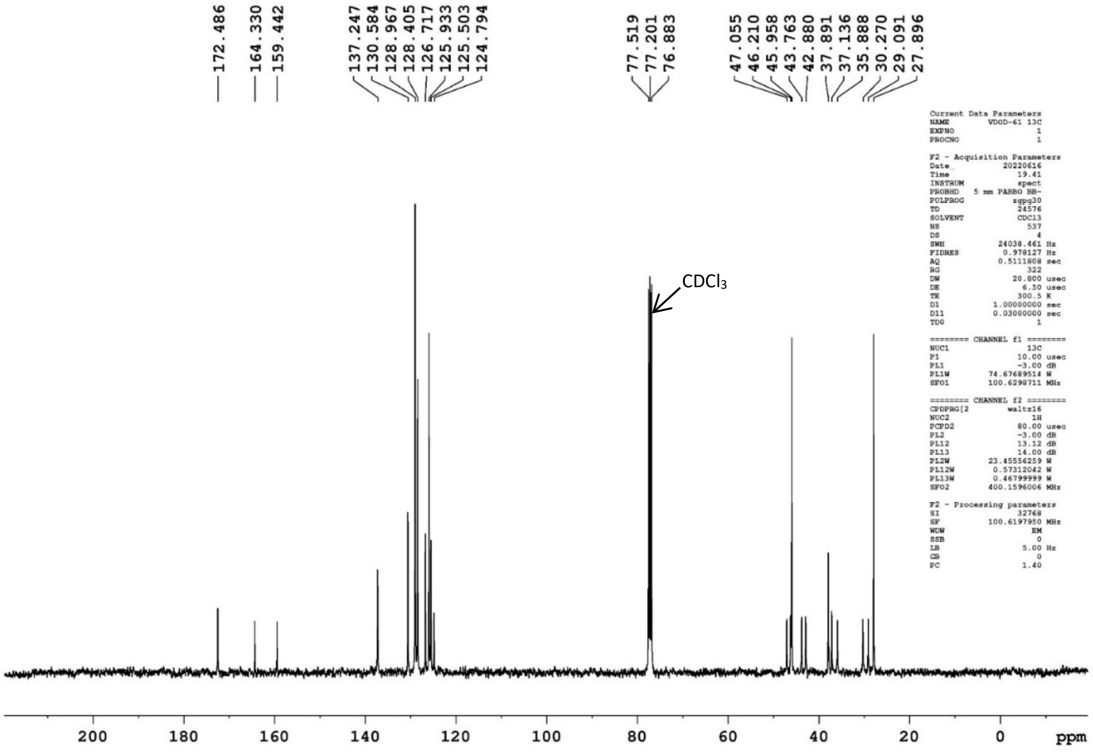

^{13}C NMR spectrum of (4-benzylidenepiperidin-1-*yl*)(1-(5-phenyl-1,3,4-oxadiazol-2-*yl*)piperidin-4-*yl*)methanone **(P26)**

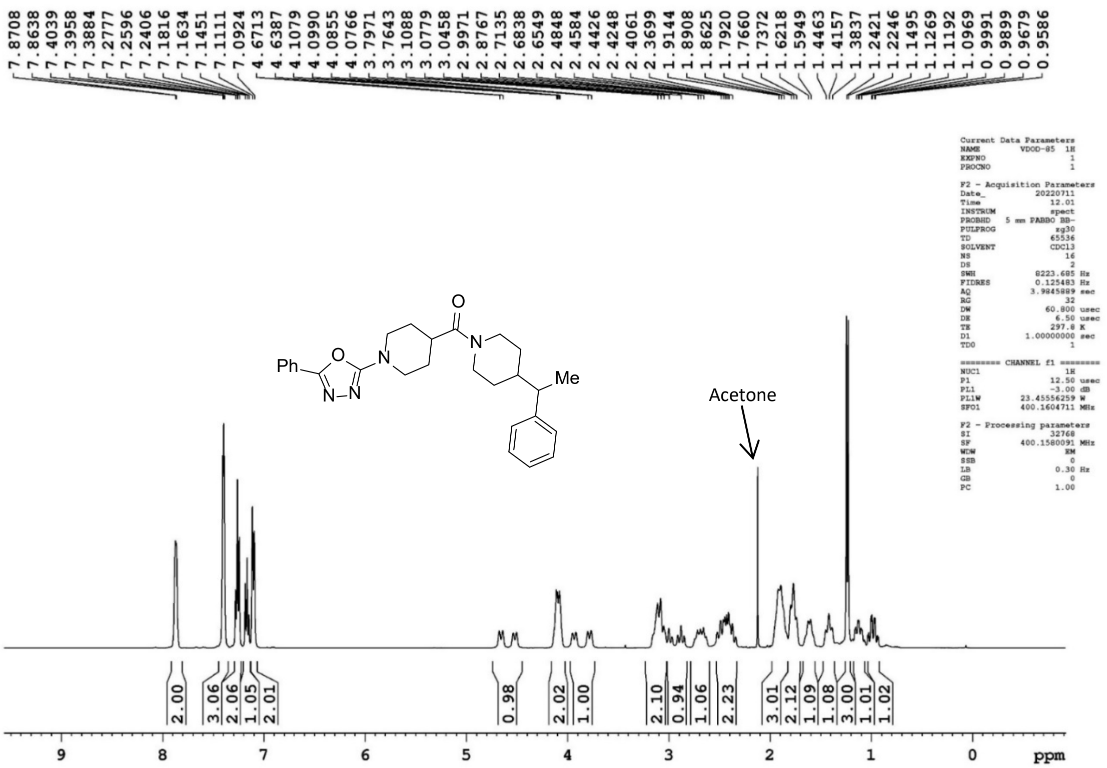

^{1}H NMR spectrum of (1-(5-Phenyl-1,3,4-oxadiazol-2-*yl*)piperidin-4-*yl*)(4-(1-phenylethyl)piperidin-1-*yl*)methanone **(P27)**

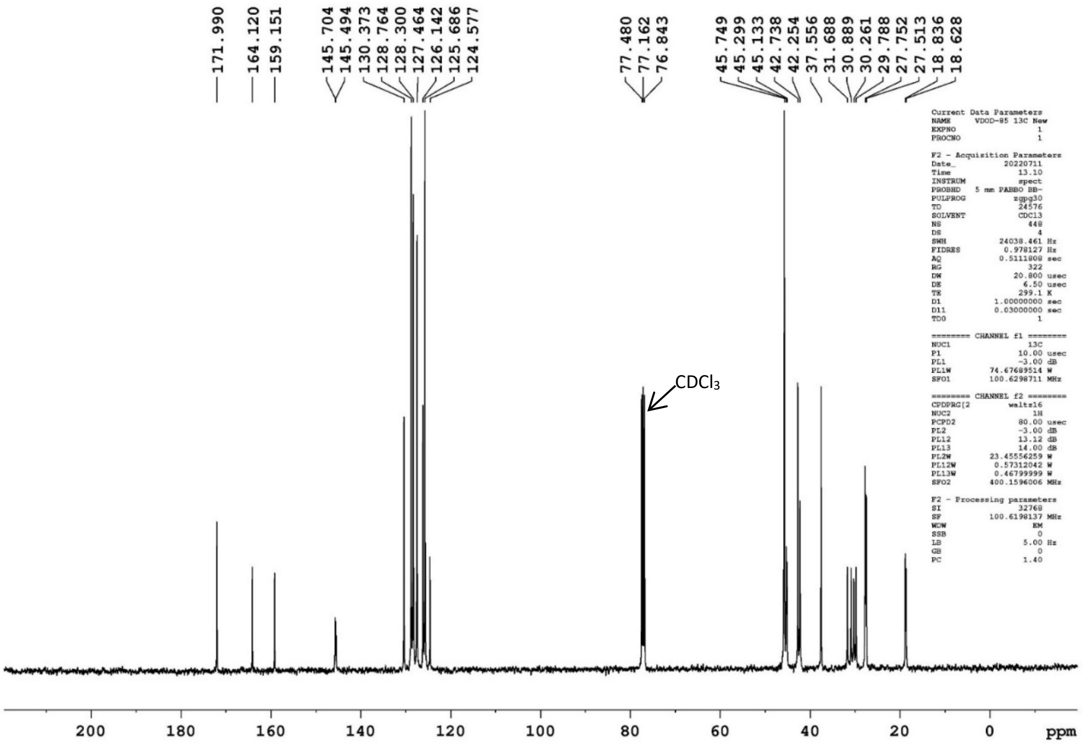

^{13}C NMR spectrum of (1-(5-phenyl-1,3,4-oxadiazol-2-*yl*)piperidin-4-*yl*)(4-(1-phenylethyl)piperidin-1-*yl*)methanone **(P27)**

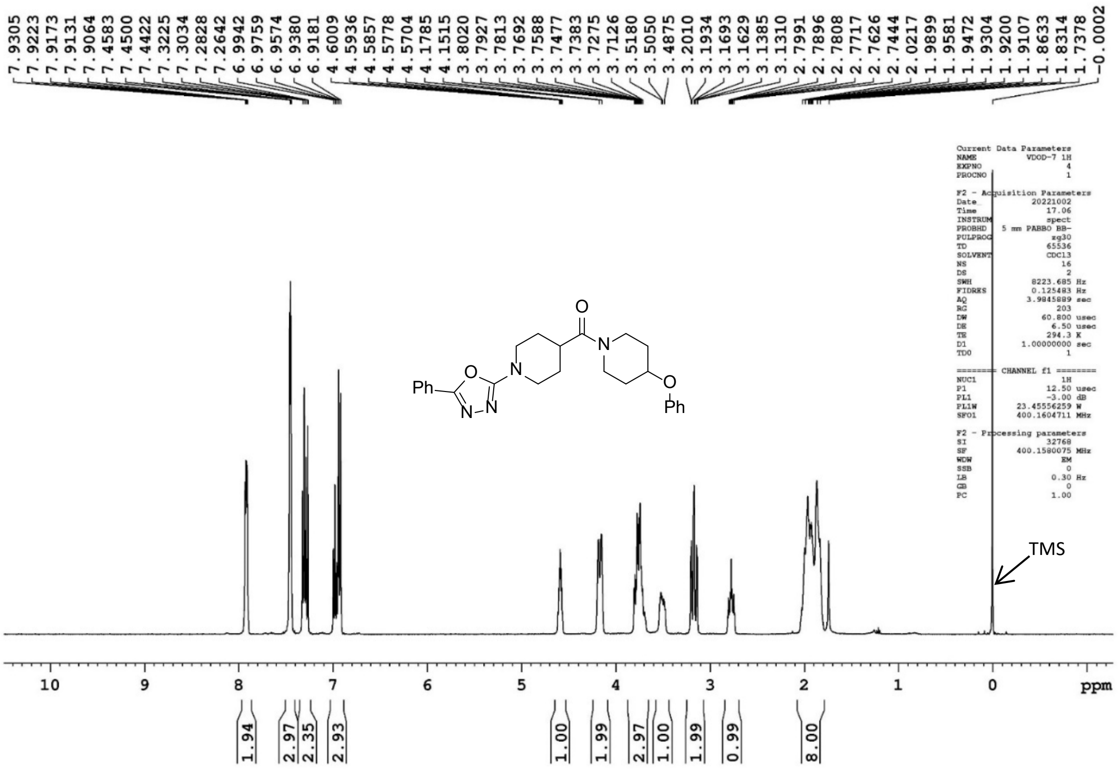

^{1}H NMR spectrum of of (4-phenoxypiperidin-1-*yl*)(1-(5-phenyl-1,3,4-oxadiazol-2-*yl*)piperidin-4-*yl*)methanone **(P28)**

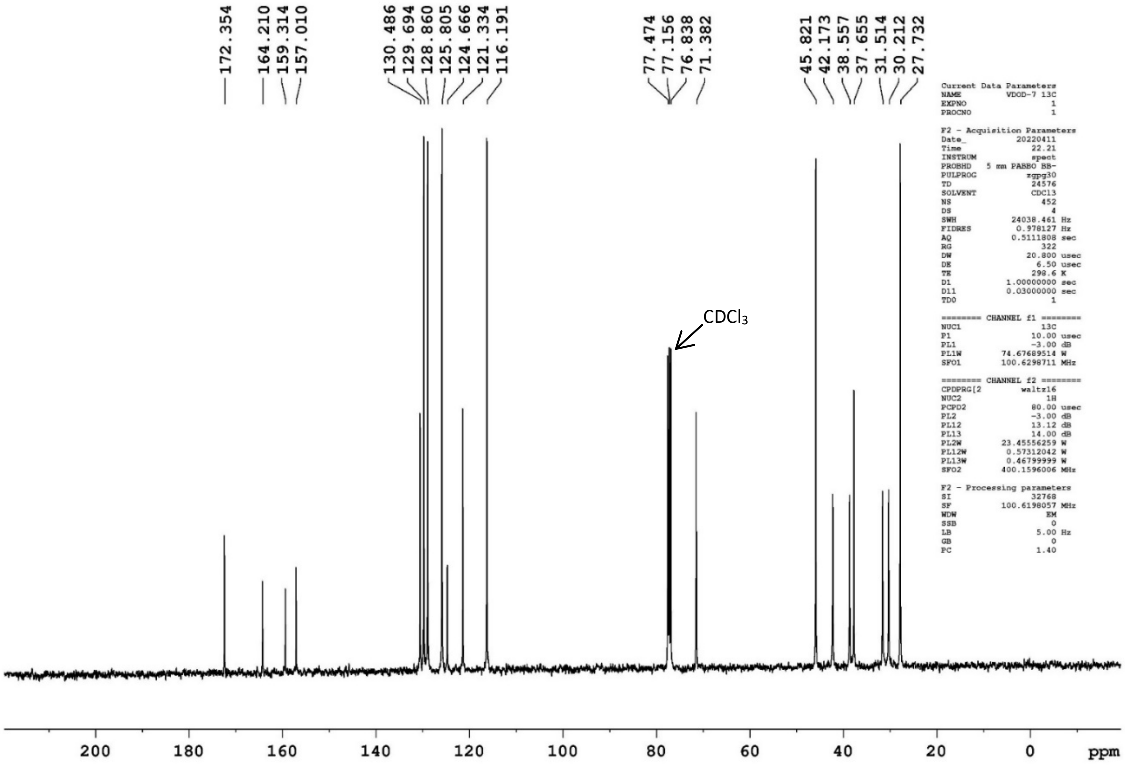

^{13}C NMR spectrum of (4-Phenoxypiperidin-1-*yl*)(1-(5-phenyl-1,3,4-oxadiazol-2-*yl*)piperidin-4-*yl*)methanone **(P28)**

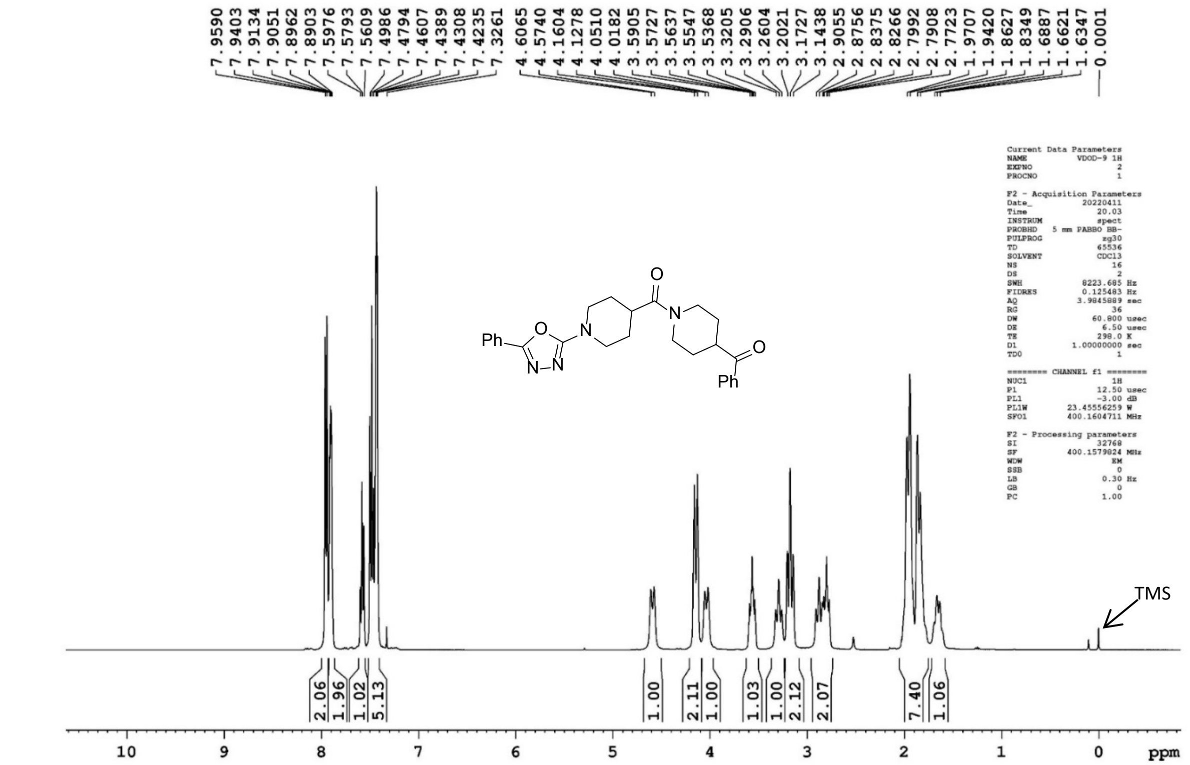

^{1}H NMR spectrum of (4-benzoylpiperidin-1-*yl*)(1-(5-phenyl-1,3,4-oxadiazol-2-*yl*)piperidin-4-*yl*)methanone **(P29)**

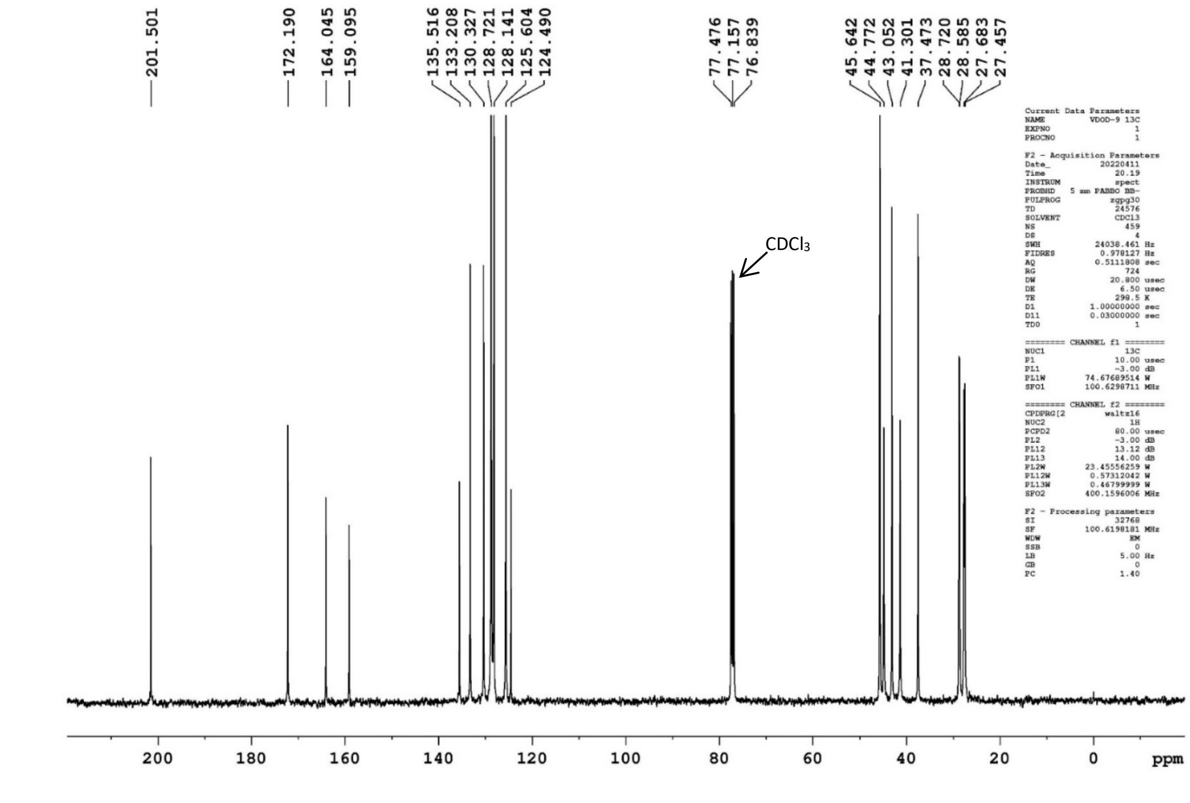

^{13}C NMR spectrum of (4-benzoylpiperidin-1-*yl*)(1-(5-phenyl-1,3,4-oxadiazol-2-*yl*)piperidin-4-*yl*)methanone **(P29)**

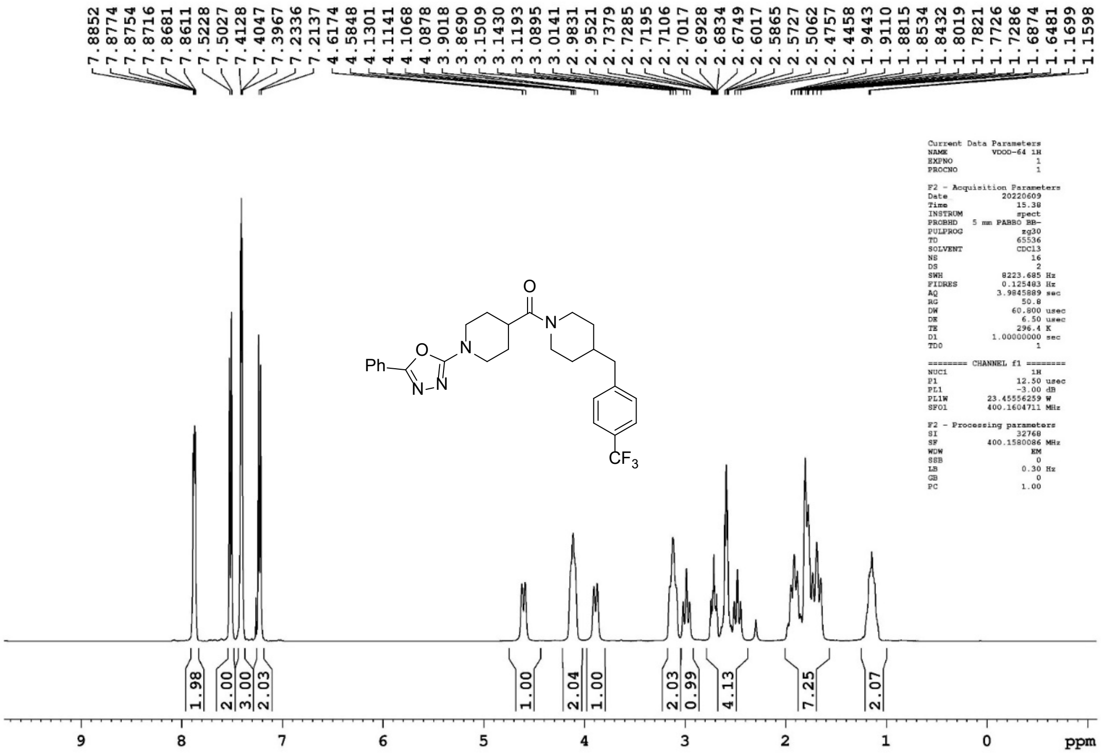

^{1}H NMR spectrum of (1-(5-phenyl-1,3,4-oxadiazol-2-*yl*)piperidin-4-*yl*)(4-(4-(trifluoromethyl)benzyl)piperidin-1-*yl*)methanone **(P30)**

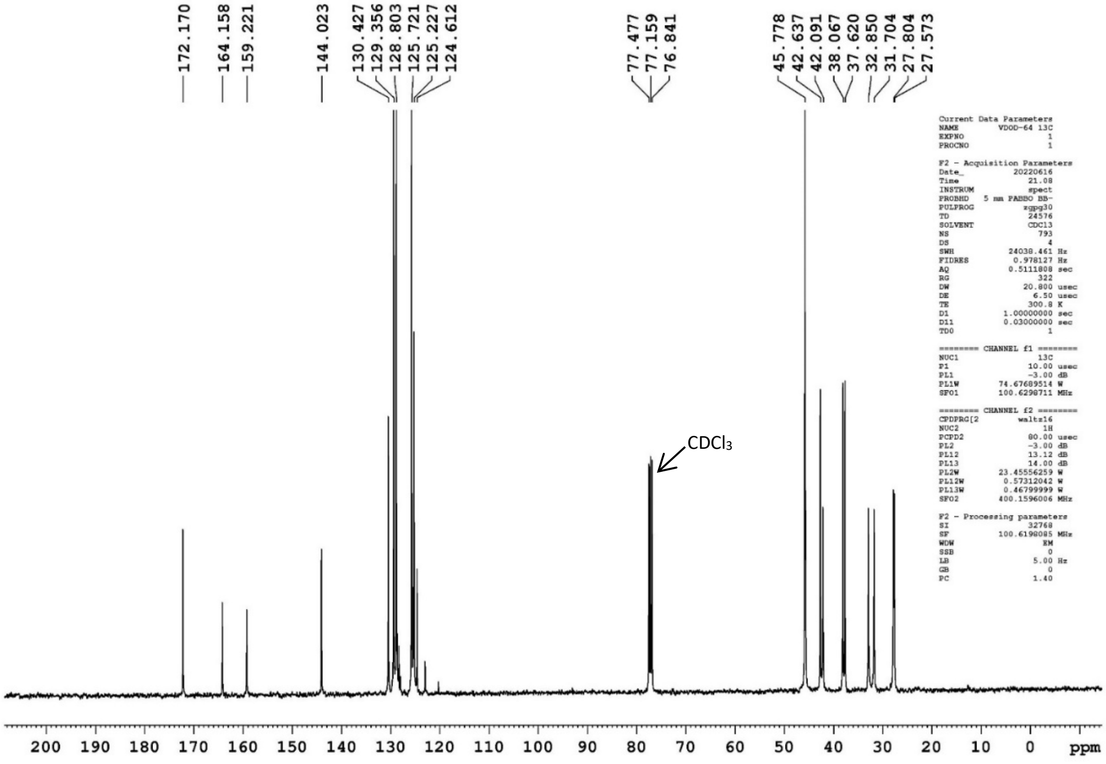

^{13}C NMR spectrum of (1-(5-phenyl-1,3,4-oxadiazol-2-*yl*)piperidin-4-*yl*)(4-(4-(trifluoromethyl)benzyl)piperidin-1-*yl*)methanone **(P30)**

HRMS, HPLC and LRMS of P30

Single Mass Analysis
Tolerance = 5.0 mDa / DBE: min = -1.5, max = 100.0
Element prediction: Off
Number of isotope peaks used for i-FIT = 3

Monoisotopic Mass, Even Electron Ions
76 formula(e) evaluated with 1 results within limits (up to 50 closest results for each mass)
Elements Used:
C: 0-200 H: 0-200 N: 4-4 O: 0-20 F: 3-3
VDOD-09JAN23-64 279 (4.736) AM2 (Ar,25000.0,0.00,0.00); ABS
TOF MS ES+

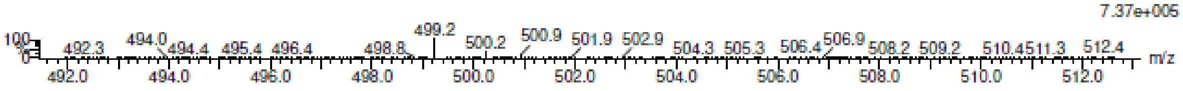

Minimum:				-1.5				
Maximum:		5.0	5.0	100.0				
Mass	Calc. Mass	mDa	PPM	DBE	i-FIT	Norm	Conf(%)	Formula
499.2314	499.2321	-0.7	-1.4	13.5	412.5	n/a	n/a	C27 H30 N4 O2 F3

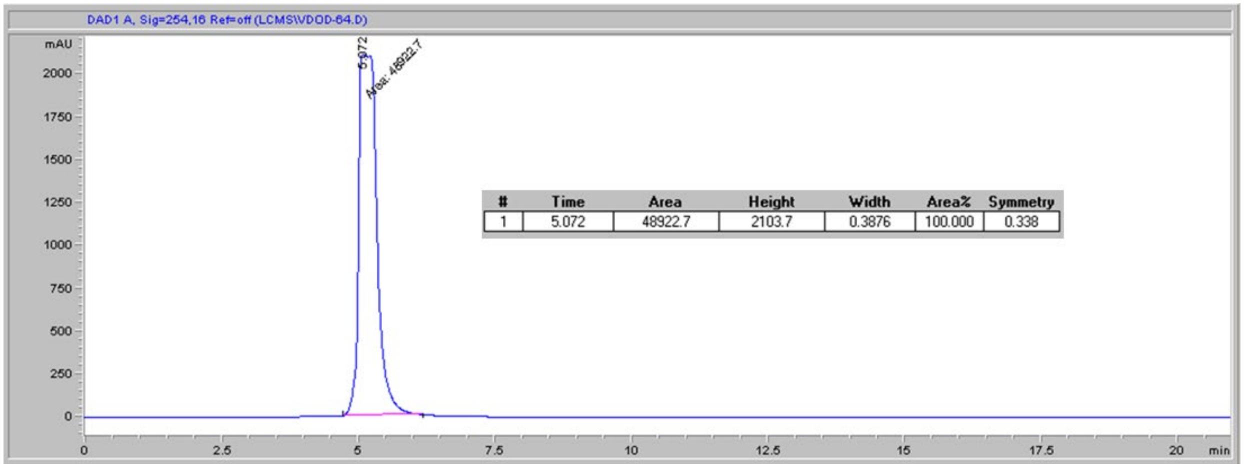

#	Time	Area	Height	Width	Area%	Symmetry
1	5.072	48922.7	2103.7	0.3876	100.000	0.338

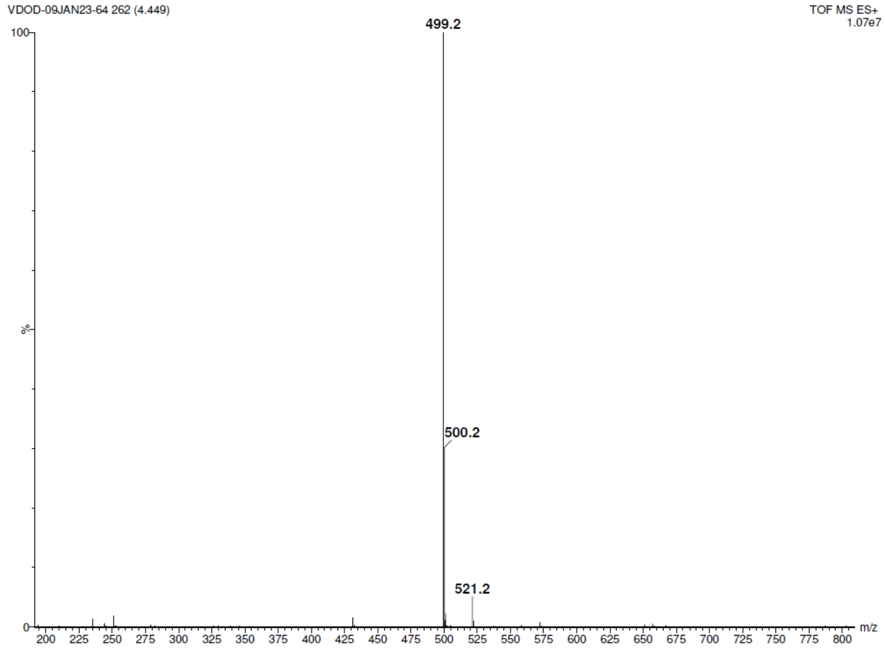

Supplement: Supplementary file 1 — ml3c00295_si_001.pdf [file ml3c00295_si_001.pdf]
